# Chapter II: Symptoms of the Head

**Published:** 1889

**Authors:** Edmund J. Lee


### CHAPTER II.

### SYMPTOMS OF THE HEAD.

**Aching**, undefined pain: abrot. absin. acet-ac. acon. æsc-h. æth. agar. ailan. all-c. aloe. alum. ambr. am-c. anac. ang. ant-c. ant-t. apis. apoc. arg. arg-n. arn. ars. arum-t. asaf. asar. aur. bad. *bapt.* bar. *bell.* benz-ac. berb. bism. bov. brom. *bry.* bufo. cact. calad. calc. calc-p. camph. cann i. cann-s. canth. carb-an. carb-v. cast. caul. cham. chel. chin. chin-s. cimic. cina. cinnb. clem. cob. coca. *cocc.* cocc-c. coff. colch. *coloc.* com. con. conv. corn. creos. croc. crotal. croton. cund. cupr. cupr-ac. cyc. daph. dig. *dios. dros.* dulc. elaps. elat. eugen. eup-per. euph. euphr. eupi. ferr. ferr-iod. fluor-ac. gamb. gels. gent. gins. *glon.* gran. graph. grat. guai. gymn. ham. hell. hepar. hipp. hura. hydr. hydr-ac. hydrphb. hyos. hyper. *ign.* indg. indm. iod. ipec. *iris.* jalap. jatr. *k-bi. k-ca.* k-clc. k-iod. kalm. lach. lachn. lac-ac. lact. laur. led. lil-t. lobel. lyc. mag-c. mag-m. mag-s. manc. mang. meli. ment-pi. meph. *merc.* merc-c. merc-i-fl. merc-i-r. merl. mezer. mill. morph. mosch. murx. mur-ac. naja. nat-c. *nat-m.* nat-p. nat-s. nicc. nit-ac. nitr. nx-m. *nx-v.* œna. osm. oxal-ac. pallad. par. petr. phel. phos. ph-ac. phys. phyt. pic-ac. plan. plat. plb. podo. poth. prun. psor. ptel. *puls.* ran-b. ran-sc. raph. rheum. rhod. rhus. rhus-v. rumx. sabad. sang. secale. selen. seneg. *sep. sil.* spig. spong. stann. staph. stram. strych. sulph. sul-ac. tabac. tanac. tarax. tarent. taxus. tellur. thea. thu. tilia. tril. trom. urt-ur. ustil. valer. verat. viol-od. viol-tr. vip. xanth. *znc.* znc-s. zing. ziz. etc.

— — **alternating** with pain in abdomen: æsc-h. cina. *gels.* plb. rhus-r.

— — — with stitches in hypochondrium: *æsc-h.*

— — **alternating** with cough: lach.

— — — with diarrhœa: podo.

— — — with frightful dreams: chin.

— — — with hæmorrhoids: abrot. aloe.

— — — with lumbago: aloe.

— — — with pains in joints: sulph.

— — — with pain in loins: aloe. lycps.

— — — with pain in neck: hyos.

— — — with pain in teeth: *lycps.*

— — **extending** to chest: con. nat-m.

— — — to ears: lach. merc. puls. rhus.

— — — to eyes: asaf. brom. caus. croc. mag-m. nat-m. spig.

— — — to face: am-m. anac. arg-n. graph. guai. lyc. nat-m. phos. puls. rhus. sars. spig. tarent. thu.

— — — to finger tips, with trembling and uneasiness: camph.

— — — to jaws: bell. calc-p. k-clc. spig.

— — — through limbs: acet-ac.

— — — to neck: bar. berb. bry. chel. guai. jac. *lach.* lyc. merc. mosch. nitr. nx-m sabin.

— — — to shoulders: glon. graph.

— — — down spine: *cocc.*

— — — into teeth: creos. crotal. graph. hydrph. lyc. lycps. merc. mezer. puls. sil.

— — — to throat: tarent.

— — — to tongue: ipec.

— **brain**, aching deep in: *acon.* aloe. alum. am-c. anac. arg. arg-n. asaf. asar. bar. bell. bov. calc. camph. canth. carb-v. caus. cham. *chin.* cina. coloc. cocc-c. con. corn. croc. daph. dros. dulc. glon. graph. hyos. ign. lach. laur. lyc. mag-c. mang. merc. *mosch.* mur-ac. nat-m. nitr. nx-v. olnd. petr. phos. ph-ac. phys. prun. ran-b. rhod. ruta. sabad. sars. sil. *spig.* stann. staph. sulph. sul-ac. therid. thu. znc.

— — with expression of anguish or deep scowl, eyes closed: canth.

— **forehead**, aching in: acet-ac. acon. æsc-h. agar. ailan. *all-c. aloe. alum.* am-c. anac. ang. ant-c. ant-t. apis. arg. *arg-n.* arn. ars. arum-t. *asaf.* asar. *bapt.* bar. bar-ac. *bell.* berb. *bism.* borax. bov. brom. *bry.* bufo. cact. caj. *calc. calc-ac.* calc-p. camph. cann-s. canth. carb-an. carb-v. caul. caus. cham. *chel. chin.* chin-s. *cic.* cimic. cina. cinnb. cist. clem. cob. coca. cocc. cocc-c. coloc. con. corn. creos. croc. crotal. croton. *cyc.* diad. *dig. dios.* dros. dulc. elaps. elat. euph. euphr. *ferr.* fluor-ac. gels. gins. glon. gran. graph. grat. gymn. guai. ham. hell. helon. hepar. hipp. hura. hydr-ac. hydr. hydrph. hyos. *ign.* iod. indg. iris. k-bi. *k-ca.* k-clc. kalm. lac-can. lach. lachn. lac-ac. lact. laur. led. lil-t. lith. lyc. mag-c. mag-m. mag-s. manc. mang. merc. merc-c. merc-i-fl. merc-i-r. merl. mezer. mosch. mur-ac. naja. *nat-c. nat-m.* nat-s. nit-ac. nitr. nx-m. olnd. ol-jec. op. osm. oxal-ac. *par.* petr. phel. *phos.* ph-ac. phys. phyt. pic-ac. plan. plect. plb. podo. psor. ptel. puls. ran-b. raph. rheum. rhod. rhus. *rumx.* samb. *sang. seneg.* sep. sil. *spig.* spong. stann. staph. stram. stront. *sulph.* sul-ac. tabac. tarax. tarent. tellur. thea. therid. thu. tilia. tril. trom. ustil. valer. verat. verat-v. verb. viol-tr. xanth. znc. zing. etc.

— — **alternating** with pain in back: brom.

— — — with crampy pain in chest; at last tearing in nose and shoulders: lachn.

— — **extending** backwards: arn. bry. cupr. eup-per. k-bi. lil-t. phyt. prun. spong. therid.

— — — over whole head: anac. selen. valer.

— — — to cheeks: brom. lachn. mosch. sang.

— — — to eyes: ant-t. apis. asar. calc-p. cham. grat. ign. k-ca. lac-can. lac-ac. lach. mur-ac. nit-ac. nx-m. *phos.* puls. sabin. seneg. *spig.*

— — — to neck: kalm. lyc.

— — — to nose: calc. dios. mosch. phos. sep.

— — — to shoulder: kalm.

— — — to occiput: bell. bry. calc. cham. colch. dios. nat-c. nat-m. therid.

— — — to orbits: chel. gins. (r).

— — — to vertex: cimic. hell. ipec. ruta. valer.

— — **eyes**, aching above: acon. æth. agar. ailan. aloe. alum. ambr. am-c. ang. ant-c. *apis.* arg. arg-n. arn. ars. asaf. aspar. aur-m. bapt. bar-ac. *bell.* berb. *borax.* bov. brom. *bry.* cadm. calc. cann-i. canth. caps. carb-an. carb-v. caus. cedr. chel. chin. chin-s. cimic. cina. cinnb. cist. coca. colch. con. *croc.* crotal. croton. cund. dig. dios. dros. ferr. ferr-i. fluor-ac. *gels. glon.* gymn. ham. hell. hepar. hipp. hura. hydr. hydrph. hyos. hyper. ign. indm. iod. ipec. *k-bi.* k-ca. kalm. lach. lac-ac. lact. laur. lil-t. lith. lobel. *lyc.* mag-c. mang. marum. *meph.* merc. merc-i-r. merl. mezer. mosch. naja. nat-c. *nat-m.* nat-p. nit-ac. nitr. nx-j. nx-m. *nx-v.* ol-an. op. osm. oxal-ac. *petr. phos.* ph-ac. *phys.* phyt. pic-ac. plan. plat. plb. ptel. *puls.* ran-b. raph. rheum. rhus. rhus-r. sabad. *sang.* selen. seneg. sep. *sil.* sol-n. *spig.* spong. *stann.* staph. sulph. sul-i. tabac. taxus. tellur. tereb. therid. thu. urt-ur. *valer.* verat. viol-tr. zing.

— — — in a narrow line: bry.

— — — to occiput: bism. naja.

— — — to vertex: arg-n. gymn.

— — **right eye**, aching above: acon. æsc-h. agar. all-c. am-c. am-m. anac. ant-c. arg-n. ars. aur-m. bapt. bar-ac. *bell.* bism. bov. bry. carb-an. caus. *chel.* chin. chro-ac. cimic. cinnb. cist. coca. cocc. colch. com. croton. cyc. daph. dig. dios. dros. dulc. evon. ferr. fluor-ac. gins. glon. ham. hyos. *ign.* indm. iris. lac-can. lach. lyc. mang. merc-i-fl. mezer. mur-ac. nat-m. nitr. nx-v. op. phos. phys. phyt. plat. *ran-b.* rhus. rumx. *sang.* spig. staph. stront. sulph. tabac. tarent. tong. viol-tr. xanth. znc. zing. ziz.

— — — first right then left: calc. lac-can. ptel. sep. sin-n.

— — **left eye**, aching above: acon. æsc-h. æth. all-c. ambr. am-c. ant-c. ant-t. arn. *ars.* arum-t. asaf. bar. berb. bov. brom. *bry.* caj. calc-p. camph. cann-i. caus. cedr. chel. cimic. colch. dios. euph. ferr. glon. ham. hell. hydr. hydrph. ign. indm. k-bi. *k-ca.* kalm. lac-can. lach. lil-t. lyc. mag-c. mag-s. men. merc-c. merc-i-r. mosch.

mur-ac. naja. nat-m. nat-p. nit-ac. nx-j. nx-m. nx-v. oxal-ac. phos. ph-ac. plat. puls. ptel. rhus-r. *sep.* sol-n. *spig.* stann. stram. sulph. sul-ac. tellur. tereb. verat. verat-v. verb. znc. zing.

— — — first left then right: lach. zing.

— — — thence to occiput and finally over whole body: *bry.*

— — — then over whole forehead, increasing and decreasing gradually: stann.

— — **behind orbits**, aching: acon. bad. berb. bism. cann-i. chel. cimic. daph. dig. gels. lach. nitr. poth. rhus. seneg. *therid.* ziz.

— — **between eyes**, aching: cupr. lach. poth.

— — **nose**, aching above root of: *acon.* agar. am-c. ant-t. ars. bar. bism. borax. calc-p. camph. coloc. dig. guai. hepar. ign. k-bi. mosch. nx-v. plat. puls. raph. staph. stict. viol-tr.

— — — left half: mur-ac.

— — **both sides** of forehead: carb-v. glon. jatr. k-bi. lac-ac. ruta. sul-ac. valer. verb.

— — **right side** of forehead: acet-ac. *acon.* agar. aloe. anac. ant-t. apis. arg-n. arn. ars. arum-t. asaf. bar. bell. berb. bov. brom. bufo. canth. cast. chel. chin. cimx. cinnb. cocc. cocc-c. colch. creos. crotal. cyc. dig. dios. dros. euph. ferr. ferr-i. fluor-ac. glon. grat. hell. *hepar.* hydrph. ign. indg. iod. k-bi. kalm. lach. *laur.* marum. men. merc. merc-i-fl. *mezer.* mosch. nat m. nat s. nicc. ol-an. op. osm. phel. phos. pic-ac. prun. psor. *ran-b.* ratan. rhod. rhus-r. rumx. ruta. sabad. sabin. sang. sars. seneg. sep. sil. spig. spong. squil. stann. staph. stram. sulph. sul-ac. tarent. thu. urt-ur. valer. verb. znc.

— — — **extending** to left side: acet-ac. æth. cyc. ign. iris. sabad.

— — — — to cheek: lachn.

— — — — to occiput, through head: *prun.*

— — **left side** of forehead: acet-ac. acon. æth. agar. ant-c. ant-t. apis. arg. arg-n. arn. asaf. *asar.* aur. aur-m. bell. bov. bry. cact. camph. carb-an. caus. chel. chin. chin-s. cic. cina. clem. coca. cocc. colch. coloc. creos. cund. cupr. dulc. euph. evon. fluor-ac. glon. gran. grat. hæmat. ham. hipp. hydrph. hyos. iod. ipec. k-bi. kalm. lac-ac. laur. lil-t. lith. lyc. mag-c. mag-m. men. merc. mezer. mur-ac. nat-c. nat-m. nat-s. nitr. ol-an. op. par. phel. ph-ac. phys. plan. plat. prun. psor. ptel. rhod. rhus. sabin. sars. seneg. sep. sil. spig. spong. stann. staph. sulph. sul-ac. tabac. tarax. *thu.* tong. valer. verb. znc.

— — — **extending** to right side: agar. hæmat. rhus-r. squil.

— — — — to occiput: nat-c.

— — **occiput**, pain in forehead and: æth. agar. alum. ambr. anac. aphis. arn. asaf. aur. bell. bry. calc. camph. cann-i. canth. caps. carb-v. chel. chin. chin-s. cimic. cina. clem. colch. con. corn. dig. dios. eup-per. ferr. gels. glon. graph. grat. guai hydr-ac. hyos. ign. iod. iris. k-bi. k-ca. lachn. laur. lyc. mag-c. mag-m. mang. merc. mezer. mosch. mur-ac. nat-m. nitr. ol-jec. op. petr. ph-ac. prun. ptel. raph. rhus. sabad. sabin. sars. seneg. sep. serp. spig. spong. squil. sulph. sul-ac. tabac. thu.

— — in morning on waking pain in forehead and occiput: k-bi. lach.

— — **temples**, pain in forehead and: agar. agn. ant-c. ant-t. arn. ars. arum-t. atro. aur. bar-ac. *bell.* berb. bov. bry. *camph.* canth. cedr. chel. chin. chin-s. clem. coloc. coral. croton. cyc. diad. dig. dios. dulc. elat. ferr. fluor-ac. gels. glon. gran. hell. hipp. hura. hydr-ac. indm. iris. k-bi. kalm. lachn. lil-t. lyc. mag-m. mag-s. mang. merc-i-fl. merl. mezer. mur-ac. myric. naja. nat-m. op. phos. ph-ac. phys. phyt. pip-m. psor. rhod. sabad. sabin. selen. seneg. spig. stann. sulph. tabac. tanac. verat. znc.

— — **temples and occiput**, pain in forehead: acon. æsc-h. bov. cann-s. nx-v. rhus-r. spig.

— — **vertex**, pain in forehead and: acet-ac. acon. all-c. aloe. ambr. anac. ant-c. ant-t. arg-n. bar-ac. bell. berb. borax. bry. bufo. calc. cann-i. carb-an. cast. caus. cinnb. corn. croton. dig. dios. glon. graph. grat. helon. hura. hydr-ac. hydrph. ign. indg. k-bi. laur. mag-c. mang. men. merc. mezer. mosch. mur-ac. myric. naja. nat-c. nat-m. nx-v. ol-an. ol-jec. oxal-ac. phel. puls. rhus-r. sep. sil. sol-n. stann. valer. znc.

— **neck**, pain spreading from: bell. berb. carb-v. ferr. fluor-ac. *gels.* glon. lac-can. puls. sang. sil. verat-v.
— — pain coming up through base of brain: gels. kalm. sang. sil.
— **occiput**, aching pain in: acon. *æth.* ailan. agar. *all-s.* alum. ambr. anac. apis. *ars.* asar. bapt. bar. bell. bism. bov. *bry.* cact. *calc.* calen. camph. cann-i. caps. carb-an. CARB-V. caus. cedr. cham. chel. *chin.* chin-s. cimic. cinnb. clem. cob. coca. cocc-c. colch. coloc. con. conv. cop. corn. crotal. croton. cyc. daph. dig. dios. dulc. elaps. euph. ferr. ferr-p. fluor-ac. *gels.* glon. gnap. *graph.* grat. ham. hydr-ac. *hell.* hydrph. hyos. hyper. hura. ign. indg. indm. iod. ipec. k-bi. k-bro. k-ca. k-clc. lac-can. lach. lachn. lac-ac. lact. laur. lil-t. lith. lobel. lyc. mag-c. mag-m. mag-s. mang. merc. merc-i-fl. mezer. mill. mosch. murx. mur-ac. myric. nat-c. nat-s. nicc. nit-ac. nitr. NX-V. op. osm. pæon. petr. phos. ph-ac. phys. phyt. pic-ac. pip-m. plan. plat. plect. plb. prun. ptel. puls. ran-b. ran-sc. raph. rhod. *rhus.* *rhus-r.* rumx. sabad. sabin. sang. secale. *seneg.* sep. *sil.* spig. spong. squil. stann. staph. stram. stront. stry. sulph. tabac. tarent. thu. tilia. trom. urt-ur. verat. verat-v. verb. xanth. zinc. znc-m.
— — **alternating** with pain in forehead: mosch.
— — — with pain in joints: sulph.
— — **extending** down back: æth. cimic. graph. lil-t. nat-m. pic-ac. podo. strych.
— — — to chest: graph.
— — — to ears: chel. æsc-h. plb.
— — — to eyes: atro. gels. *glon.* petr. *sang.* (r), sars. *sil.* (r), *spig.* (l).
— — — to forehead: arg-n. aur. bell. bov. brom. chel. clem. dios. ferr. fluor-ac. *gels.* *glon.* k-bi. nat-m. op. petr. ph-ac. plb. ptel. seneg.
— — — over whole head, from morning till afternoon: chin.
— — — to lower jaw: cham.
— — — from right to left: dig. staph.
— — — from left to right: squil.
— — — to neck: *bry.* glon. hell. k-ca. lil-t. phyt. sulph. ziz.
— — — down back of neck: cimic. lobel. podo.
— — — to shoulders: bry. gels. ipec. podo.
— — — — — while lying on back in bed, after waking: bry.
— — — to temples: *glon.* coca. plb. seneg. *spig.* (l).
— — — upward: glon. ph-ac. *sang.* sep.
— — — to vertex: *calc.* CIMIC. glon. hura. lac-ac. nat-c.
— **semi-lateral** pains in head: acon. agar. agn. *alum.* ambr. am-c. am-m. *anac.* ang. ant-c. ant-t. apis. arn. ars. *asaf.* *asar.* aur. bar. *bell.* bism. borax. bov. bry. *calc.* calc-p. camph. cann-s. canth. *caps.* carb-an. carb-v. caus. *cham.* chel. chin. *cic.* cina. clem. cocc. *coff.* colch. *coloc.* con. cop. creos. croc. cupr. cyc. dig. dios. dros. dulc. eugen. euph. euphr. ferr. glon. graph. guai. hell. hepar. hyos. ign. iod. ipec. k-bi. *k-ca.* lach. lact. laur. led. lyc. mag-c. mag-m. mang. marum. men. merc. mezer. mosch. murx. mur-ac. nat-c. nat-m. nicc. nitr. nit-ac. nx-m. *nx-v.* olnd. par. petr. phos. *ph-ac.* phyt. *plat.* plb. *psor.* *puls.* ran-b. ran-sc. rheum. rhod. *rhus.* ruta. sabad. sabin. samb. *sang.* *sars.* selen. seneg. *sep.* serp. sil. *spig.* spong. squil. stann. staph. stram. stront. sulph. *sul-ac.* tarax. tarent. thu. ustil. valer. verat. verb. viol-od. viol-tr. znc.
— — **alternating** with pain in left arm: ptel.
— — **coffee**, from excessive use of: nx-v.
— — **ears**, behind the, pain: asar. calc-p. chel. sang.
— — — behind the left ear: ambr. sang.
— — **extending** to eye: *asaf.* brom. caus. croc. mag-m. nat-m.
— — — to neck: guai. *lach.* lyc. merc.
— — — to neck and shoulders, neck stiff: lach.
— — — from side to side through temples: alum. chin. phos. plant. sang.
— — — to waist: hydrph.
— — **spots**, pain in: k-bi. kalm.
— **both sides** of head, simultaneously, pain in: all-c. asar. bov. calc-p. carb-an. chin. cupr. cyc. dig. dios. euphr. glon. lyc. mag-c. mag-m. merc-c. merc-i-fl. mezer. ol-an. phos. plat. squil. tilia. tong.
— — **alternating** from one to the other: agar. bell. colch. cupr. evon. hell. hydr. *lac-can.* lil-t. plan. valer.
— — on side on which one lies: calad. graph. mag-c. ph-ac.
— — ceases on one side, becomes more violent on other: lac-can. nat-m.
— **right side** of head, pains in: acon. agar. agn. alum. am-c. am-m. anac. ang. ant-t. apis. arg. arg-n. arn. ars. asaf. aur. bar. *bell.* bism. borax. bov. bry. bufo. *cact.* calc. camph. cann-i. caps. carb-an. carb-v. caus. cham. chel. chin. cic. cimic. cina. cinnb. cist. clem. coca. cocc. coff. colch. coloc. con. creos. croc. crotal. cyc. dig. dros. dulc. elaps. euph. euphr. gels. gins. gran. graph. grat. guai. hell. hepar. hyos. ign. indm. iod. jac. k-bi. k-ca. kalm. lach. laur. led. lyc. mag-c. mag-m. manc. mang. marum. men. merc. merc-i-r. mezer. mill. mosch. mur-ac. nat-c. nat-m. nitr. nit-ac. nx-m. nx-v. ol-an. petr. phos. ph-ac. plat. plb. puls. ran-b. rheum. rhod. rhus. ruta. sabad. sabin. *sang.* sars. seneg. sep. sil. spig. spong. squil. stann. staph. stront. sulph. sul-ac. tarax. tarent. thu. valer. verat. verb. znc.
— — right then left side: arn. bry. colch. cupr. dig. merc-i-r. staph. taxus.
— — right side, morning; left side, evening: bov.
— **left side** of head, pain in: acon. agar. agn. aloe. alum. ambr. am-c. am-m. anac. ang. ant-c. ant-t. apis. arg. arg-n. arn. *ars.* asaf. asar. aur. bar. *bell.* bism. borax. bov. brom. bry. calad. calc. calc-p. camph. cann-i. cann-s. canth. caps. carb-v. caus. cham. chel. chin. chin-s. cic. cimic. cina. cinnb. clem. cocc. colch. coloc. con. conv. creos. croc. crotal. cupr. cyc. dig. dios. dros. dulc. elaps. eup-pur. euph. euphr. *ferr.* fluor-ac. gent. graph. guai. gymn. ham. hell. hydr. hyos. ign. indm. iod. k-bi. k-ca. kalm. lac-can. *lach.* lac-ac. laur. led. lil-t. lith. lyc. mag-c. mag-m. manc. mang. marum. merc. merc-i-fl. merc-i-r. mezer. mur-ac. murx. nat-c. nat-m. nitr. nit-ac. nx-m. nx-v. olnd. pallad. par. petr. phel. phos. ph-ac. plan. plat. plb. ptel. puls. ran-b. ran-sc. rhod. rhus. sabad. sabin. samb. sang. sars. secale. selen. seneg. *sep.* *spig.* spong. squil. stann. staph. stront. sulph. sul-ac. tabac. tarax. thu. trom. valer. verat-v. verb. viol-od. viol-tr. xanth. znc. zing. ziz.
— — left then right side: arn. eup-per. glon. nx-m. squil.
— — of head and face extending to neck: guai.
— **temples**, aching in: acon. all-c. ambr. apis. apoc. arg. arn. ars. arum-t. aspar. atro. aur. *bad.* bapt. benz-ac. berb. brom. bry. calc-p. camph. cann-i. cann-s. caps. carb-an. caus. cedr. *cham.* chin. chin-s. cic. cina. cinnb. clem. cob. cocc-c. *coloc.* con. conv. coral. creos. crotal. croton. cup-ars. cyc. daph. dios. dros. elaps. elat. euphr. eupi. fluor-ac. gels. gins. glon. graph. ham. hell. *hipp.* hura. hydrph. hydr. hyos. hyper. ign. iod. iris. jatr. k-bi. k-ca. k-clc. kalm. lach. lachn. laur. led. lith. lobel. lyc. mag-c. manc. *merc.* merc-c. merc-i-fl. merl. mezer. naja. nat-c. nat-m. ol-jec. *op.* osm. phos. ph-ac. phys. phyt. pic-ac. plan. plb. podo. psor. ptel. raph. rheum. rumx. sabad. sang. *sep.* stram. tabac. tarent. *thea.* xanth. znc. zing.
— — **alternating** with heat of face: cocc-c. (1).
— — **blood-vessel**, as if a, had been torn out: æth.
— — **deep-seated**: eup-per. hydr-ac. naja. peti.
— — **extending** backward over ears: cedr. gymn..nat-p.
— — — to eye: asim. berb. cedr. cocc-c. nat-p. pip-m.
— — — to eyebrows: pic-ac.
— — — over forehead: *all-c.* ferr. glon. hepar. lil-t.
— — — to centre of head: dirc.
— — — to jaw, aggr. by change of weather: calc-p.
— — — to neck: pic-ac.
— — — to nose: glon.
— — — to occupit: cund. lil-t. meni.
— — — to shoulder, face distorted: graph. (1).
— — — to teeth, last molar: hydr. (r).
— — — from right temple to left: glon. lil-t. plat. ptel.
— — — from left temple to right: aur-m. calc. hipp. merc-i-fl. ol-jec. ptel.
— — — from temple to temple: alumn.

con. glon. ham. hydrph. lac-can. lil-t. lobel. mezer. naja.
— — — — — — — — and back again: hydr. *lac-can.* lil-t.
— — — toward vertex, amel. after cold washing: cocc-c.
— **vertex**, aching in: acet-ac. acon. æth. agar. agn. ambr. am-c. *anac.* ant-c. ant-t. apis. arn. ars. arum-t. bad. bell. borax. bry. bufo. *cact.* calc. calc-p. cann-s. carb-an. *carb-v.* cast. *caus.* cedr. cham. chel. *chin.* *cimic.* cinnb. cob. coca. cocc. cocc-c. coff. colch. con. conv. corn. creos. crotal. cupr. daph. dig. dios. dros. dulc. elaps. euph. eup-per. ferr. gels. glon. gran. graph. hell. hepar. hura. *hydr.* hydrph. hyper. iod. iris. k-bi. kalm. lac-can. *lach.* lac-ac. lact. laur. lith. *lyc.* men. merc. merc-c. merc-i-fl. *mezer.* mosch. mur-ac. naja. nat-m. nit-ac. nx-m. *nx-v.* ol-jec. oxal-ac. pallad. par. phos. phys. phyt. pic-ac. podo. ptel. puls. ran-sc. rheum. rhod. rumx. sabad. sang. sep. sil. sol-n. spig. spong. squil. stann. staph. stram. *sulph.* sul-i. tabac. tellur. thu. ustil. valer. verat. verb. xanth. znc.
— — **extending** backward: chel. k-bi. nitr.
— — — ear, from one to other: pallad.
— — — to eyes: ign.
— — — to forehead: caps. *caus.* cham. nx-m.
— — — to malar bones: tarent.
— — — to neck: calc-p. *chel.* glon. kalm.
— — — to occiput: calc-p. chel. gels.
— — — to temples: caus. cham. hipp.

**Agitation.** See Undulation, Vibration, etc.

**Air**, or wind passing through head, sensation as of: aur. colch. coral. mill. nat-m. petr. puls.
— extending to abdomen: aloe.
— on rocking: coral.

**Alive**, sensation as if something were, in head: ant-t. asar. croc. crotal. hyper. petr. sil. sulph.
— as if brain were an ant-hill: agar.
— as if everything in head were: petr.
— crawling, in forehead, as of a worm: alum.
— pressing, crawling pain, spreading out from centre, as of something alive: tarax.
— night: hyper.
— on walking: sil.

**Anxiety** in head: ant-c. apis. cic. laur. nat-m.

**Arthritic**: acon. arn. ars. *asar.* aur. *bell.* benz-ac. bry. camph. *caps.* caus. cham. chin. *cic.* *coloc.* con. *eugen.* graph. guai. hyos. ign. ipec. mang. mosch. nat-c. *nat-m.* *nit-ac.* *nx-v.* petr. phos. plat. puls. rhus. sabin. sep. *verat.* znc.
— periodical: bell.
— afternoon: aur. coloc.
— evening: eugen.

**Ascending** pains: meph: *ph-ac.* rhus. sang. sep. sil. staph.
— — See also special pains and parts from which pain extends.

**Asleep**, sensation in, as if: apis. carb. an. con. cupr. merc. mur-ac. nit-ac-op. sep.
— — after a debauch: *op.*
— — after eating: con.
— — when lying down: merc.

**Back**, pains extending as far as the: anac. bell. bry. calc. caus. dig. lyc. mag-c. nat-m. nit-ac. petr. phos. prun. *puls.* rhod. rhus. samb. sep. sil. spig. spong. stann. stront. sul-ac. thu.
— pains proceeding from the: *calc.* hell. sep. sil.

**Balancing** sensation in: æsc-h. bell. *glon.* lyc.
— difficult to keep the head erect: glon.
— pendulum-like: cann-i.
— on motion: crotal. fluor-ac. lyc. rhus.

**Ball**, sensation as of a, rising up: acon. plat. plb.
— — — — fast in brain: staph.
— — — — rolling in brain: hura. hydrph.

**Band.** See under Compressive.

**Beats** against bed: hyos. mill.
— against wall, in sleep: mag-c.
— feels as if he could beat head to pieces: nit-ac.
— Compare also with Pulsation, Striking, Throwing, etc.

**Beaten** sensation. See under Bruised.

**Bend** head backward, must: nitr.
— See under Conditions and compare with Falling, Throwing about, etc.

**Biting** sensation: arg. bar. carb-v. cham. grat. k-bi. k-iod. lyc. mezer. phel. ran-sc. rhod. secale.

— See also section upon External Head.
— rubbing aggr.: staph.
— scratching amel.: grat.
**Blindness**, followed by violent headache; sight returns as headache becomes worse: k-bi.
— followed by violent headache: gels.
— pain begins with blurred vision: iris.
— See also Eyes, under Concomitants.
**Blows**, pain as from: æth. alum. ant-t. arn. bov. caus. hell. indg. led. mang. nat-m. nx-v. olnd. ph-ac. plat. ran-b. ruta. sabad. spig. sul-ac. valer. znc.
— on back of head and neck: cann-i.
— on forehead, awakens at 1 A. M.: psor.
— on occiput: hell.
— vertex, stupefying pain as from a blow on: valer.
**Board** or bar before, sensation as of: acon. æsc-h. calc. carb-an. cocc. dulc. eugen. helon. lyc. olnd. op. plb. rhus. sulph. znc.
— before forehead: carb-an. cocc. helon. lyc. op. rhus. sulph.
**Boil**, pain as from a, forehead: hepar.
**Boiling** sensation: acon. alum. caus. chin. coff. dig. graph. grat. k-ca. laur. mag-m. mang. merc. sars. sil. sulph.
— as of boiling water: acon. indg.
— seething sensation in left side of vertex: lach.
**Bolt**, pains as from. See Nail, Plug, and compare with Pulsation, Shocks, etc.
**Bones**, pain in. See under section for External Head.
**Bores** head into pillows: apis. bell. camph. dig. hyper. stram.
**Boring**, digging, screwing pains: agar. am-c. am-m anac. ang. arg-n. ant-c. ant-t. aur. bar. bell. bism. borax. bov. bry. cadm. calc. camph. cann-s. canth. carb-an. caus. cham. chin. chin-s. clem. cocc. coloc. dros. dulc. graph. hell. *hepar*. hipp. hyper. ign. ipec. k-ca. k-iod. lach. laur. led. lyc. mag-c. mag-m. mang. merc. mezer. mosch. mur-ac. nat-m. *nat-s.* nit-ac. nx-v. olnd. ol-an. pæon. petr. phel. phos. ph-ac. plat. puls. ran-sc. rhod. rhus. ruta. sabad. sabin. samb. seneg. *sep.* sil. *spig.* squil. stann. staph. stram. sulph. thu. valer. znc.
— — extending to nose: phos.
— — — from within out: dulc. puls. sep. znc.
— — twisting, screwing pain from right side of head to both temples; after going to bed, spreading over whole head; returns daily; after a walk, on entering room: sabad.
— **forehead**: agar. am-m. anac. ant-c. arg. arg-n. bar. bell. *bism.* bry. calad. calc. carb-v. chel. chin. chin-s. colch. coloc. dios. dros. dulc. hell. hepar. hydr-ac. ign. ipec. k-ca. laur. led. mag-m. mang. merc. mezer. mosch. nat-s. nicc. ol-an. phel. plat. ruta. sabad. sabin. *sang.* sep. *sil.* spig. squil. znc.
— — deep: dulc. nat-m.
— — **eyes**, over: arg-n. (l), asaf. cimic. (l), colch. (r), cup-ar. (l), dulc. ipec. laur. led. lyc. (l), mag-s. (l), ol-an.
— — **inwards**: bell. calc. cocc. k-ca.
— — **intermittent**: arg.
— — **nose**, above: bism. coloc. hepar. nat-m. *sulph.*
— — **outwards**: ant-c. bell. bism. bov. dros. dulc. ipec. sep. spig. spong. staph.
— **occiput**: agar. gels. hell. merc. mosch. nat-m. nat-s. nicc. ol-an. ph-ac. plan. ran-sc. sabin. spig. stann. stront. znc.
— — intense pain as if a bolt had been driven from neck to vertex, worse at each throb of heart: cimic.
— **side of**: ang. aur. arg-n. (r), arum-t. (r), bov. (r), bry. (r), chin (l), coloc. (r), cop. (l), eup-pur. hepar. k-iod. laur. mag-c. mag-m. mag-s. (l), nat-m. nat-s. (l), puls. stann. (r), znc.
— — changing to stitches in evening: bell. (r).
— — in spot: hepar.
— — outwards: bell.
— **temple**, in: acon. aloe. (l), alum. (l), ang. ant-c. apis. arg-n. bar. bell. (r), bov. bufo. calc. (l), camph. carb-an. carb-v. (l), cham. clem. coloc. (r), cyc. (r), dios. (l), dulc. grat. hepar. ipec. led. (l), mag-m. mang. mezer. mur-ac. nat-s. (l). ol-an. pæon. ph-ac. (l), psor. ptel. (r), rhod. (l), stann. stram. sulph. thu.
— **vertex**: agar. ang. bar. bell. chel. chin. colch. lach. mag-s. mosch. mur-ac. nit-ac. olnd. phos. ph-ac. puls. samb. spig. sulph.
— — in spots: borax. colch.
— — outward: staph.

**Conditions** of Boring pain.
— **morning** : arg-n. camph. cham. dios. hepar. hydrph. hyper. nicc. nx-v.
— — after awaking : apis. arg. arum-t. aur. mezer.
— **afternoon** : aloe. mag-s. nicc. sang. sep.
— **evening**: aloe. arg-n. coloc. hipp. mag-c. mag-m. nat-s. plan. sep. znc.
— — amel. in : nx-v.
— — in bed : mag-s.
— **night**, at : am-c. carb-v. clem. dulc. sulph.
— **air**, cold, amel. : phos. thu.
— **bed**, aggr. in warm, at night : arg-n.
— **bending** back, aggr : aur. mang.
— **chilliness**, during : *sang.*
— **coffee**, after : nx-v.
— **cooling** of head aggr. : carb-an.
— **coughing** aggr. : aur.
— **dinner**, after : znc.
— — during : am-c.
— **eating**, after : nx-v.
— **eyes**, closing the amel. ; sep.
— **heat** and cold aggr. : grat.
— — of the face, with : puls.
— **laying** forehead on table amel. : ang.
— **light** aggr. : nx-v.
— **menses**, during : calc. mag-c. sep.
— **mental** exertion, from : nx-v.
— **motion** aggr. : hepar. sep.
— — even of talking : dulc.
— **noise** aggr. : nx-v.
— **pressure** aggr. : bell.
— — amel. : hell. ipec. sep.
— **rest** amel. : sep.
— **rubbing**, on : ol-an.
— **sitting**, when : agar.
— **stooping**, on : hepar. merc. sep.
— — stooping amel. : rising up or bending backwards aggr. : mang.
— **waking**, on : cham.
— — amel. by sleep when sufficient : sep.
— **walking**, when : bufo. coloc.
— **writing**, when : dros.

**Brain**, pain deep in. See under Aching, and also under special pains.

**Bruised**, beaten sensation : acon. agar. alum. am-m. anac. ang. arg. ars. *aur.* bell. bov. camph. canth. caps. caus. cham. chin. cob. coff. con. cupr. *euph. euphr.* gels. glon. graph. gymn. *hell.* hipp. ign. indm. iod. ipec. lach. led. manc. merc. mur-ac. nat-c. nicc. nitr. *nx-v.* op. phos. plan. *plat.* puls. ratan. staph. sulph. sul-ac. tarent. *verat.* znc.
— **extending** to root of tongue ; nausea, worse out doors : ipec.
— as if crushed, shattered, beaten to pieces : acon. æth. ars. aur. bov. camph. cham. *chin.* coff. euphr. graph. hell. ign. ipec. merc. mur-ac. nx-v. phos. puls. rhus. sep. stront. sul-ac. verat.
— — pain as if brain would be dashed to pieces, morning on awaking, on rising pain goes off and is converted into toothache, afterward passes into small of back : ign.
— — in spots : ratan.
— **brain**, in : anac. aur. *chin.* coff. cupr. gels. glon. hell. ign. indm. iod. ipec. merc. mur-ac. *nat-m. nx-v.* phos. phys. phyt. plan. rumx. stann. tellur.
— — paroxysmal : *verat.*
— **forehead** : ang. ant-t. ars. bapt. carb-an. cob. coff. gels. glon. hepar. hipp. indg. iod. lil-t. mag-s. merc-i-fl. nat-m. plan. plat. *puls.* ran-b. *rumx.* sol-n. stann. sul-ac. thu.
— — breaking sensation, after dinner : nat-s.
— — as if on surface of brain : ph-ac.
— — as after violent blow : arn. chel. sol-n. sul-ac.
— — above eyes . cann-i. gels. plan.
— — above nose : carb-an.
— **occiput** : æsc-h. agar. alum. cann-i. chel. cic. coff. crotal. euph. gins. grat. hell. indg. merc-i-fl. mezer. nicc. nit-ac. nx-v. phyt. plan. sep. spig. tarent. znc.
— — — as if crushed : graph.
— — — extending to temples : tarent.
— **side** of : ars. benz-ac. bov. *chin.* con. crotal. grat. k-iod. laur. (l), mang-c. merc-i-fl. (r), nit-ac. (r), nx-v. plan. plat. (r), sil. sulph. (l).
— — — extending to orbit and teeth : crotal. (l).
— **temple** : atrop. cob. cop. (r), hæmat. nicc. (r), ph-ac. phys. plan. *rhus.* tarent.
— **vertex** : bov. chel. *gamb.* glon. hell. hyper. indm. iod. lach. *mag-c.* nicc. olnd. petr. ph-ac. phyt. sep. sil. thu.
— — in spots : *caus.* vinc.

**Conditions**, of Bruised pain.
— **morning** : aur. cob. gymn. hyper. nicc. nx-v. plan.
— — on waking : ign. *plan.* tarent.

— — on rising: ars.
— **afternoon**: nicc.
— **evening**: bov. euphr. graph. mag-c.
— **night**: cob.
— **air**, cold, aggr.: thu.
— — open amel.: ang. ipec.
— **bed**, on going to: plan.
— — in: aur.
— **coughing**, when: sulph. tarent.
— **dinner**, amel. after: rumx.
— **eyes**, on moving to affected side: con.
— — — — or opening: chin.
— — shutting amel.: plan.
— — on turning: cupr. mur-ac.
— **heat** in head, with: hell.
— **lying** down aggr.: aur. crotal.
— — amel.: alum.
— — on painless side amel.: nx-v. plan.
— **menses**, during: gels. mag-c.
— **mental** effort, from: anac. *aur.* chin.
— — reading, etc., aggr.: aur.
— **motion** aggr.: caps. *chin.* rumx. *tellur.*
— — moderate exercise amel.: mur-ac.
— **pressure** aggr.: cop. hell.
— **rest** aggr.: aur.
— **rising**, amel. after: *nx-v.*
— **rubbing** amel. temporarily. ars.
— **sitting** up in bed aggr.: mur-ac.
— **stepping** or jarring, from: phyt.
— **stooping**, when: ang. hell. nicc. rumx.
— **sun**, from exposure to: manc.
— **talking**, when: aur.
— **touch**, on: caus.
— **waking**, when: indm. plan. tarent.
— **walking**, when: caps.

**Bubbling** sensation in: asaf. bell. berb. bry. creos. indg. k-ca. nx-v. par. puls. *spig. sulph.*
— at night: par. puls.
— when walking: nx-v. spig.
— most relieved when leaning back when sitting: spig.
— in occiput: indg.

**Burning**: acon. ailan. am-c. ant-t. apis. arg. *arn. ars.* arum-t. aur. aur-s. bar. bell. bism. bov. bry. calc. *canth.* carb-an. carb-v. caus. chin. cocc. coloc. croton. cupr. dig. dulc. *eugen.* graph. hell. helon. ipec. k-bi. k-ca. lachn. lact. lil-t. lith. manc. mang. *merc.* mur-ac. nat-s. nit-ac. nx-j. nx-v. par. phel. phos. ph-ac. phys. plat. plb. psor. rhod. rhus. sabad. sang. secale. sep. sil. spig. stann. staph. stront. sul-ac. tabac. tarax. tarent. taxus. verat. znc-s.
— — compare with Heat in Head.
— — alternating with pain: brom.
— — as if brain were on fire: hydr-ac.
— — contracting: bism.
— — as from hot iron around head; or, hot water in: acon.
— — pressing: mang.
— — rest of body cool: *arn.*
— — in spots: ars. glon. graph. nit-ac. raph.
— — tearing: merc.
— **brain**, in: acon. arn. bell.
— — burning pain in and: phos.
— **forehead**, in: acon. *alum.* am-c. *ars.* aur. bism. bry. carb-an. carb-v. *caus.* cham. chel. chin. coloc. conv. croton. cupr. dulc. eup-per. glon. hydrph. hyos. ipec. k-ca. k-iod. lil-t. lyc. mag-m. mang. marum. men. merc. merc-i-r. mezer. mur-ac. nat-c. nat-m. nx-v. phos. phys. psor. rhus. rhus-v. sabad. secale. spig. stann. staph. stront. sul-ac. tarent. therid.
— — eyes, over: nx-m.
— — externally: grat. marum.
— — like fire: stann.
— — as if a hot iron were passed over it: grat.
— **occiput**: agar. apis. aur. aur-m. cupr. k-ca. indg. lyc. mag-m. nat-c. (r), picr-ac. rhus. sep. staph. sulph.
— — burning, stinging, beginning in: phos.
— **side** of: bapt. (l), bell. (r), calc. canth. mang. phos.
— — — ascending from neck, with soreness and giddiness: canth.
— **temples**: alum. (r), am-m. (l), apis. aur. (r), bar (r), cann-i. carb-an. (r), caus. (r), chel. (l), cimic. (r), cinnb. coloc. con. (r), croton. cupr. (l), merc. (l), nit-ac. (l), phel. phyt. plat. (l), rhus. (r), sabad. (l), sars (l), spig. (l), staph. (l), verb. (l), viol-tr. (r).
— — extending to cheek: mezer. (r).
— **vertex**: agar. arn. ars. bry. carb-v. caus. chin-s. coc-c. cupr. dulc. glon. graph. helon. hyper. *lach.* merl. nat-m. phos. ph-ac. ran-sc. raph. sabad. sep. stann. *sulph.* viol-tr. znc.
— — chilly: caus.
— — extending into temples, amel. by rubbing: phos.

— — in spots: *arn. graph.*
— — transient: nat-c.
**Conditions** of Burning.
— **morning**: arn. canth. *nx-v.* phos. phys.
— — on waking: cocc-c.
— **noon**: *sulph.*
— **afternoon**: canth.
— **evening**: am-c. merc-i-r. phys.
— — in bed: carb-v. merc. nat-c.
— **night**: arn. lyc. merc. sil.
— **air**, amel. in open: mang. myric.
— **chilliness**, with: ant-t. k-ca. sil.
— **climacteric**, during the: *lach.*
— **dinner**, after: *alum.* grat.
— **headache**, with: sang.
— **lying** on back, when: agar.
— — in bed aggr.: merc.
— — amel.: canth.
— **mental** efforts, from: sil.
— — employment amel.: helon.
— **motion** aggr.: apis. arn.
— — amel.: helon.
— **pressure** of hand amel.: apis.
— **rest** amel.: arn.
— **room**, on entering from air: *caus.*
— **rubbing** amel.: phos.
— **scratching**, after: ol-an. par.
— **sitting**, when: canth. phos.
— — upright amel.: merc.
— **sneezing** amel.: lil-t.
— **standing**, while: canth.
— **stooping** aggr.: apis.
— **touched**, when: ipec. nat-m.
— **vomiting**, after: nat-s.
— — amel.: eugen.
— **walking**, when: rhus.
— — amel.: canth.
— **wrapping** head up warmly amel.: *sil.*
**Burrowing** sensation: agar. ant-t. bar-ac. cham. clem. cocc-c. colch. eupi. hepar. mag-m. phos. ratan. (r), samb. *spig.* squil. tilia.
— **extending** from forehead to mouth: eupi.
— — to nose: phos.
— over eye, while walking: plat.
— in spots: inu.
**Conditions** of Burrowing.
— **morning**: agar. hepar.
— — after rising: bar-ac. junc. squil.
— **afternoon**: ant-t.
— **night**: agar.
— **air**, in open: agar. ratan.
— **bending** head, amel.: hepar.
— **dinner**, after: agar.
— **lying** amel.: junc. *spig.*
— **motion** and noise aggr.: *spig.*
— **walking** in open air: agar.
**Bursting**, splitting, etc.: æsc-h. am-c. *am-m.* ant-c. apis. arg-n. asaf. asar. bapt. bar. BELL. berb. bov. brom. *bry.* calad. calc. cann-s. *caps.* carb-an. cast. caus. cham. chel. *chin.* cimic. clem. cob. coff. con. creos. dig. dios. dolich. euph. ferr. gent. *glon.* graph. gymn. ham. hepar. hydr. hydroph. ign. k-ca. kalm. lach. lachn. lac-ac. lyc. mag-m. *merc.* mezer. mill. naja. nat-c. *nat-m.* nat-s. nicc. nit-ac. nitr. nx-m. nx-v. olnd. petr. phos. ph-ac. phys. pic-ac. prun. psor. ptel. puls. *ratan.* rhus. sabad. *sang.* sep. *sil. spig. spong.* stann. stront. *sulph.* sul-ac. thu.
— — compare with Enlarged, Fullness, Pressure, Pulsation, etc.
— — as if would fly to pieces: arg-n. asaf. bar. carb-an. caus. graph. hepar.
— — as if split open with a wedge: lachn.
— **brain**, would burst out: alum. con. *glon.* sol-n. verat.
— **forehead**: æth. am-c. ant-c. bar. *bell.* calad. calc. caps. chin-s. dulc. *ferr. gels.* graph. hell. hydr. indg. k-ca. lac-can. merc. nat-c. nat-s. nitr. nx-v. *olnd.* ratan. sep. sil. spig. spong. staph. ustil. znc.
— — **eyes**, over: k-bi.
— **occiput**: aloe. calc. spig. spong. staph. znc.
— — extending to top of head, is so severe she thinks head will burst and that she will go crazy: *calc.*
— — neuralgic, beginning in upper cervical region, extanding over head, causing bursting pain in forehead and eyeballs; worse at 10 A. M., when lying; nausea, cold sweat and cold feet: *gels.*
— **side of**: asar. brom. nicc. puls. znc. (r).
— **temples**: apis. bell. (r), cact. chin-s. cimic. glon. hell. ign. indm. ipec. kalm. lil-t. *sang.* sol-n. staph.
— **vertex**: am-m. bapt. calc. carb-an. *cimic.* graph. lac-ac. nat-s. nit-ac. sil. spig. spong. stront. xanth.
— — as if blown off: cham.
**Conditions** of Bursting.
— **morning**: am-m. dios. ham. lac-ac. phos.
— — on awaking: con. hydr. nx-v.

— — on first opening the eyes: *bry.*
— — beginning in and gradually increasing till evening: bry. sang. sep.
— **night**, worse at: cact. carb-an.
— **evening**: clem. ham. ratan.
— **day**, every: *sulph.*
— **air**, in open: *bell.* mag-c.
— **coughing**, when: dios. merc. nat-m. phos. ph-ac. sep.
— — aggr.: bell. hydr.
— **eating**, after: graph. nat-s. nx-v.
— — dinner, after: k-bi.
— **eyes**, aggr. on moving: chin. ptel. puls.
— — amel. on opening: chin.
— **fever**, with: æsc-h. bell.
— **influenza**, during: naja.
— **jar**, from any: bell. chin. sil.
— **lying** down amel.: lach. mag-m. sang.
— — or resting head amel.: k-bi.
— **menses**, during: berb. *bry.* calc. *glon.* lyc. nat-m. sang. sep.
— **mental** labor, from: arg-n. ptel.
— — reading or writing amel.: ign.
— **motion**, from: caps. chin. hydrph. k-bi. mag-m. rhus. sep. sol-n.
— — continued hard motion amel.: sep.
— **press** with hands, must: carb-an. glon. mag-m.
— **resting** head amel.: k-bi.
— **rubbing** amel.: phos.
— **sitting**, when: phos.
— — bent over, when: ratan.
— **sleep** amel.: sang.
— **stool**, after: ratan.
— **stooping**, when: ham. hepar. hydr. hydrph. k-bi. nat-m. ptel. sep. stry.
— **talking** aloud, when: ign.
— **turning**, after: hydrph.
— **waking**, on: cham. ham. hydr.
— **walking**, when: caps. k-bi. stront.
— — in open air amel.: *sang.*
— **weather**, worse in wet: carb-an.
— **weeks**, every six: mag-m.

**Buzzing**, humming in: acon. bar. calc. caus. cocc. coff. ferr. graph. hyper. k-ca. k-iod. lact. lyc. nat-s. nit-ac. nx-v. phos. ph-ac. plat. puls. rhus. sars. spong. squil. stann. staph. sulph. sul-ac. thu. verat. viol-tr.
— as of bees: carb-v. op.
— chirping as of locusts: bry.
— evening, as after a debauch: cocc.
— — in warm room: ph-ac.
— menses, during: brom. *creos.*
— water, as of boiling: bar.
— vertigo, during: nat-s.
— vertex, in at night: hyper.

**Cleaving** pain. See Cutting, Darting.

**Cloudiness**. See Confusion.

**Coldness**, chilliness, etc., of: acon. agar. almn. ambr. ant-t. apis. arn. ars. ars-iod. asaf. asar. bar. BELL. benz-ac. CALC. calc-p. chel. cimic. cist. coca. cocc. con. creos. dios. dulc. eup-per. gins. glon. graph. grat. ham. hura. indm. iod. k-ca. lact. laur. lyc. mag-m. mag-s. mang. merc. MERC-C. merc-i-r. morph. mosch. naja. nat-m. nit-ac. olnd. phel. phos. phyt. raph. RHUS. *ruta.* sabad. *sep.* STANN. staph. STRONT. strych. *sulph.* sum. tarent. thea. tilia. valer. *verat.* verb. vip. zing.
— — **air**, as from cold: acon. arg-n. laur. nat-m. petr.
— — **alternating** with heat: bell. calc. merc. verat.
— — **begins** in head: *bar.* nat-m. stann.
— — — spreads from the: mosch. valer.
— — icy coldness: agar. bar. *calc.* laur. valer.
— — **internally**: arn. bell. calc.
— — **spot**, as of a cold: sulph.
— — **water**, as from cold: *cann-s.* croc. glon. sabad. tarent.
— **brain**, as from a cloth spread over: glon.
— — a cold, creeping along convolutions: abrot.
— **forehead**: acon. agar. arn. ars. bell. camph. cedr. chin. cimic. cinnb. cist. coff. colch. gels. glon. hydr-ac. hyper. *laur.* lyc. mag-m. merc. mezer. mosch. œna. phel. ph-ac. plect. puls. staph. sul-ac. verat. znc.
— — **air**, cold, penetrated painfully: znc.
— — **externally**: cist. gels.
— — **hand**, as if cold, touched: hyper.
— — **metal**, cold, as from: cinnb.
— — **middle** of, in brain: bell.
— — **spot**, in small, as if by a cold finger: arn.
— — **water**, as from cold: tarent.
— **side of**: *asar.* bar. (r), *calc.* (r), cann-s. *con.* croc. k-bi. lach. lobel. phos. (l), tarent. verat. (r).
— — one sided lach.

— — in small spot as a drop of water: croc. (l).
— — — — — above ear: asar. (l).
— **occiput**: acon. agar. aloe. alum. berb. calc-p. cann-i. chel. chin-s. cocc-c. dulc. gins. nitr. nx-m. plat. phos. sil. thea. verat.
— — and over back, every evening: dulc.
— — as if frozen: nx-v.
— — like cool air, rising from navel: acon.
— —rising from neck: chel. sep.
— **temples**: berb. (r), gamb. merc-c. ol-an. ph-ac. plat. rhod. tarent. (r).
— **vertex**: agar. am-c. arn. *arum-t.* aur-m. k-ca. k-iod. *laur.* mang. myric. nat-m. plat. sep. sil. sulph. tarent. valer. VERAT.
— — **extending** to sacrum: acon.
— — icy coldness: agar. arn. *laur.* valer.
— — in spots: mang. sulph.
— — upper part feels cold and as if without covering: arum-t.
— — water, as from cold; aching in head: tarent.

**Conditions** of Coldness.
— **morning**: cedr. dios. lact. plect. tarent.
— **afternoon**: *arum-t.* gamb. gels. ol-an. valer.
— **evening**: alum. ars-i. creos. dulc. hyper. merc-c. stry. sulph. znc.
— **night**: cimic. lyc. mang. sep.
— **air**, in open: phos.
— — amel. in: sep.
— **arteries**, with throbbing of: aloe.
— **breakfast**, after: arn.
— **burning**, after: sulph.
— **congestion** of, with: glon.
— **covered**, when: mang.
— — — amel.: grat.
— **earache**, with: sep.
— **heat**, with: chin. puls.
— **menses**, during: ant-t. calc. mag-s. sep. sulph. *verat.*
— **motion**, on: almn. chel. sep.
— **pressure** of hat, from: valer.
— **rest,** amel.: chel. sep.
— **riding**, after: lyc.
— **room**, in warm: merc-i-r. tarent.
— **sitting**, when: mezer.
— **stooping** aggr.: alum. sep.
— **sweat**, with: merc-c.
— **vertigo**, with: lact. verat.
— **walking**, amel.: gins.

**Come off**, sensation as if top of head would: bapt. cham. cob. cupr-s. sang.
— — Compare with Bursting, Pressure, etc.
— — at every jar: cob.

**Commotion**, painless, in: caus.
— See Motion, Undulation, etc.

**Compressive**, Constrictive, Contracting sensation: *acon. æth.* agn. *alum. ambr.* anac. ang. *ant-t.* apis. arg-n. arn. *asaf.* asar. bell. bov. *bry.* bufo. cadm. calc. *camph.* cann-i. cann-s. carb-v. *caus.* cham. chel. chin. cimic. cic. cina. *cocc.* coloc. con. creos. croc. daph. dios. dulc. eugen. fluor-ac. gamb. gent. *graph.* grat. guare. hell. hipp. *hyos.* hyper. *ign.* indg. ipec. k-ca. k-iod. lact. lam. laur. lobel. lyc. mag-c. mag-m. mag-s. mang. *men.* merc. merc-i-fl. mosch. nat-c. nat-m. nat-s. nit-ac. nitr. nx-m. olnd. *par. petr.* phel. ph-ac. phys. pip-m. *plat.* plb. prun. psor. puls. ran-b. rhus. sabad. sabin. selen. sep. sil. spig. spong. stann. *staph.* stront. sul-ac. tabac. tarax. tarent. therid. thu. valer. verat. vip. znc.
— — Compare also with Cramping, Heaviness, Pressure, etc.
— — **alternately** with relaxation: calc. lac-can.
— — **armor**, as if in: cann-i. clem. croton.
— — **band** or hoop, as if in: acon. æth. ant-t. brom. camph. clem. cocc. cyc. gels. glon. guai. iod. ipec. lam. laur. marum. merc. nit-ac. op. osm. petr. phys. plat. sabin. sars. spig. stann. *sulph.* therid. ziz.
— — **boards**, as if compressed by two: ipec.
— — **hat**, as if in a tight: phys.
— — **net**, as if in a: nat-m.
— — **periodic**: phos.
— — **screwed**, as if. See Vise, below.
— — **sides**, from both: acon. arg. bell. bov. bry. camph. chin. cic. com. gamb. hell. lam. mag-m. mag-s. men. nat-m. prun. sabad. tarax.
— — — as from a screw behind each ear: oxal-ac.
— — — from behind and before: nx-m. spong.
— — — from all sides: *acon.* tarax.
— — **skull** feels smaller: grat.
— — **string** or cord, as if bound by:

asaf. cyc. graph. hell. iod. merc. mosch. nat-m. psor. *sulph.*
— — — like a ligature, from nape to ears: anac.
— — **vise**, as if in a: æth. agar. alum. am-m. ant-t. *arg-n.* atro. bar. bry. cadm. carb-v. caus. chel. cina. clem. cocc. euph. glon. graph. grat. mag-c. mag-s. merc. nat-m. nicc. nit-ac. olnd. op. petr. plat. *puls.* ran-b. ran-sc. ratan. rhus. sabad. sars. spig. stann. sulph. sul-ac.
— **brain**, as if bound up: æth. am-bro. ant-t. arg. bry. calc. carb-v. cham. *cocc.* colch. cyc. hyper. laur. mag-s. merc. nat-m. olnd. op. ph-ac. plat. prun. puls. rhus. sars. sil. *spig.*
— — as if membranes of, were too tight: op.
— — as by an iron helmet: crot-c.
— — as by a cloth: cyc.
— **forehead**: *acon.* æth. ailan. alum. ambr. anac. ant-t. apis. arn. ars. asaf. asar. bell. bism. bry. camph. cann-s. carb-an. caus. cham. coff. colch. dig. dulc. elat. fluor-ac. gels. graph. hæmat. ham. ign. ipec. iris. lac-can. lept. manc. mang. merc. mosch. naja. nat-m. nitr. nit-ac. nx-m. olnd. osm. par. phys. phyt. *plat.* plb. psor. puls. sabad. sep. sil. stann. staph. sulph. sul-ac. tarax. therid. valer. verat. verb.
— — **across**: arn. ham. lept. naja. phys.
— — **alternating** with expansion: tarax.
— — **band**, as from: æth. ant-t. *carb-ac.* cedr. *chel.* coca. helon. indg. iod. iris. lil-t. merc. mill. tarent.
— — **eyes**, over: æth. ars. asaf. bell. bry. borax. (1), colch. nx-m. puls. sang.
— — — from margins of orbits to temples: cann-s.
— — **intermittent**: arn. hyos. plat.
— — **narrow**, as if too: gels.
— — **nose**, over, root of: *acon.* bov. camph. mosch. nitr. spong. tarax.
— — **sides**, from both: agar. alum. chin. cina. lyc. spig.
— — — from front to back: spong.
— — — from behind and above: æth.
— **occiput**: all-c. *ambr.* am-m. anac. asar. *camph.* *chin.* colch. fluor-ac. *graph.* guare. hura. *ign.* lact. manc. mang. *merc.* nat-m. nitr. phel. pip-m. *rhus.* *stann.* *staph.* stront. sul-ac. tabac. tarent. tong.
— — as from band: psor.
— — from front to back: pip-m.
— — constrictive pain, part feels stiff, forcing to bend head back, amel. tying hair up: *nitr.*
— — extending into back and chest, noon: *graph.*
— — upwards: all-c. puls.
— **side** of: acon. (1), æth. am-m. arg-n. asar. bov. bry. calc. caus. coloc. com. dios. hura. mag-m. mag-s. pallad. phel. phos. rhus. sars. squil. sul-iod.
— — like a band tied around: dios.
— — as from a screw behind ears: oxal-ac.
— — screwed in, as if, better in open air: k-iod.
— **temple**: *absin.* acon. agn. ambr. anac. ant-t. arn. ars. *asar.* (1), bell. bov. cann-s. canth. carb-an. caul. cedr. *cham.* chel. chin. cimic. coloc. con. cyc. dig. elaps. elat. fluor-ac. glon. hell. hipp. k-bi. lach. lept. men. mezer. naja. osm. par. petr. ph-ac. phys. plat. plb. *puls.* sars. *squil.* stann. sulph. tabac. therid. thu. verb.
— — **crushed**, as if: caul.
— — **both sides**, from: ant-t. lyc. men. mezer. nat-m. ran-sc. rhus. sars. sum. tabac.
— — **screws**, as with: acon. arg-n. lyc. sabad.
— **vertex**: *acon.* anac. benz-ac. chin. colch. con. crotal. fluor-ac. *graph.* ign. ipec. k-bi. kalm. laur. mang. men. nitr. nx-m. phel. phos. phys. *sep.* spig. STANN. staph. valer.
— — **above** downwards, and from both sides: nx-m.
— — extending to forehead: ign.
— — both **sides**, from: men.
— — as by an elastic body: benz-ac.
— — as if bound: cyc. kalm.
— — like a band drawn tightly over, from ear to ear: ipec.

**Conditions** of Compressive.
— **morning**: bry. cham. gamb. graph. k-bi. sulph.
— — after rising: lyc. tong.
— **afternoon**: graph. mag-c. naja. nit-ac. phos.
— **evening**: anac. hyper. k-bi. phos. *rhus.* sep. sulph. tabac. tarent. valer.
— **air**, in open, aggr.: merc. valer.
— — — amel.: coloc. lyc.

— **breakfast**, amel. after: bov.
— **chill**, during: tarent.
— — with coldness all over: camph. stann.
— **coughing**, on: petr.
— **drinking**, aggr.: merc.
— **eating**, after: con. k-ca. nat-m. sep.
— **eyes**, amel. on closing: *chel.*
— **light**, from candle: cann-i.
— **looking** intently aggr.: *puls.*
— **menses**, during: *gels.* helon. iod. lyc. merc. plat. sulph.
— — before: hepar.
— **motion**, from: asar. carb-v. mezer.
— — amel.: op. sulph. valer.
— — in open air, amel.: acon.
— — changing position amel.: valer.
— **pressure** amel.: æth. men. thu.
— — — temporarily: anac.
— **reading**, when: agn.
— **rest**, when at, aggr.: sulph. valer.
— — amel. when at: hipp.
— **rising**, amel.: laur.
— **room**, on entering: bov.
— — warm room: acon. cann-i.
— — amel. in a: hepar. merc. valer.
— **running**, amel.: hipp.
— **sitting**, amel.: asar. hipp.
— — when aggr.: fluor-ac.
— **sleeping** on affected side, when: caus. (1)
— — aggr.: merc.
— **stooping**, aggr.: coloc. thu.
— — bending backwards, amel.: thu.
— **vomiting**, when: asar.
— — amel.: stann.
— **waking**, on: tarent.
— — from a nap: bry. nx-v.
— **walking**, when: ang. *asar.* *chin.* hipp. thea.
— — in open air.
— **wet** weather aggr.: sulph.

**Concussion** of brain: *arn.* bell. *cic.* hepar. hyper. led. merc. ph-ac. rhus. sep. sul-ac.
— in head. See Shock.

**Confusion**, cloudiness, muddled, stupid feeling, etc., in head: acet-ac. *acon.* æsch-h. *æth.* agar. agn. alcoh. all-c. aloe. alum. ambr. am-c. am-m. anac. ang. *ant-t.* apis. apoc. arg. arg-n. *arn.* *ars.* asaf. *asar.* aspar. atrop. aur. aur-m. bapt. bar. *bell.* benz-ac. *bism.* borax. bov. brom. BRY. calad. calc. calc-p. camph. cann-i. cann-s. canth. caps. carb-an. carb-v. cast. caus. cham. chel. chin. chin-s. cic. cina. clem. coca. cocc. cocc-c. coff. colch. coloc. com. con. corn. creos. croc. croton. cup-ac. cyc. diad. dig. dios. dros. dulc. elat. ether. eugen. eup-per. euphr. eupi. ferr. fluor-ac. gels. gent. gins. glon. gran. *graph.* grat. hæmat. hell. hepar. hipp. hydr. hydr-ac. hyos. hyper. hura. ign. indg. iod. ipec. jatr. k-bi. k-bro. k-ca. k-iod. kalm. *lach.* lact. *laur.* led. lil-t. lith. lobel. lyc. mag-c. mag-m. mag-s. mang. marum. men-pi. men. meph. *merc.* merc-c. merc-s. merl. mezer. mill. mosch. murx. mur-ac. naja. nat-c. *nat-m.* nat-s. nicc. nitr. nit-ac. nx-j. *nx-m.* *nx-v.* olnd. *op.* osm. par. petr. phel. phos. *ph-ac.* phys. plat. plb. plect. prun. psor. ptel. *puls.* raph. rheum. *rhod.* *rhus.* ruta. sabad. sabin. samb. sang. sars. secale. seneg. *sep.* serp. *sil.* spig. spong. squil. stann. staph. stram. stryc. *sulph.* sul-ac. tabac. tarax. tereb. therid. thu. tilia. tong. tril. valer. *verat.* verb. viol-od. viol-tr. vip. xanth. znc. zing.
— — compare with Dullness, Stupefaction of head, etc.
— — **alternating** with nausea: secale.
— — — with pain in kidneys: alum.
— — as if memory failed: cham. mezer. puls.
— **forehead**: acon. agar. all-c. alum. am-m. anac. apis. arg-n. asaf. bar-ac. bell. brom. *bry.* calc. cann i. cann-s. chel. chin. clem. cocc-c. coff. colch. coloc. croc. croton. cyc. ferr-i. gins. gran. graph. hæmat. hell. hipp. hura. hydr-ac. hyos. k-bi. lact. laur. led. lobel. lyc. manc. merc. mezer. nitr. nx-m. op. ph-ac. plat. raph. ratan. rheum. rhod. ruta. sabin. *sep.* staph. *sulph.* thu. tilia. valer. vinc.
— — **alternately** in either frontal protuberance: lact.
— **occiput**: all-c. *ambr.* arg-n. asar. bov. cann-s. carb-v. cham. colch. con. croc. croton. fluor-ac. k-clc. lobel. mill. nat-c op. phel. sep. spig. tong. znc.
— — extending over head: gels.
— — and forehead: mang. mezer. squil. sumb.
— **side** of: anac. arn. asar. *coloc.* con. (1), creos. croton. cyc. (r), fluor-ac. (r), hell. (1), hydr-ac. laur. op. rhus-r. spig. sulph. (1).
— — sudden, as from smoke: sul-ac. (r).

— **temples**: asar. (1), croton. cyc. k-bi. lith. nx-m. plan. spig. thu.
— **vertex**: all-c. chel. cocc-c. corn. euphr. eup-per. k-bi. lobel. merc. raph. sulph.

**Conditions** of Confusion.
— **morning**: acon. agar. aloe. alum. ambr. am-m. anac. ant-t. arg. arn. asaf. asar. aur. bar. bell. bism. bov. *bry.* bufo. *calc.* canth. caps. carb-an. carb-v. caus. cham. chin. chin-s. cic. clem. cocc. colch. con. euphr. graph. hyos. ign. iod. k-ca. lach. lact. lyc. mag-c. mag-m. merc. mill. mosch. murx. nat-c. nicc. nitr. nx-j. op. petr. phos. ph-ac. ran-b. ran-sc. *rhod.* rhus. ruta. samb. sars. seneg. sep. sil. squil. stann. staph. sulph. sul-ac. thu. tilia. verat. znc.
— — on waking: acon. anac. ant-t. arg-n. ars. bar. *bry.* calc. calc-p. carb-v. clem. cocc-c. euphr. hyper. ign. lyc. mag-s. merc. phos. rhod. ruta. sulph. tilia.
— — when rising: anac. arg-n. asar. aur. bell. bry. carb-v. cham. cic. cina. clem. cocc-c. corn. graph. ign. k-ca. lact. mag-c. mag-m. merc. merl. phos. ph-ac. raph. rhod. rhus. sabad. samb. sep. sil. sulph.
— — after rising, amel.: ant-t. mag-s. phos. rhus.
— **afternoon**: asaf. bry. calc. carb-v. cham. chel. chin. clem. coloc. croton. ferr. graph. hell. hyos. k-bi. k-ca. laur. nx-v. op. petr. phel. sabin. sep. sulph. znc.
— **evening**: aloe. am-c. ars. bar. bell. borax. bov. calc. carb-an. carb-v. cham. chin-s. cocc-c. cyc. diad. dig. dulc. euphr. ferr. graph. iod. ipec. k-ca. kalm. lyc. mezer. mill. nitr. nx-v. phos. ph-ac. ptel. puls. rhus. ruta. sars. sil. spig. stann. sulph. sul-ac. thu. valer. znc.
— **night**: anac. arg-n. calc. croton. lyc. phos. psor. ptel. raph. ruta. sep. sulph. tilia.
— — on lying down: brom. lil-t. rhus-r.
— — on waking: *glon.* merc-i-fl. phos. psor.
— **air**, in open: agar. colch. con. croton. hyos. nit-ac. nx-v. rhod. sulph.
— — — amel.: am-m. ant-t. bar. bell. bry. clem. cocc-c. croc. dulc. glon. hydr-ac. mag-m. mag-s. mang. men. merc. nat-c. par. phos. ratan. sulph.
— **bed**, when in: ambr. calc. phos. rhod.
— **beer**, from: bell. calc. chin. coloc. con. coral. croton. ign.
— **breakfast**, after: cocc-c.
— — before: calc.
— **bread**, aggr.: croton.
— **carousal**, after a: gran. nx-v.
— — See Intoxicated, as from.
— **catarrh**, during: graph.
— — as during: berb. chin.
— — from suppressed: lyc.
— **chill**, during cold stage: caps. cic. dros. hell. k-ca. nat-c. nx-m. plb. rhus. ruta. stram. viol-tr.
— **coffee**, after: arg-n. calc-p. mill.
— — amel.: coca. hipp.
— **coition**, after: bov.
— **cold** bath, amel.: calc-p. euphr.
— **colic**, during: coloc.
— **compression**, amel.: eupi.
— **coryza**, during: æth. cocc-c. hepar.
— — as if from: berb. chin. coloc. con. ol-an. sep. staph.
— **cough**, before paroxysm of: cina.
— **dinner**, after: arg-n. carb-v. euphr. nx-v. phos.
— **drinking**, after: bell. cocc. con. croc.
— **eating**, from: mag-m. olnd. ph-ac.
— — after: ambr. arg-n. bell. bufo. carb-v. caus. *cocc.* cocc-c. coloc. croc. cyc. euphr. ferr. grat. hyos. led. lobel. lyc. men. merc. mill. nat-c. nat-m. nit-ac. *nx-v.* op. petr. phos. ph-ac. puls. sabin. sep. sil. sulph. znc.
— — amel.: agar. apis. caus. lach. mezer. nx-j. phos.
— **eructations**, from: all-c. gent.
— — amel.: bry. sang.
— **exertion**, on: *calc-p.* nat-c. phos.
— **fever**, during the: arg. bry. camph. cham. cocc-c. dros. *hyos.* ign. ipec. laur. op. phos. puls. sep. valer. verat.
— **hat**, putting on, aggr.: ferr-i.
— **head**, with **congestion** of: acon. arg-n. bry. carb-an. carb-v. glon. hyos. lach. nat-m.
— — **drawing** in temple, from: stann.
— — with **fullness** of: acon. agar. calc. corn. croton. cyc. gymn. hyos. mag-m. merl. phos. sulph. sul-ac. therid.
— — with **heat** of: arg-n. bism. caus. clem. corn. cyc. hipp. hyos. jatr. k-bi. k-bro. lach. lact. laur. mang.

merc-c. op. osm. pæon. petr. phel. sulph. therid.
— — with **heaviness** of: acon. agar. alum. anac. arg-n. asaf. asar. atro. bar. berb. bov. bry. calc-p. cann-s. canth. cic. cimic. clem. cocc. coloc. con. corn. croton. dros. euphr. ferr. gins. glon. hæmat. hell. hepar. hura. hyos. ign. indg. jatr. k-bi. k-ca. k-iod. lach. lac-ac. lact. laur. lyc. mag-c. mag-m. mang. merc. merc-c. mosch. mur-ac. murx. naja. nat-c. nitr. nx-j. nx-m. pæon. petr. phos. phyt plb. puls. rhod. rhus. sabad. sabin. sars. stann. staph. sulph. sul-ac. tabac. thea. therid. tilia. znc.
— — with humming in: ph-ac.
— — moving the, on: mag-m.
— — on raising the: clem. nx-v.
— — resting the, on table, amel.: rhod.
— — on turning the: clem.
— — trembling sensation in, with: calc.
— — wagging of, with: sep.
— **heat** of body, during: cocc-c. colch.
— — see Fever, above.
— **intoxicated**, as if: acon. asar. bell. dig. grat. ign. laur. lyc. nitr. nx-v. phel. rhus. sabad. spong. tong.
— — as after being: acon. agar. am-m. ang. arg. bell. *bry.* camph. carb-v. chin. clem. cocc. coloc. coral. croc. k-ca. lam. laur. mosch. nat-m. nx-v. op. ph-ac. psor. puls. rheum. sabin. squil. valer.
— **lie down**, must: marum. nit-ac. phos.
— **lying** down, when: brom. cham. lil-t. mag-m. merc. rhus-r.
— **menses**, during: am-c. cocc. lyc. phos.
— — after: nat-m.
— **mental** exertion, from: ang. aur. calc-p. canth. carb-v. caus. cham. cocc. diad. evon. gels. hepar. iod. laur. mag-c. mag-m. nat-c. nat-m. *nx-m.* nx-v. olnd. petr. ph-ac. pic-ac. puls. ran-b. sep. sil. staph.
— — activity, with: dig.
— **motion**, from: acon. ambr. bell. bry. cob. ign. indg. lobel. mosch. nx-v. *puls.*
— — amel.: ferr.
— **nose**, on blowing: euphr.
— — bleeding of, amel.: cham.
— **palpitation**, during: glon.
— **pressure** in temples, during: ant-t. gran.
— — of hands, amel.: hydr.
— **reading**, when: ang. calc. cocc. ferr-i.
— **rest**, when at: arg. nat-c.
— **riding**, when: bry. sil.
— **rising**, when: aur. bov. bry. k-ca. laur. merc. nat-m. nat-s.
— **room**, in a: acon. am-m. ars. aur-m. bry. croc. mag-m. men. merc. nat-c. phos.
— — amel. in: caus. colch.
— — warm: acon. ph-ac.
— **sitting**, when: am-c. asaf. asar. bar. bell. calc. carb-an. caus. cic. colch. k-ca. mang. merc. nat-c. nit-ac. puls. rhus. sabad. sars. sep. sil. spig. sul-ac. thu. valer. verat.
— **sleeping**, after: ambr. anac. ars. bry. calc. carb-v. con. graph. hepar. op. squil.
— — after a siesta: calc. carb-v. phos.
— **sleepiness**, with: cham.
— **smoking**, after: alum. bell. ferr-i. gels. petr.
— **speaking**, when: staph.
— **spirituous** liquor, from: *alum.* bell. bov. *con.* coral. petr. stront.
— **standing**, when: bov. bry. cic. grat. lith. plb. staph. thu. valer. verat.
— **stooping**, when: bov. calc. caus. coloc. corn. hell. nat-m. nit-ac. phos. spig. valer. vinc.
— — amel.: verat.
— **sun**, in the: nat-c. nx-v.
— **sweat**, during: chin. samb.
— **turning**, on: am-c.
— **vertigo**, during: acon. æth. agar. arg-n. bar. bov. calc. calc-p. carb-an. caus. cham. clem. creos. dig. ferr. gels. glon. graph. hell. hydr. hydr-ac. ipec. laur. lyc. mill. mosch. mur-ac. nx-m. op. phos. ph-ac. phyt. ran-sc. sabin. secale. seneg. sil. stann. staph. stram. sulph. znc.
— **vexation**, after: nx-v.
— **vomiting**, during: arn. croton. graph.
— **waking**, on: acon. anac. ant-t. arg-n. ars. bar. berb. bry. calc. calc-p. caps. carb-v. cham. chin. clem. cocc-c. con. euphr. glon. hyper. ign. lyc. mag-s. merc. merc-i-fl. phos. *ph-ac.* psor. rhod. rhus. ruta. stann. sulph. tilia.
— **walking**, when: agar. ang. asar.

bell. borax. bry. calc. camph. carb-an. carb-v. cic. cocc-c. coff. con. dros. ferr. k-ca. nat-m. nit-ac. rhus. sep. spong. tarax. thea. thu. viol-tr.
— — in open air: acon. agar. carb-v. caus. coff. k-clc. lyc. nat-m. sep. spig. sulph. tarax.
— — — — — amel.: bry. merc-i-fl. nat-c. par. rhod. sulph.
— **washing** amel.: calc-p. coca. cyc. euphr. phos.
— **wine**, after: alum. bov. con.
— **wrapping** up head, amel.: mag-m.
— — uncovering head, amel.: phos.
— **writing**, when: arg-n. croc. ferr-iod. laur. nat-c.
— **yawning**, amel. when: bry.

**Congestion**, rush of blood, etc.: acet-ac. *acon.* aloe. alum. *ambr.* am-c. am-m. anac. *ant-c. apis.* arg-n. *arn.* asaf. *aur.* bar. *bell. borax.* bov. brom. *bry.* cact. *calc.* camph. cann-i. *cann-s.* canth. carb-an. carb-v. caus. *cham. chin.* cic. *cimic.* cinnb. clem. cocc-c. coff. colch. coloc. con. cop. coral. corn. creos. croc. crotal. croton. cupr. cyc. dig. dulc. elaps. eucal. eugen. eup-per. *ferr.* fluor-ac. gamb. *gels. glon.* gran. graph. *grat. hell.* hura. hydr. hydr-ac. *hyos.* ign. indg. *iod.* jatr. k-bi. *k-bro. k-ca.* k-clc. k-iod. kalm. *lach.* lac-ac. lact. *laur.* lil-t. *lyc.* mag-c. mag-m. *mag-s. mang.* merc. merc-c. merc-i-fl. *mill. mosch.* naja. nat-c. nat-m. *nit-ac.* nitr. nx-m. *nx-v.* ol-an. *op.* pæon. petr. phel. *phos.* ph-ac. pic-ac. plb. psor. puls. ran-b. rhus. sabin. sang. secale. seneg. *sep. sil. spong.* staph. *stram.* strych. *sulph.* sul-ac. *tabac.* tarax. tarent. tellur. thea. thu. urt-ur. valer. *verat.* verat-v. viol-od. znc. zing. ziz.
— — compare with Fullness, Pulsation, etc.
— — **alternating** with congestion to heart: glon.
— — — with icy cold sensation: *calc.*
— — **chronic**, caused by fright or grief: ph-ac.
— — **extending** to, from abdomen: croton.
— — — from chest: *glon.* mill. sulph.
— — as if blood rushed from heart to head: nx-m.
— — at night, a stream from chest to head like a gust of wind, with epistaxis: *mill.*
— — as if blood streamed from below upwards or from within outwards: oxal-ac.
— **forehead**, in: aloe. bad. bell. cimic. cinnb. fluor-ac. glon. lac-ac. mag-s. nat-c. ran-b. spong. stann. viol-od.
— **occiput**: aloe. borax. chel. *glon.* ol-an. pip-m. staph. thu.
— **temple**: chel. glon. sil. zing.
— **vertex**: absin. cann-i. cinnb. phos. ran-b. sil.

**Conditions** of Congestion.
— **morning**: calc. cham. chin-s. glon. lach. lac-ac. lyc. mag-c. mag-s. naja. raph. tellur.
— — on waking: *calc.* lyc.
— — on rising: eugen.
— — worse toward noon, gradually ceasing toward evening, with terrible pain, would press head against wall; fears going mad: stram.
— **afternoon**: am-c. cham. chin-s. lach. nat-m. pæon. ran-b. sil.
— **evening**: calc. caus. chin-s. fluor-ac. indg. mag-m. mill. nat-c. nx-v. phos. puls. rhus. trom.
— **night**: am-c. anac. carb-v. cyc. mill. *psor.* puls. sil. sulph.
— **abdomen**, during pain in: coloc. sang.
— **alcoholic** liquors aggr.: *calc. glon.* lach.
— **anxiety**, with: *acon.* cyc.
— **air**, in open: lil-t. nat-c. ran-b. *sulph.*
— — — amel.: camph. caus. hell. mag-m. mosch.
— **bandage**, amel. by a tight: arg-n.
— **bed**, when in: anac. lyc. mill. sulph.
— **chest**, during shocks in: tabac.
— **coffee**, from: am-c. mill. rumx.
— **constipation**, during: aster. crotal. nx-v.
— **convulsions**, before: glon.
— — during: canth. crotal.
— **coryza**, with sensation as of: carb-v.
— **dentition**, during: verat-v.
— **diarrhœa**, during: rhus.
— **dinner**, after: cyc. nx-m.
— **eating**, before: uran-nit.
— — after: cinnb. cop. cyc. glon. nx-m. petr.
— — high living, from: verat-v.
— **epistaxis**, with: bell. carb-vh. lac. lil-t.

— — feels as if were coming on: ign. lac-ac.
— **excitement**, during: asaf.
— — after a pleasant surprise: coff.
— **exercise**, on taking: sulph.
— **eyes**, with congestion of: canth. lac-ac. nit-ac. seneg.
— — — dim vision: alum. cyc. grat. hydr-ac. lac-ac. nit-ac.
— **face**, with congestion of: cop. coral. corn. merc-c. mill. ziz.
— — **heat** of with: acon. asaf. canth. chin-s. coff. cop. ferr. hell. kalm. mang. phos. rhus. sil. *sulph.* valer.
— — **redness** of with: acon. bell. canth. coff. glon. phos. sil. sol-n.
— **fright** or grief, from: ph-ac.
— **hæmorrhoids**, with: lach.
— **head**, on raising: lyc.
— — on bending back: bell.
— — on shaking: nit-ac. nx-v.
— **heart**, at every throb of: cimic. glon.
— **lifting**, after: nat-c.
— **lochia**, from suppressed: acon. bell. bry. cimic.
— **lying**, when: cyc. hydrph. mang. naja.
— — on temple: mur-ac. (r).
— — amel.: nat-c.
— **menses**, before: *acon. apis. bell.* bry. cupr. gels. *glon.* hepar. hipp. hyper. iod. lyc. merc. trill.
— — **during**: acon. apis. *bell. bry.* cact. *calc.* caus. cham. *chin.* cinnb. con. elaps. gels. *glon.* iod. mag-c. mag-m. manc. merc. mosch. nat-m. *nx-v.* phos. sang. sulph. verat. verat-v.
— — **after**: chin. nat-m. sulph. thu.
— — **suppressed**, from: *acon. apis.* arn. *bell. bry. calc.* cham. *chin. cimic.* ferr. gels. graph. lach. merc. op. stram. sulph.
— **mental** exertion, from: agar. aur. *calc.* cham. psor.
— **motion**, from: glon. grat. k-clc. mang. nx-v. petr. sulph.
— **nausea**, with: glon. pæon.
— **nose**, on blowing: nit-ac.
— — see, also, Epistaxis.
— **pains**, when, suddenly cease: cimic.
— **parturition**, in: aur.
— **pressure**, amel.: camph. glon.
— **respiration**, with difficult: acon. œna.
— **riding**, from: grat. sulph.
— **rising**, on: eugen. mag-s. nat-c. sil. sulph.
— — amel.: aur. mill.
— **room**, on entering: ol-an.
— — in a hot: carb-v. cocc-c. sulph.
— — sitting in amel.: sulph.
— **sitting**, when: lac-ac. mag-c. mang. nat-c. phos. thu.
— — must sit up: aloe.
— **sleep**, during: glon. sil.
— — after amel.: grat.
— **smoking**, from: mag-c.
— **speaking**, when: coff. sulph.
— — when spoken to: ign.
— **standing**, from: k-ca. mang.
— **stepping** heavily, from: bar.
— **stool**, during: *bry.* sulph.
— — after: lach.
— **stooping**, when: acon. aur. bell. calc-p. canth. coral. elaps. lach. lyc. mill. myric. nat-c. nit-ac. rhus. seneg. sep. sulph. tellur. verat.
— **sun**, from exposure to: acon. cact. glon.
— **suppressed** discharges or suddenly ceasing pains: cimic.
— **sweat**, during the: thu.
— **vertigo**, with: *acon.* alum. arn. *bell.* bry. cact. calc-p. chin. con. eugen. glon. grat. k-clc. lach. merc. mosch. myric. *nx-v.* op. rhus. sabad. sil. sulph. urt-ur.
— **waking**, on: am-c. bell. carb-v.
— **walking**, when: caus. lach. mang. ran-b.
— — in open air: caus. ran-b.
— — — — amel.: cham.
— **wet**, from getting the feet: dulc.
— **wine**, after: sil.
— **working**, when: tellur.
— **writing**, when: cann-s.
**Constrictive.** See Compressive.
**Continuous** pain, increases periodically: cupr.
**Contusive.** See Bruised.
**Cord**, sensation as of a, across forehead: merc-i-r. nat-c.
— see also under Compressive.
**Cracking**, crackling sensation in: acon. ars. calc. carb-v. cham. con. dig. kalm. puls. sep. spig.
— — as if something broke: sep.
— **forehead**, in: acon. spig.
— **side** of: acon. arn. calc. cham. coff. hepar.
— **occiput**, in: calc. carb-v.
— **vertex**, in: *coff.* con.
— **evening**, aggr. in: acon.
— **motion**, aggr.: acon.

— **nose**, after blowing: hepar.
— **shivering**, with: kalm.
— **siesta**, during: dig.
— **sitting**, when: carb-v. coff.
— — amel. when: acon.
— **turning** the head, when: sep.
**Cramping** pain: *acon.* *ambr.* am-m. anac. ang. *ant-t.* ars. asaf. calc. carb-v. cina. colch. *coloc.* croc. eugen. gels. *ign.* k-ca. mag-m. marum. mezer. nit-ac. nx-v. olnd. petr. *ph-ac.* *plat.* psor. ran-sc. rheum. sep. squil. stann. thu. verb. znc.
— — compare with Compressive.
— — **extending** to malar bone: bell.
— **forehead**: æth. bell. croc. ign. *plat.*
— — above root of nose: arn. bell.
— — — — as if would lose senses: *acon.* ign.
— **occiput**: am-m. dios.
— — in small spot: am-m.
— **side** of: bell. (r), phos. sars. thu. (l).
— — cold, crampy, left: phos.
— **temple**: agar. (l), calc. (l), cina. indg. (l), k-ca. (l), petr. plat. sil. (l), verb. znc.
— — extending into teeth: nat-m. (r).
— — with tickling: cann-s.
— early morning, after rising: mag-c.
— from rubbing forehead, amel.: thu.
— from suppressed catarrh: *acon.*
— study and exertion from, after ague: gels.
— vexation, after: mag-c.
**Crazed** sensation, in: carb-v. lil-t.
— with pain in right iliac region: lil-t.
— pain so severe believes will go crazy or lose senses: *acon.* ambr. *calc.* ign.
— — feels crazed with headache, when studying: indm.
— Compare with Wild.
**Crawling**, creeping sensation: arg. arn. calc-p. colch. cupr. hyos. plat. puls. *rhus.* sulph.
— in brain: alum. apis. laur. puls.
— forehead: colch.
**Cry** out, pains compel one to: anac. bov. *coloc.* cupr. mag-m. *sep.* sil. stann. stram.
**Cutting**, darting, stabbing, etc.: agar. alum. ambr. arg. arn. aur. *bell.* bism. calc. camph. canth. caps. carb-v. caus. chin. cina. con. dros. ferr. hepar. k-bi. k-clc. lach. lyc. mag-c. mosch. nat-m. nit-ac. puls. staph. tilia. verat.
— — compare with Lancinating, Shooting, etc.
— — as if brain were cut to pieces, on stooping: nicc.
— — as if split by a wedge, body icy cold, thirst: lachn.
— — as with a knife: alum. arn. *bell.* k-bi. lach. mag-c. mag-s. nat-m.
— — — followed by sensation of coldness: arn.
— **forehead**, in: agar. *bell.* bism. calc. camph. cocc-c. dios. dros. nat-m. seneg. sep. ziz.
— — as with a knife: mang. nat-m. sabin. tereb.
— — **extending** to occiput, whereupon all previous pain suddenly ceases: bell.
— — across forehead, followed by stitches in epigastrium: æsc-h.
— — above right orbit, extending to occiput: bism.
— — momentary darts causing one to close eyes: podo.
— **occiput**: *bell.* calc. caps. chin. tong.
— — **extending** to orbits, aggr. by motion and stooping: chin.
— — as with a knife: con. nat-m.
— **side** of: arg-n. aur. *bell.* (r), cic. k-bi. nat-m. nat-p. rumx. (l).
— — as of a knife drawn transversely through: *arn.*
— **temple**, in: alum. apoc. (r), arg-n. *bell.* canth. chin. cimic. cocc-c. *coloc.* (l), croc. cyc. dios. (l), glon. graph. hydr. iris. lac-can. lach. mag-c. nat-p. phos. ph-ac. ptel. (r), rhus. stram. (r), stront. verb.
— — **extending** to jaw: glon.
— — — to eyes: berb.
— — as with a knife: cyc. ferr. lach. stram.
— — — — from temple to temple: *bell.*
— — sharp pains by spells in both temples: bapt.
— **vertex**: bell. con. *lach.* senec. verat.
**Conditions** of Cutting.
— **afternoon**: nat-p. ptel.
— **evening**: *bell.*
— **air**, open aggr.: nat-m.
— **cold** applications, amel.: tilia.
— **coughing**, when: ziz.
— **exertion**, on: ambr. tilia.
— **eyes**, closing amel.: tilia.
— — moving aggr.: dros.
— **motion**. aggr.: chin. tilia.

— **nose**, after blowing: sep.
— **rest**, aggr.: capr.
— — amel.: tilia.
— **sleeping**, amel.: stram.
— **standing**, when: agar.
— **stooping**, on: chin. dros. nicc.
— **supporting** head on hands, amel.: dros. hydr.
— **thinking** of it, amel.: cic.
— **walking**, aggr.: calc.
— — amel.: caps. hepar.
**Darting**. See Cutting, and compare with Lancinating, Shooting, etc.
**Digging**. See Boring.
**Dilate**, expand, etc., seems to: bell. cann-i. carb-ac. dulc. euph. sol-n. stront.
— on shaking head, aggr.: carbol-ac.
— Compare Swollen.
**Distention**. See Swollen, and Compare Fullness, Pressure, etc.
**Distorted**, feels as if: nat-m.
**Dragging** sensation in: ant-t. calc. canth. crotal. gels. laur. merl. nat-m. rhus.
— extending to shoulders: gels.
— sitting and leaning against high pillow amel.: gels.
**Drawing**: *acon.* æth. agar. ailan. ang. alum. ambr. am-c. ant-t. apis. arg. arg-n. ars. asar. aur. bar. bell. berb. bism. borax. bov. bry. *calc.* camph. canth. caps. carb-an. *carb-v. cham.* chin. cimx. cimic. cina. coff. coloc. *con.* creos. croc. cupr. cyc. diad. dulc. eugen. eupi. ferr. glon. gran. graph. guai. hell. hipp. hydr. ipec. k-ca. k-iod. lach. lil-t. lyc. *mag-c.* mang. men. merc. mezer. *mosch.* nat-c. *nat-m.* nit-ac. nx-v. ol-an. petr. plat. puls. ran-sc. rheum. *rhod.* rhus. ruta. sabad. sabin. seneg. sep. sil. squil. stann. staph. stront. *sulph.* sul-ac. thu. tilia. tong. valer. verat. *znc.* zing.
— — **behind** forward, from: carb-v.
— — **extending** to face; ant-t. diad. graph. seneg.
— — — to nose: ant-t.
— — — to occiput: glon.
— — — to spine: *mosch.* nitr. thu.
— — — to temples: asar.
— — **here and there**: ambr. ipec. mosch. nx-v. tong.
— — drawing hot pain, head and eyes, sneezing relieves: lil-t.
— — **periosteum**, as if in: merc-c.
— — **preceded** by drawing in armpit: petr.
— — **round** the head: bov. carb-v.
— — in stripes, as if in: arg-n.
— **forehead**: acon. agar. all-c. am-c. anac. ang. ant-cr. ant-t. arg-n. ars. asaf. (1), asar. aur-m. bad. bar. (1), bell. borax. bry. cann-i. cann-s. canth. caps. (r), carb-v. cast. caus. chel. chin. cic. cina. (1), clem. (1), colch. (1), coloc. (1), con. *croc.* cyc. (1), dulc. (1), eupi. ferr. gins. graph. guai. hell. hipp. *k-ca.* lact. laur. lil-t. lyc. mag-c. mang. men. (r), merc. mezer. mosch. nat-m. nat-s. nit-ac. (r), nitr. nx-v. petr. phos. puls. ran-b. rheum. *rhod.* (1), ruta. (r), sabad. sabin. (r), selen. seneg. sep. sil. squil. stann. (r), staph. stront. sulph. tarax. valer. verb. (1), viol-od. (1), *znc.* zing.
— — **alternating** with pain in wrist: sulph.
— — **extending** to eyes: agar. cann-i. lil-t.
— — — to lower jaw: nat-m.
— — — to neck: *mosch.* nitr. viol-tr.
— — — to nose: glon. nx-v.
— — — to occiput: *sep.*
— — **flying**: chel.
— — **eyes**, above: *agar.* asaf. bry. calc-ac. *cann-i.* carb-an. colch. con. *ign.* (r), nat-m. (1), nit-ac. seneg. sulph. thu. (1), znc.
— — — eyes feel as if projecting, with sensation as if a thread were tightly drawn through eyeball and backward into middle of brain, sight weak: par.
— — **nose**, above root of: *acon.* agar. asar. *carb-v.* k-clc. nat-m. rheum.
— — **worm**, as if a crept through: sulph.
— **occiput**: agar. ambr. anac. ant-t. arg. arg-n. arn. asaf. bell. BRY. cact. calad. calc. calc-ac. calc-p. cann-s. carb-v. caus. chel. cocc. cocc-c. coloc. corn. cyc. dros. gels. gins. glon. graph. guai. k-bi. laur. mag-c. mang. men. merc. mill. mosch. mur-ac. nat-c. nat-s. nitr. *nx-v.* phos. ph-ac. plat. plect. puls. ran-b. raph. rhod. rhus. sabin. selen. sep. spig. squil. staph. sulph. valer. *znc.*
— — **extending** to ears: bar. (r), cann-s.
— — — to forehead, amel. by pressure and walking: chin.
— — — to neck. before going to sleep: *bry.*

— — — to nape of: merc. nat-c. plect. sulph.

— — — to nose: corn.

— — — to upper cervical region and shoulders, better resting head high on pillow, with eyes half closed; sleepy: gels.

— — **wandering**: mezer.

— — **upward** from nape: ambr. carb-v. ferr.

— **side** of: *acon.* alum. (r), anac. (l), ang. (r), ant-t. (l), apis. (l), *arg.* (l), arg-n. (r), arn. (l), asaf. (r), bar. (l), bar-ac. bell. (r), brom. (l), calc. (r), camph. (r), canth. (l), caps. carb-v. *cham.* chin. cimx. cina. clem. cocc. (r), colch. (l), coloc. dig. dros. fluor-ac. (r), gran. hell. indg. iod. (l), k-ca. (l), lach. (r), lyc. (r), men. nat-s. nit-ac. (l), nx-v. phos. (r), ph-ac. (r), plat. rhus. (l), sars. (l), sep. (l), spong. (r), sul-ac. (r), thu. (r), valer. (r).

— — **extending** to clavicle: indm.

— — — to face: cup-ac.

— — — to neck: *chel.* (r), cupr-ac. lyc.

— — — to orbits: crotal. (l).

— — — to teeth: crotal. iod.

— — in **spot**: phos.

— — increases gradually and ceases suddenly, feeling as if a nerve had been torn: arg. (l).

— **temple**: acon. agar. ang. ant-c. (l), ant-t. arg. asar. bell. (r), bry. calc. cann-i. casc. (r), caus. (r), chel. chin-s. cina. coff. (r), colch. (l), *coloc.* (r), con. croc. (r), cupr. (l), cyc. (l), dulc. (l), eupi. guai. hepar. hipp. indg. (l), k-bi. laur. lyc. mang. merc. (r), mosch. nit-ac. *nx-v.* ol-an. olnd. petr. phos. ph-ac. phyt. plat. (l), ran-b. raph. rhod. rhus. ruta. sabad. (r), sabin. sars. (r), seneg. spig. (l), squil. (r), stann. stront. sulph. sul-ac. tabac. *tarax.* (l), thu. (l), tilia. (l), znc. (l), zing. (r).

— — **extending** to eye: aloe. (l).

— — — to face: ant-t. (r), arg. *bry.* seneg.

— — — across forehead: chin-s. lach. lact. lyc. sabin.

— — — to vertex: aur-m. cyc. (l).

— — **spots**, in: sul-ac. (l).

— **vertex**: anac. ant-t. arg. ars. bov. *calc.* caus. chel. cinnb. crotal. dulc. grat. hell. indg. iod. k-ca. led. nx-m. nx-v. ol-an. phos. ph-ac. ran-b. ran-sc. ruta. sars. spig. spong. stann. tilia. znc.

— — **extending** to eyes: nx-m.

— — — to forehead: led.

— — — to nose, while eating: dulc.

— — — to neck, aggr. by walking: chel.

— — — to temple: chel.

**Conditions** of Drawing.
— **morning**: ang. dros. hell. k-bi. mag-c. mezer. petr. sulph. znc.
— — on rising: nat-m.
— — wakens, passes off on rising: am-c.
— **noon**, amel. of: BRY.
— **afternoon**: agar. ant-t. dulc. gins. verat-v. zing.
— **evening**: all-c. aloe. ang. bov. cast. crotal. graph. hipp. nitr. ol-an. phos. ran-b. stront. valer. znc.
— — amel. in: coloc.
— **night**: *k-ca.* nat-m. phos. rhus.
— **air**, in open: *con.* grat. mang. plect.
— — — amel.: asar. hell. olnd.
— **bed**, in: agar. hell. hipp.
— **breath**, aggr. when holding: agar.
— **chewing**, when: sulph.
— — hence amel. after eating: sulph.
— **chilliness**, during: eupi.
— **church**, when in: znc.
— **cold** applications, amel.: tilia.
— **dinner**, after: bell. nat-c. phos.
— **draught** of air, from: tilia. valer.
— **eating**, during: dulc.
— — after: ant-t. bell. cina. mill. nat-c. phos.
— — — amel.: con. sulph.
— **eyes**, on closing: sabad.
— — amel.: tilia.
— **head**, on moving: cact. staph.
— — resting high on pillows, amel.: gels.
— **house**, on entering, amel.: mang. plect.
— **lying** down, amel.: asar.
— **menses**, during: berb. mag-c. sang.
— **mental** exertion, from: calc-ac. cina. coff. gins.
— **motion** aggr.: arg-n. bism. tabac. tilia.
— **nausea**, during: croc.
— **pressure** aggr.: cina.
— — amel.: chin.
— **rest**, when at: arg. bell. eupi.
— — amel.: arg-n. tilia.
— **rising**, when: bry. coloc. nat-m.
— **sitting**, when: arg. chin. men. squil.
— **sneezing**, frequent amel.: lil-t.

— **standing**, while: agar. mag-c.
— — amel.: *tarax.*
— **stooping** low, on: *ign.*
— **stretching**, aggr. by: agar.
— **swallowing**, on: mag-c.
— **touch**, on: con.
— **waking**, when: agar.
— **walking**, when: chel. coloc.
— — — amel.: chin.
**Drawn**, backward, the head: acon. acet-ac. bell. camph. cann-i. cedr. cham. chin. cic. cina. creos. cup-ac. eup-per. ipec. mag-c. mur-ac. nat-c. nit-ac. nx-v. op. phel. stram. tong. viol-tr.
— — compare with Falling.
— — in convulsions: tabac.
— — during menses: znc.
— **forwards**: hydr-ac. merc. par. plb. sang.
— **sideways**: *bar.* bell. (1), camph. (1), caul. (1), cup-ac. *lach.* (1), merc. (1), plb. (1), puls. sabad. (r), sil. taxus. (1).
— — first right then left: ang.
— — first left then right: stram.
— — upon shoulders: hydr-ac.
**Dropsy** of brain: acon. am-c. apis. apoc. *aur.* bell. calc-p. hell. indg. lach. mag-m. ph-ac. plat. samb. stram.
— — with sweat: merc.
**Dull** pain: *agar.* ailan. all-c. alum. anac. ant-c. arg. arum-t. bapt. bar. bism. bov. calc. camph. cann-i. canth. carb-an. carb-v. caus. cham. chel. chin. cina. con. croc. crotal. cupr. eup-pur. ferr. glon. graph. ham. hell. hepar. hyos. ign. *lach.* lachn. lact. laur. led. lyc. mag-m. mang. marum. men. meph. merc. mosch. nat-c. nat-m. nit-ac. petr. ph-ac. plat. puls. ran-sc. rheum. rhod. sabad. sars. secale. seneg. sep. sil. spig. spong. squil. sulph. sul-ac. tereb. thu. verat. verb. viol-od. viol-tr.
— — compare Aching.
— **forehead**: agar. ant-c. ant-t. asar. bapt. calc. camph. cann-i. cocc. coff. coloc. dulc. (1), euph. hell. ign. laur. lept. nat-m. ph-ac. plat. plb. puls. rheum. sabad. sars. verat. znc.
— — **eye**, over: apis. cann-i. znc.
— — in right frontal protuberance, then in left: acet-ac.
— **occiput**: æsc-h. alum. ambr. asar. bry. calc. chin. cic. cyc. fluor-ac. gels. nat-c. ran-sc. rhod. samb. *secale.* stront. thu.
— **side** of: canth. croc. dros. laur. spong. znc.
— **temple**: agar. chin. laur. ph-ac. stront. verat.
— — from temple to temple: lobel.
— **vertex**: agn. ant-c. cimic. gels. mezer.
— **morning**: agar.
— **afternoon**: bapt.
— **move** head, must and close eyes: agar.
— **pressure**, amel.: cimic.
**Dullness**, cloudiness, stupid feeling, etc.: abrot. acet-ac. æsc-h. æth. agar. agn. ailan. alco. all-c. alum. am-c. ant-t. apis. apoc. arg. arg-n. ars. arum-t. asaf. asar. bad. bapt. bar. bell. berb. bism. bov. bry. *calc. calc-p.* cann-i. cann-s. caps. carb-v. cast. caul. caus. cedr. cham. chel. chin-s. cic. cimx. cimic. cinnb. clem. cob. coca. cocc. cocc-c. colch. con. creos. croc. crotal. croton. cub. cup-ac. dig. dios. dros. dulc. elat. eucal. ferr. ferr-p. fluor-ac. gamb. *gels.* glon. graph. grat. guai. hæmat. hell. hura. hydr. hyper. iris. jatr. k-bi. k-ca. k-iod. kalm. lach. lachn. lac-ac. lact. laur. led. lil-t. lyc. mag-m. mag-s. mang. marum. men. merc. merc-c. merc-i-fl. merc-i-r. mezer. mill. mosch. myric. naja. nat-c. nat-m. nat-p. nat-s. nicc. nit-ac. nitr. nx-m. nx-v. olnd. ol-an. op. osm. oxal-ac. pæon. pallad. par. phel. phos. ph-ac. phys. phyt. pic-ac. plan. plat. plect. plb. podo. prun. psor. ptel. puls. ran-sc. ratan. rhod. rhus. rhus-v. ruta. sabad. sang. sars. secale. selen. seneg. sep. serp. sil. sol-n. spig. spong. stann. staph. stram. stront. sulph. sul-ac. tabac. tarax. tellur. tereb. thu. tilia. urt-ur. ustil. valer. verat. verat-v. verb. viol-tr. vip. xanth. znc.
— — Compare with Confusion, Stupefaction, etc.
— — **blood**, as from too much: calc. glon. phos. sil. *sulph.*
— — **chronic**: calc.
— — **coryza**, as from: ars. berb. k-ca. lyc. *merc-i-r.* nicc. op. phos. sep.
— — **dizzy**, dullness: agar. iris. mosch. par. sabad. tilia.
— — **drinking**, as after: ang. bry. cimic. cocc. creos. dulc. glon. mag-m. *mezer.* nicc. nitr. op. ph-ac. rheum. rhod. *rhus.* sabad.

— — **gloomy**: ang. arg. calad. clem. dig. men. merc. mezer. nat-m. nitr. *nx-v.* op. ph-ac. puls. rheum. samb. thu. valer.
— — **fatigue**, as from: nat-m.
— — **intoxicated**, as if: æth. caus. mezer. ratan. rhus. samb. thu.
— — **muddled**, as if: agar. anac. ant-c. ars. asaf. bry. coral. euphr. ferr. men. nx-v. puls. rhus. secale. seneg. staph. tabac. thu. verb. viol-od. viol-tr.
— — **nailed** up, as if: *acon. æth.* mag-s. plat.
— — **opium**, as from: cann-i.
— — **painful**: ang. arn. *asaf.* calc. caus. diad. dros. glon. grat. hell. merc. nat-c. *nat-m.* nx-m. plat. secale. spig. tilia. viol-od.
— — **sleep**, as from too much: nat-c.
— — — as from loss of: ambr. ars. bry. chin. colch. ferr. k-ca. mezer. nicc. nx-v. op. phos. ph-ac. rhod. ruta. sulph. znc.
— — **stunning**: ang. ant-t. arg. asar. aur. *cocc.* croc. dulc. k-ca. mag-m. mag-s. mezer. par. rheum. sul-ac. verb.
— — **sudden**: bry. sul-ac.
— **forehead**, in: æsc-h. agar. ailan. ant-c. arg-n. arum-t. bell. calc. cann-i. canth. carb-an. cham. cimic. cinnb. *cocc.* coloc. croc. cund. euphr. ferr. fluor-ac. gels. glon. gymn. ham. hell. hepar. hipp. hydr-ac. ign. jac. lac-can. lachn. mang. merc-c. mur-ac. naja. nat-m. *nx-m. op.* oxal-ac. pallad. par. phos. phyt. pip-m. psor. (1), ratan. rhod. rumx. sabin. serp. sil. sol-n. squil. sulph. sul-ac. tabac. tilia. ustil. vinc. *znc.*
— — **board**, before, like a: acon. calc. cocc. dulc. olnd. op. plat. plb.
— — as if something worked around in: merc.
— — mounting like a vapor from occiput: æth.

**Conditions** of Dullness.

— **morning**: agar. alum. ars. arum-t. *calc.* carb-an. cob. coloc. hyper. ign. indg. mag-m. mag-s. nat-c. nat-m. nat-p. nicc. nx-v. ol-an. oxal-ac. petr. podo. *rhus.* sulph. sul-ac. thu. tilia. ustil.
— — on waking: arg-n. chin. croton. ferr. myric. nat-m. plect. rhod. znc.
— — on rising: anac. calc. cocc-c. hura. mag-m. plb.
— **afternoon**: agar. almn. chel. lac-ac. laur. mag-s. merc-i-r. nat-m. sol-n. verat-v.
— **evening**: ars. cedr. croc. dulc. elat. grat. k-ca. mag-s. mezer. mill. nat-m. oxal-ac. pallad. *nx-m.* phos. phys. selen. sep. spig. stann. sulph. thu. znc.
— **night**: arg-n. fluor-ac. pic-ac. *sulph.* tilia.
— **abdomen**, during colic at night: *sulph.*
— — during pressure in: graph. sep.
— **air**, in open: rhod. spig.
— — — amel. in: dulc. phos. phyt. ratan.
— — — exercise in, amel.: rhod.
— **ascending**, on: *sulph.*
— **breakfast**, before: fluor-ac.
— — amel. after: bov.
— **catarrh**, during: graph.
— **chilliness**, with: aloe. hell.
— **coffee**, after: arg-n. mill.
— **coition** (or emissions), after excessive: caus. mezer. ph-ac.
— **cold**, after: olnd.
— — washing amel.: phos.
— **conversation**, from: sil.
— **coryza**, during: nat-m.
— **covered**, in bed: amel.: nat-c.
— **diarrhœa**, after: nx-v.
— **dinner**, after: arg-n. mag-m. plan. sabad. thu. tilia.
— **draught** of air, from: *calc-p.*
— **drinking**, after: con.
— **eating**, after: arg-n. calc. cocc. lyc. mag-m. men. mill. nat-m. phos. plan. sabad. thu. tilia. znc.
— — amel.: bov. mezer.
— **eructations**, amel.: sang.
— **exertion**, in open air, amel.: rhod.
— **eyes**, on turning: rhus.
— **fever**, during: thu.
— **hat**, worse from pressure of: calc-p.
— **head**, covering the, on: stront.
— — resting on table, amel.: rhod.
— — shaking, aggr.: glon.
— **house**, aggr. in: phos.
— **menses**, after: graph. *nat-m.*
— **mental** exertion, from: cocc. mag-c. nat-m. oxal-ac. phos. thu.
— **micturition**, free, amel.: tereb.
— **motion**, from: nx-v. ol-an. tabac.
— — amel.: ferr-p.
— **nausea**, during: fluor-ac.

— **periodic**: staph.
— **rising**, after: laur. nat-s. olnd. rhod.
— **room**, in warm: bell. croc. merc-i-fl. phos.
— **siesta**, after: calc.
— **sitting**, when: phos. phyt. puls. rhus. sars. verat.
— **sleep**, after loss of: ph-ac.
— **smoking**, from: gels. thu.
— **sneezing** amel.: lyc.
— **standing**, while: staph.
— **stool**, during: dios. oxal-ac. phos.
— — amel. after: mag-s.
— **stooping**, on: nat-m.
— **vertigo**, during: acon. æth. am-c. ars. borax. bov. camph. canth. carb-an. caus. cham. clem. cocc. coff. coloc. croc. lach. laur. mag-c. mag-m. nitr. *nx-v.* op. phos. secale. sep. sulph.
— **vomiting**, amel.: tabac.
— **waking**, on: acon. ars. caps. cham. con. sep. sulph. znc.
— **walking**, from: coloc. grat. nat-c. nat-m. sep.
— — amel.: agar. ferr-p. sulph.
— — in open air amel.: merc-i-r. rhod.
— **wine**, from least: *alum.* coloc. *con.* k-clc. mill. oxal-ac. *znc.*

**Empty**, hollow sensation: am-c. anac. ant-c. arg. arn. ars. asaf. bell. berb. bov. cact. calc. camph. caps. carb-v. caus. chin-s. cina. clem. *cocc.* coral. cupr. cyc. dulc. euphr. ferr. glon. gran. graph. hipp. hyos. ign. jabor. manc. mang. myric. naja. nat-c. nat-m. nat-p. nx-v. oxal-ac. phos. pic-ac. *puls.* secale. *seneg.* sep. stram. sulph. zing.
— — aching of brain, with: arg.
— — as from catarrh: ars.
— — as after intoxication: acon. agar. ambr. spig.
— — head feels like a lantern: ars. puls.
— **forehead**, in: alum. caus. spig. sulph. sul-ac.
— — as if between forehead and brain: caus.
— **occiput**: mang. nat-c. sep. staph. *sulph.*
— — while brain in front seems too large: hell.
— **temples**: cyc.

**Conditions** of Empty sensation.
— **morning**: anac. bov. chin-s. euphr. sulph. verat.
— **afternoon**: nx-m.
— **night**, aggr. lying on occiput; amel. by pressure of hand: sep.
— **air**, in open, aggr.: cocc. sulph.
— **bed**, amel. on getting warm in: cocc.
— **coryza**, during: hell.
— **eating**, after: cocc. graph. men.
— **house**, amel. in: sulph.
— **pressure** of hand, amel.: mang. sep.
— **riding**, amel.: euphr.
— **sitting**, when: spig.
— **sleep**, after restless: hipp.
— **talking**, when: spig. sulph.
— **vertigo**, during: phos.

**Enlarged**, seems: agar. ant-c. ant-t. apis. *arg-n.* ars-i. bapt. *bell.* berb. *bov.* caj. caps. *cimic.* cob. coll. com. daph. dulc. *gels.* gins. *glon.* hyper. indg. k-iod. lach. lachn. lact. laur. lith. *mang.* meph. merc. nat-c. nx-m. phel. ran-b. ran-sc. rhus. sil. spig. tarax. therid. tilia. tong. zing.
— — compare with Swollen.
— **elongated**, feels: hyper.
— **extended** upward, the vertex seems: lachn.
— sensation as if widened: aloe.
— and as if split open by a wedge from the outside; body icy cold, skin moist and sticky; cannot get warm even under a feather bed, face yellow; whines with the pain; head burns like fire, with thirst: lachn.
— **bandaging**, amel.: arg-n.
— **fever**, with intermittent: *cimic.*
— **headache**, with: gels.
— **lying**, when; rising aggr.: rhus-r.
— — amel.: dulc.
— **menses**, during: arg-n. *glon.*
— **pregnancy**, during: arg-n.
— **pulling** on boots, aggr.: coll.
— **stool**, during: cob.
— **walking** in open air, after: ant-t.
— **weather**, aggr. in cold, damp: dulc.

**Expanded**. See Enlarged, Swollen, etc.

**Falling** backward of head: æth. *agar.* camph. chin. colch. dios. glon. k-ca. *led.* œna. op. phel. samb. *spig.* tarent.
— — see also under Heaviness.
— — when sitting: chin. œna. op.
— — during vertigo: *spig.*
— — when walking: *chin.* phel.

— **forward**, feels as if head would: agn. calc. clem. cupr. elaps. gels. glon. hipp. hydr-ac. hyos. *ign.* k-ca. laur. lyc. op. par. phos. phys. pic-ac. plb. *puls.* ran-b. sars. sulph.
— — on rising: hipp.
— — when looking at anything, sinking of head forward: cic.
— — **sitting**, when: nx-m. œna. staph.
— — stooping, when: puls.
— — walking, when: hipp.
— — wrinkling of forehead and open air, amel.: phos.
— **forward**, sensation of, in brain: *alum.* am-c. bar. berb. bry. carb-an. cham. coff. creos. *dig.* grat. hipp. k-ca. laur. mag-s. nx-v. *rhus.* sabad. sul-ac.
— — — — — pain, as if fell forward and came up again: sul-ac.
— — — — — on stooping: *carb-an.* coff. laur. mag-s. nat-m. nat-s. rhus.
— — — — — raising head, amel.: alum.
— **hither** and thither: bell. phel.
— **out**, sensation as if everything would, at forehead: *acon.* all-c. bar. bell. brom. bry. canth. carb-an. caus. chel. colch. coloc. creos. hell. hepar. k-ca. mag-m. mag-s. mezer. nx-v. phos. plat. puls. ratan. rhod. sabad. sep. spig. spong. stann. staph. stront. tabac. thu. verb.
— — see also under Pressure.
— — coughing, when: hepar.
— — stool, when at: ratan.
— — stooping, on: hell. mag-s.
— **to pieces**, sensation as if head would fall, when stooping: glon.
— **sideways** of head: ang. arn. ars. cann-s. cina. dios. eup-per. ferr. fluor-ac. hyos. k-iod. nx-m. op. prun. stram. sulph. tarax.
— — child leans head, all time: cina.
— — to right side: ferr.
— — to left side: nx-m.
— — on waking: sulph.
— — when walking: dios. ferr.
— **side** to side, in brain: nicc. sul-ac.
— — to side, which stoops: am-c.
— — to left temple, on stooping: nat-s.
**Flapping** sensation in brain: *ars.* rhus.
**Flattened**, sensation in forehead: coral.
— as if pressed flat: verat.
**Fluctuating** in: aphis. bell. cimic. coff. glon. hepar. hyos. mag-m. par.
— compare Undulating.
**Fly** off. See under Bursting, Come off, etc.
**Flying**, fleeting pains. See Stitches, Wandering, etc.
**Foreign** body in, pain as from a: fluor-ac. rhod.
— — in right half of brain: con.
**Fullness**: abrot. ACON. æsc-h. agar. ailan. all-c. am-c. *am-m.* ang. apis. apoc. arg-n. arn. arum-t. asaf. bapt. *bell.* berb. *borax.* bov. *bry.* calc. calc-p. cann-i. caps. carb-an. carb-v. *carlsbad.* cast. cham. chin. chin-s. chr-ac. cimic. cinnb. clem. cob. cocc-c. coff. con. corn. creos. crotal. croton. cupr. cyc. daph. dig. dios. elaps. eucal. ferr. fluor-ac. *gels.* gent. gins. GLON. graph. grat. guai. gymn. ham. hell. hydr. hyos. hyper. ign. iris. jac. k-bi. k-ca. k-iod. kalm. lach. lac-ac. lact. laur. lil-t. lyc. melilo. meph. *merc.* merc-i-r. merc-s. mill. nat-c. nat-m. nat-p. *nicc.* nit-ac. nx-m. nx-v. op. osm. pæon. petr. phel. phos. phys. phyt. pic-ac. plan. psor. ran-b. *ran-sc.* raph. *rhus.* rumx. samb. sang. senec. sep. sil. sol-n. spong. stram. *sulph.* sul-ac. tabac. tanac. tellur. *tereb.* thu. tilia. urt-ur. valer. verat-v. *xanth.* ziz.
— — compare with Congestion, Heat, Heaviness, Pressure, etc.
— — as if would burst: am-c. cann-i. daph. ipec. lil-t. merc. nit-ac.
— — as if too full of blood: lac-ac. sulph.
— — causing head to hang down: clem. phel.
— — as if stuffed full: glon. indm.
— — chilliness, thick speech, double vision, with: *gels.*
— **forehead**: acon. agar. am-m. ang. apoc. arg-n. bapt. bell. berb. borax. bry. calc. cann-i. carb-an. chr-ac. cinnb. clem. coca. euph. eupi. gels. *glon.* gymn. ham. hell. helon. hydr. hyos. indg. indm. laur. lil-t. mag-s. naja. nat-p. nicc. oxal-ac. pallad. phys. phyt. podo. ran-sc. rhus. rumx. sang. sep. sulph. sul-ac. thea. tilia.
— — as if there was not room enough, a forcing out, amel. washing and eating: psor.
— — **eyes**, over: hydr. lil-t. nat-p. oxal-ac.
— — — with vertigo: podo.

— — **nose**, over, evening: naja.
— **occiput**: agar. apis. bapt. caj. cann-i. cinnb. con. creos. glon. helon. osm. sulph. therid.
— **temple**: alco. apis. bell. cic. cinnb. cob. glon. gnap. guare. jac. lil-t. lith. plan. rumx. sep.
— — first right, then left, then to nape where it disappears: jac.
— **side** of: arg-n. (r), asaf. (l), cimic. (l), cyc. (r), fluor-ac. (l). glon.
— **vertex**: æsc-h. alcoh. am-c. apis. calc-p. chin-s. chro-ac. cimic. eup-per. GLON. gymn. ham. helon. hyper. k-bi. lac-ac. meph. osm. pic-ac. psor.
— — as if something were pumped in: glon.

**Conditions** of Fullness.
— **morning**: am-m. arg-n. borax. carl. cann-i. chin-s. chr-ac. cinnb. cob. con. hydr. indg. mag-m. nat-p. nicc. petr. pic-ac. rhus. sul-ac.
— — 10 A. M. to 10 P. M.: lac-ac.
— — on waking: con. glon. kalm. lil-t. tilia.
— **noon** to 2 P. M.: pic-ac.
— **afternoon**: arg-n. coca. ferr. gels. guare. lac-ac. lact. lith. mill. nat-p. osm. phys. sang. sulph.
— — to night: sil.
— **evening**: arg-n. cimic. ferr. guare. ham. naja. nat-m. nat-p. thu.
— **night**: arg-n. chr-ac.
— **air**, in open: grat.
— — — amel.: cinnb. carl. jac.
— — — walking in, amel.: borax. hydr.
— **ascending**, on: borax.
— **breakfast**, after: con. hydr.
— **chilliness**, during: eupi.
— **dinner**, after: gins.
— **eating**, while: con.
— — after: con. gins. hydr.
— — before: uran-nit.
— **hat**, worse from pressure of: calc-p.
— **head**, aching, during: calc-p.
— — on bending back, aggr.: osm.
— — leaning to left: chin-s.
— — on raising, aggr.: sulph.
— — shaking aggr.: *carb.* glon.
— **heat**, during: glon. lach.
— **lying**, when: naja.
— **menses**, on appearance of: gent. *glon.*
— — during: arg-n. *bell. calc.* eupi. *glon.*
— **mental** exertion, from: helon. indg. nat-p. *psor.*
— — better when mind is employed: helon.
— **micturition**, copious, amel.: gels.
— **motion** aggr. from: calc-p.
— **pressure** of hat aggr.: calc-p.
— — amel.: agar. arg-n. hydr.
— **reading**, when: helon. indg.
— **rising**, on: am-m. cinnb. glon. sil.
— **sewing** aggr.: petr.
— **siesta**, after: mill.
— **sitting**, when: borax. glon.
— — up, aggr.: calc-p.
— **sleep**, aggr. after: sulph.
— **sneezing**, on: hydr.
— **stool**, when straining at: ham.
— — amel. after: corn.
— **stooping**, when: acon. lac-ac. merl. nicc. petr. pic-ac. rhus. spong.
— **talking**, after, aggr.: sulph.
— **vertigo**, during: am-m. borax. bry. chr-ac. con. croton. cyc. gymn. helon. lac-ac. lact. merc. nat-m. nat-p. podo. sol-n. tilia. urt-ur.
— **waking**, on: agar. asaf. guare.
— — before: hyper.
— **walking** in open air: thu.
— **wine**, after: ailan.
— **writing**, while: chin-s.

**Gloominess.** See Confusion, Dullness, etc.

**Gnawing**: calc. canth. led. lyc. nat-m. pæon. par. phos. *ran-sc.* znc.
— **forehead**: con. merc-i-r. nat-s. sulph. znc.
— — above right eye, morning: dros.
— — above nose: calc-ac. merc. phos. raph.
— **occiput**: calc. dros. glon. nicc. ol-an. raph.
— **temple**: ran-sc. sol-n. (r).
— — temples, occiput and ears: led.
— **vertex**: men. ran-sc.
— with otalgia: ran-sc.
— at night: merc-i-r.
— — when stepping heavily: lyc.

**Gradually** disappearing pain: op. plat. sabin.
— increasing and suddenly ceasing: sul-ac.
— see also under Increasing.

**Grasping**: ars. con. mag-m. nat-s.
— forehead, amel. by cold foot-bath: nat-s.
— grabbing sensation, during confusion of head: hell.
— grasped forehead and cried my head, my head! I am crazy; amel. walking about: sulph.
— momentary blindness; grasps head, it feels so strangely: nx-m.
— apoplexy, grasps head; mouth drawn to left: phos.
**Grinding**: agar. anac. myric.
— amel. by pressure: anac.
**Griping**: con. mag-s. sep.
**Grumbling**: hepar. indg. sul-ac.
**Gurgling**: asaf. bry. sep.
— see Bubbling.
**Hacking**: am-c. ars. aur. lyc. nitr. ph-ac.
— see Cutting.
**Hair.** See under Scalp.
**Hammering**: *am-c.* ars. aur. calc. chin-s. cic. clem. coff. dros. *ferr.* hepar. indg. *lach.* manc. mezer. nat-m. nicc. nit-ac. ph-ac. psor. rhus. rheum. sil. sulph. tarent.
— — see Pulsation, Shocks, etc.
— — like a clock: mag-s.
— — like a hammer: ars.
— **forehead**: am-m. cic. creos. olnd. verb.
— — as with small, sharp hammer: nicc.
— **temple**: ars. benz-ac. chin. psor.
— **vertex**: hyper. phos.
**Hands**, holds head with: glon. hyos.
— — — — on coughing: nicc. nx-v. sulph.
— leans on: iod.
— puts to: camph. verat.
— rubs with: verat.
— see also under Grasping.
**Heat**: acet-ac. *acon.* æsc-h. æth. agar. all-c. aloe. alum. *ambr.* am-c. am-m. anac. ang. ant-c. ant-t. apis. arg-n. *arn.* ars. asaf. asar. *aur.* bad. bapt. bar. *bell.* benz-ac. berb. bism. borax. brom. *bry.* calad. calc. calc-p. camph. cann-i. cann-s. canth. carb-an. carb-v. caus. cham. chel. chin. chin-s. cimic. cinnb. clem. coff. colch. coloc. con. corn. croc. cupr-ac. cyc. daph. dig. dios. dros. dulc. euph. euphr. eupi. fluor-ac. gamb. gels. gins. glon. gran. graph. grat. gymn. hæmat. hell. hura. hydr. hydr-ac. hyos. hyper. ign. indg. indm. iod. ipec. iris. jatr. k-bi. k-bro. k-ca. k-clc. k-iod. kalm. lach. lact. *laur.* led. lyc. mag-c. mag-m. mag-s. manc. mang. men. merc. merc-c. merl. mezer. mosch. nat-c. nat-m. nicc. nitr. nit-ac. nx-j. nx-m. nx-v. ol-an. op. pæon. petr. phel. phos. ph-ac. phys. pic-ac. plat. plb. podo. puls. ran-b. ran-sc. ratan. rheum. rhus. ruta. sabad. sabin. sars. secale. senec. *sep.* *sil.* spig. spong. squil. stann. staph. stram. stront. *sulph.* tabac. tarent. taxus. therid. tellur. thu. tilia. tong. valer. verat. vinc. viol-od. *xanth.* znc.
— — **agreeable**: camph. cann-s. nicc. thu.
— — **air**, as if surrounded by hot: aster.
— — **alternating**, with chilliness: asaf. sep.
— — — with diarrhœa: bell.
— — — with rigor in back: spong.
— — **anxious**: canth. coff.
— — **coryza**, as from: berb. nx-v.
— — **breath**, like a warm: fluor-ac.
— — **flashes** of: æsc-h. æth. am-m. ant-t. arn. ars. aur. calc-p. cic. cocc. colch. corn. dig. glon. hell. hepar. lact. laur. led. mag-c. mag-s. nat-m. œna. ptel. *sep.* *sulph.* tabac. xanth. ziz.
— — — after chilliness: *sang.* *sil.*
— — **hot** body, as if, fell forwards: k-ca.
— — — iron around, as if from a: *acon.*
— — — water: all-c. indg.
— — — water thrown on scalp and penetrating to brain, as from: petiv.
— — **periodical**: calad.
— — **rising** up: æth. calad. canth. cyc. gamb. nat-s. plb. rheum. rhus.
— — — from abdomen: alum. indg. mag-m. plb.
— — — from back: phos.
— — — from chest: acon. glon. phos. sulph.
— — **spot**, in small: carb-v. mezer.
— — **sweat**, as if, would break out: caus. phos. plat. ran-b.
— — **tormenting** sensation of heat, with dullness: stram.
— — **transient**: agar. arn. cann-i. mag-m. sulph. tabac. valer.
— — **vapor**, as from warm: ol-an.
— — **wine**, as from: rhus-r. sabad.
— **forehead**: *acon.* æth. alum. am-m. ang. ant-t. ars. asaf. asar. bad. bapt. bell. brom. calc. camph. canth. carb-an. carb-v. caus. chel. chin. cimic. cinnb. clem. cocc. coloc. creos. cup-ac. cyc. euph. euphr. eupi. fluor-ac. gins. glon. gran. graph. grat. gymn.

hell. hepar. hydr. hyos. indg. indm. jatr. k-bi. k-ca. lact. laur. led. lyc. mag-m. mag-s. manc. merc. merc-c. mezer. nat-c. nat-m. nicc. nitr. ol-an. op. petr. phel. phos. ph-ac. phys. pic-ac. puls. ran-b. ratan. rhus-r. sabad. senec. sep. sil. spong. stann. staph. stram. sulph. tarax. tarent. taxus. tellur. thu. tilia. verat. viol-od. znc.
— — **alternately** in either protuberance: lact.
— — as if warm water trickled down inside: glon.
— — feeling of warmth in middle of forehead, then coolness as from draught of air: laur.
— **occiput**: æsc-h. bell. brom. camph. cann-i. cann-s. cic. cinnb. cocc-c. con. dig. fluor-ac. glon. indg. jatr. kalm. lobel. manc. merc-i-fl. nat-s. puls. rhus-r. *sulph.* tarent. thu. verat-v. znc.
— — flashes of: æsc-h. lach.
— **side**: am-m. (r), calc. (l), caus. (r), cinnb. cyc. (r.), k-bi. (l), petr. (l), phel. (l), pic-ac. tarent. tilia.
— — in flashes: k-bi.
— **temple**: berb. euph. glon. hura. ign. lyc. merl. ol-an. phel. podo.
— — with cold cheeks: berb.
— **vertex**: acon. *calc.* camph. carb-an. chel. cocc-c. corn. daph. *eup-per.* grat. hepar. hyper. *lach.* laur. lept. mag-s. *merc-i-r.* mezer. nat-s. nx-m. phos. podo. rhus-r. *sulph.* tarent. thea.
— — in small spots: mezer.

**Conditions** of Heat.
— **morning**: alum. am-m. ant-c. ant-t. *bry.* calc. carb-an. chin. clem. cyc. dios. euphr. hipp. hyper. indg. kalm. lyc. *merc-i-r. mezer.* nat-c. nitr. nx-v. petr. phos. podo. sep. *sulph.* tilia. tong. zing. znc.
— — on waking: berb. calc. lyc. nat-m. sil. stann. sulph.
— — on rising: agar. am-m. bar. calc. corn. cyc. dulc.
— — — — amel.: sulph.
— **noon**: ant-c. bell. jatr.
— **afternoon**: ant-t. arg-n. bad. berb. bry. cann-s. carb-an. chin-s. dios. graph. hyper. ipec. lyc. mag-c. mag-m. mag-s. mang. nat-m. nicc. ol-an. phos. phys. *puls.* sep. spong. stront. sulph.
— **evening**: acon. alum. am-m. borax. calc. canth. chel. cocc-c. cyc. grat. indg. k-ca. laur. lil-t. lobel. lyc. mag-c. mag-m. merc-i-r. nat-c. nx-j. nx-v. ol-an. ph-ac. phys. puls. ran-b. *rhus.* sep. sil. sulph. thu. znc.
— **night**: am-m. ang. arg-n. camph. cann-s. lyc. meph. nat-c. rhus-r. ruta. sil. staph. tilia.
— — in bed: carb-an. lyc. nat-m. *sulph.*
— — on waking: tilia.
— **air**, in open aggr.: verat.
— — — — amel.: clem. grat. laur. mag-m. mang. nat-c. phel. phos.
— **abdomen**, from pain in: grat.
— **anus**, from pain at: oxal-ac.
— **anxiety**, with: stront.
— **back**, with coldness of: thu.
— **bed**, when in: ang. arg-n. carb-an. cyc. lyc. nat-c. *nx-v.* staph.
— — — amel. in: k-ca.
— **beer**, after: chel. sulph.
— **bone**, with pains in: merc-c.
— **catarrh**, during: graph.
— **chest**, after heat in: grat.
— **chilliness**, during: ant-c. asaf. colch. hell. mag-m.
— **cold** bath, amel.: euphr. indm. mezer. nat-m. sep.
— **coldness** of body, with: *acon.* ARN. asaf. bufo. calc. chin. chin-s. clem. gels. hipp. hyos. *lachn.* mag-s. nx-v. plb. stram. verat.
— — extremities, with: bell. camph. cann-i. cann-s. chel. com. nx-j.
— — fingers, with: hell.
— **constipation**, during: chin.
— **contradiction**, from: cop.
— **coryza**, during: ARUM-T. calc. jatr. lach. mag-m.
— **cough**, during: am-m. ant-t. *ars.* carb-v. ipec. *sulph.*
— **diarrhœa**, during: oxal-ac. rhus.
— **dinner**, after: alum. berb. caus. cyc. graph. mag-m. phel.
— — during: grat. nat-c. nx-v.
— **eating**, after: alum. bell. berb. canth. carb-v. caus. clem. cyc. graph. hyos. laur. *lyc.* mag-m. *petr.* phel. phos.
— — hot food: mag-c.
— **exertion**, from: berb.
— **eyes**, on moving: chin.
— **face**, with coldness of: thu.
— — with heat of: æth. arg-n. berb. bry. calc-p. cann-s. canth. clem. corn. glon. hura. jatr. iris. k-ca. k-iod. nat-m. nitr. op. phos. sabad. stront. sulph.

— — with pale: ambr. puls.
— — during redness of: æth. bry. cact. cann-s. k-iod. mag-c. mag-m. mag-s. merl. nat-c. phel. plb. stront. sulph. tarent. znc.
— **feet**, with cold: alum. am-c. bar. bell. con. ferr. gels. laur. nat-c. squil. sulph.
— **grief**, after: phos.
— **hands**, with coldness of: asaf. asar. bar. bell. hell. iod. lact. lyc. nat-c. sumb.
— — with heat of: canth. lach. laur. mag-c. ol-an. phel. phos.
— — — — — palms: borax. tarent.
— **headache**, with: apis. hydr.
— — on moving head: calc.
— — on raising head: grat.
— **heart**, during oppression of: glon.
— — with palpitation of: coloc. iod.
— **lying** down, when: arn. ars. nx-j.
— — — amel. when: k-ca. nat-c. rhus.
— **menses**, before: apis. bell. calc. *con.* ign. *iod.* ipec. *lyc.* petr. thu.
— — during: *apis. arn.* bell. *calc.* carb-an. caus. cham. *ign.* ipec. *k-iod.* lyc. mag-c. mag-m. mag-s.
— **mental** exertion, from: anac. aur.
— **motion**, on: ant-t. spong.
— — amel.: phos.
— **nausea**, with: cann-s. dig. gran. jatr. nat-m. phyt. stann.
— **pressure**, amel.: arg-n. hydr. nx-v.
— — when touched: berb.
— **reading**, when: nat-s.
— **riding**, when: lyc.
— **rising**, on: bar. calc. mag-s.
— — from stooping: grat. nat-c.
— — amel. on: carb-an. k-ca. sulph.
— **room**, when in: caus. clem. cocc-c. indg. mag-m. nat-c. nicc. ran-sc. *sulph.*
— — in hot: cocc-c. sulph.
— **sewing**, when: petr.
— **shivering**, with: ARN. ars. ran-b.
— **siesta**, after: clem. cyc. rhus.
— **sitting**, when: canth. merc. nat-c. ph-ac. spong.
— **sleep**, before going to: almn. cocc-c.
— — amel.: laur.
— — with sleepiness: creos. stann. stront.
— **sneezing**, from, amel.: lil-t.
— **soup**, on taking: phos.
— **speaking**, by: phos. ph-ac.
— **standing**, amel. when: phos.
— **stool**, during urging to: clem. mag-m.
— — after: lyc.
— **stooping**, when: k-ca. petr. valer.
— **storm**, on approach of: nat-c.
— **throbbing**, with: *glon.* spong.
— **vertigo**, with: clem. dios. hydr-ac. lact. merl. nit-ac. op. ph-ac. puls. spong. sabin.
— **waking**, on: calc. chel. lyc. nat-m. phos. sil. stann. sulph. tarent. tilia.
— — before: hyper.
— **walking**, when: borax. glon. indg. mezer. nit-ac. sep. stront.
— — in open air, amel.: sulph.
— **wine**, after: lyc. nx-v. petr.
— **writing**, on: k-ca. ran-b.

**Heaviness**: acet-ac. *acon.* æsc-h. æth. *agar.* agn. ailan. all-c. aloe. alum. ambr. am-c. *am-m.* anac. ang. ant-t. apis. apoc. *arg-n. arn. ars.* arum-t. asaf. asar. aur. bapt. bar. bar-ac. bar-m. *bell.* berb. bism. borax. bov. brom. *bry.* bufo. cact. *calc. camph.* cann-i. canth. carb-an. *carb-v.* cast. caus. cedr. *cham.* chel. *chin.* chin-s. cic. cimic. cinnb. clem. coca. cocc. cocc-c. coff. coloc. con. corn. cop. creos. croc. crotal. croton. *cupr-ac.* cyc. dig. dios. dros. *dulc.* euphr. eupi. ferr. ferr-i. fluor-ac. gamb. *gels.* gins. glon. gran. graph. grat. guare. gymn. hæmat. *hell.* hepar. hipp. hura. hydr. hydr-ac. hyos. hyper. ign. indg. iod. *ipec.* iris. jatr. k-bi. k-ca. *k-iod.* lac-can. lach. lachn. lac-ac. *laur.* led. lil-t. lobel. *lyc.* mag-c. *mag-m. mag-s.* manc. mang. *men.* meph. *merc.* merc-c. merc-i-fl. merc-i-r. *merl.* mezer. morph. mosch. murx. mur-ac. naja. nat-c. *nat-m.* nat-p. nat-s. *nicc.* nit-ac. nitr. nx-m. *nx-v. olnd.* ol-an. *op*-osm. pæon. par. petr. *phel. phos. ph ac.* phys. phyt. pic-ac. plan. plat. plb. prun. ptel. *puls.* ran-b. ran-sc. ratan. *rheum. rhus.* ruta. sabad. *sabin.* sang. sars. *secale. seneg. sep. sil.* sol-n. spig. *spong.* squil. *stann.* stram. stront. *staph. sulph.* sul-ac. *tabac.* tarax. tarent. tellur. tereb. thea. therid. *thu.* tilia. *tong.* ustil. valer. verat. *verat-v.* verb. viol-od. viol-tr. vip. znc. zing.
— — compare with Pressure.
— — **alternating** with clearness of mind: murx.

— — **blood**, as if too full of: glon. ign. lil-t.
— — **coryza**, as if from: berb. laur.
— — **dizzy**: camph. bry. nat-m. nx-v.
— — **drawn**, backward, as if: phel. znc.
— — — forward, as if: olnd. viol-tr.
— — — to one side, as if: sil.
— — **drinking**, as if had been: acon. agar. cocc. dulc. ferr. lach. laur. nitr. sabin.
— — **dull**: apoc. caj. calc. fluor-ac. glon. nat-s. phys. rumx. verb.
— — **fall**, backward, head: camph. chin. k-ca. phel.
— — — **forward**, as if brain would: carb-an. laur. rhus. sul-ac.
— — — forward, as if head would: agn. alum. bar. berb. hipp. nat-m. op. par. phos. plb. rhus. sulph. sul-ac. tabac. viol-tr. znc.
— — — **down**, as if brain would: alum. bell. berb. hipp.
— — — **side**, to one, as if head would: bry. fluor-ac. phel.
— — **lying** with head too low, as if had been: *phos.*
— — **oppressive**: *ars.* ipec. sabin. therid.
— — **painful**: gran. hell. nicc. olnd. sabad. verb.
— — **pressed** forward, as if brain were: bry. canth. laur. thu.
— — brain feels compressed: hyper.
— — like a weight on brain: chel. nx-v. sil.
— — — — — on head: cocc. phel.
— — **raise** from pillow, as if he could not: iod. ph-ac. puls. sabad.
— — **sleep**, as if had not had enough: mag-c. znc.
— **forehead**: *acon.* æth. agar. ailan. all-c. am-c. *am-m.* ang. ant-c. ant-t. apoc. arg. arn. ars. arum-t. asaf. asar. aspar. bapt. bar. bell. berb. *bism.* bov. brom. bry. bufo. *calc.* camph. cann-i. canth. carb-an. carb-v. cham. chel. chin. chin-s. cic. clem. coloc. con. conv. creos. crotal. croton. dulc. elaps. ferr. ferr-i. gamb. gels. gins. glon. gran. grat. hæmat. ham. hell. hepar. hipp. hura. hydr. hyos. indg. jatr. k-bi. k-ca. k-iod. lac-can. lach. laur. led. lil-t. lith. mag-c. mag-m. mag-s. mang. merc. merc-i-r. mur-ac. naja. nat-c. nat-m. nicc. nitr. nx-v. olnd. op. oxal-ac. pallad. phos. phyt. plb. rhod. rhus. ruta. sabin. sars. sep. *sil.* sol-n. *stann.* staph. stront. sulph. tarent. taxus. tellur. thea. tong. verat. znc.
— — as if full of lead: clem. lyc.
— — — — stone lay there: ruta.
— — — — all would come out: acon. creos. mag-s.
— — — — a weight pressed forward in; must hold head upright: *rhus.*
— — — — — — sank down in it: nx-v.
— **occiput**: æsc-h. æth. agar. ant-t. apis. aur. *bell.* bism. bov. *bry.* cact. calc. caj. cann-s. canth. *carb-an.* cham. CHEL. chin. cic. clem. colch. con. cop. creos. dulc. eup-per. ferr. gins. hell. indg. k-ca. k-iod. *lach.* lac-ac. lact. laur. lyc. mag-m. mang. *meph.* mezer. *mur-ac.* myric. *nat-m.* nat-s. nicc. nitr. op. pæon. petr. phos. ph-ac. plb. prun. ptel. ruta. sabin. secale. selen. sep. spig. spong. stann. *sulph.* tarax. thu. tilia. trill.
— — eyes, seems to draw together: nat-m.
— — **extending** downward: sep. sulph.
— — — from ear to ear: ferr.
— — — to shoulders, after waking, while lying on back: bry.
— — **lead**, as if full of: k-ca. lach. mur-ac. op. petr. spong.
— — **raise**, difficult to: chel.
— — — pain in occiput like a weight, must raise head with hands: eup-per.
— — **sink**, as if head would, backward: ign. k-ca. mur-ac. op.
— — step, at every, a jolt as if a weight were on occiput: bell.
— **side** of: æth. (r), am-c. (l), arg-n. (r), bov. (r), *cact.* (r), cedr. elaps. (r), eugen. (r), grat. (l), hydr. kalm. (r), k-ca. (l), k-iod. lyc. (l), mag-m. (l), sabad. (r), sabin. (l), stann. (l), sul-ac. (l), tarent. (l).
— **temple**: agar. bell. bism. bov. cact. carb-an. cimic. cinnb. clem. ferr. glon. k-iod. led. nit-ac. phyt. rhus. sabad. sars. sep. stann. tellur.
— — as if a weight hung at both sides: agar. rhus.
— **vertex**: acon. all-c. *aloe.* alum. apis. bry. CACT. camph. *cann-s.* canth. cast. chel. chin-s. con. dig. eup-per. hell. hipp. hydr. indg. k-bi. k-iod. laur.

mag-c. merc-i-r. mosch. oxal-ac. *phel.* phys. phyt. pic-ac. rhus. squil. stann. *sulph.* xanth.
— — as from a stone or weight: cann-s. indg. mosch. phel.

**Conditions** of Heaviness.
— **morning**: acon. agar. alum. am-m. ars. arum-t. berb. bov. bry. calc. *carb-an.* cast. chel. chin. cimic. clem. coca. com. con. croc. eupi. gamb. hell. hydr. hyper. indg. k-ca. k-iod. kalm. lach. lyc. mag-m. mang. mezer. nat-m. nicc. nitr. *nx-v.* op. oxal-ac. pæon. pallad. petr. phos. phys. phyt. pic-ac. plb. ruta. sabin. sars. sep. sil. spig. sulph. sul-ac. tarent. verat. znc.
— — on waking: bar. bell. bry. calc. calc-p. cann-i. cham. chin. croc. croton. euphr. ferr. fluor-ac. lach. lil-t. lyc. mag-s. nat-m. nicc. nit-ac. phos. rhus. sol-n. squil. tarent. tong. verat.
— — on rising: am-m. anac. ang. ars. aur. bell. clem. cocc-c. coff. hell. hipp. hura. k-bi. k-iod. mag-c. mag-m. nat-m. nicc. phos. rhod. sep. stront. sulph. tong.
— — — amel. after: k-iod. mag-s. nicc.
— **afternoon**: all-c. alum. am-c. *arg-n.* bry. bufo. cham. chel. chin-s. ferr. gamb. gels. hyper. indg. k-iod. lact. mag-c. mag-m. mang. murx. nicc. nx-j. pallad. puls. sil.
— **evening**: ambr. apoc. arg-n. ars. bar. bov. bufo. cedr. chin-s. coloc. ferr. fluor-ac. hydr-ac. k-iod. kalm. laur. lith. lyc. phos. plan. rumx. sep. *stann.* sulph. znc.
— **night**: arg-n. carb-an. k-iod. lil-t. mezer. nit-ac. sil. tarent. tilia.
— — on waking: chel. cic. nat-c. tilia.
— **abdomen**, with distention of: graph.
— **air**, in cold: carb-an.
— — in open: laur. lil-t.
— — — amel. in: ant-t. ars. caus. clem. ferr-i. gamb. hell. hydr. mang. mosch. nicc. phos.
— **ascending**, on: men. rhus-v.
— **back** and limbs, with pain in: apoc.
— — with drowsiness and pain in: gamb.
— **bed**, when lying in: am-c.
— **beer**, after: chel.
— **breakfast**, amel. before: *nat-m.*
— **candle-light**, from: bov.
— **chill**, during: sulph.
— — after: dros.
— **coffee**, strong, amel.: corn.
— **cold**, after taking: dulc.
— — amel.: chin-s.
— **coughing**, on: euphr. taxus.
— **daily**: nat-m. sil.
— **darkness**, amel.: brom.
— **descending**, on: men.
— **diarrhœa**, with morning: lil-t.
— **dinner**, amel. after: carb-an.
— **eating**, while: æth.
— — after: am-c. bry. cast. cedr. euphr. gins. graph. grat. k-iod. mag-c. mag-s. nat-c. nat-m. nx-j. nx-v. op. tabac.
— **epistaxis**, amel.: dig.
— — accompanied by: nat-s.
— **erect**, on becoming, aggr.: con.
— **exercise**, aggr.: calc.
— **eyes**, on moving: chin. nx-v. *rhus.*
— — exertion of, aggr: mur-ac.
— — with burning, after dinner: nat-c.
— **fever**, during the: sep. thu.
— — after the: tarent.
— **forehead**, wrinkling of, amel.: phos.
— **head**, bending back. amel.: *cocc.* ph-ac.
— — — forward, on: nat-m. ph-ac.
— — holding erect, on: dros. tarax.
— — lying with, head high, amel.: sulph.
— — moving, on: asar. calc. indg. sars. spig.
— — raising, on: dros. ign. op. spong. sulph.
— — — amel.: bry.
— — shaking the, partially amel.: gels.
— — supporting, amel.: staph.
— — stupid feeling in, with: canth. cic. coloc. crotal. ferr. hell. hepar. indg. k-iod. laur. mag-m. mezer. nicc. nitr. nx-v. op. plb. puls. sars. sol-n. sulph. tabac.
— **heat**, from: com. hell.
— — of sun: brom. nat-c.
— **legs**, with tingling in: secale.
— **light**, worse from strong: cact.
— **looking** steadily, amel.: sabad.
— — sideways, when: agn.
— **lying** down, when: am-c. bov. glon. mag-c. merc. nicc. nx-m. puls. sep. sulph. *tarax.*
— — — amel.: *nat-m.* olnd. rhus. tellur.

— — on back: cact. mezer.
— — on side, when: men.
— — — — amel.: cact.
— — with head high, amel.: sulph.
— **menses**, before: cimic. crotal. ign.
— — during: calc. carb-an. *ign.* *k-ca.* *mag-c.* *mag-s.* nx-v. znc.
— — after: all-s. nat-m.
— — — menstrual colic: ant-t.
— **mental** exertion, from: *calc.* crotal. ferr-iod. lyc. nat-m. phos.
— **motion**, from: acon. arg-n. bism. bov. *calc.* canth. colch. fluor-ac. lyc. phys. plat. *sars.* *stann.* sulph. thu.
— — amel.: mag-c. mosch.
— **pressure**, amel.: ailan. CACT. camph. cop. mur-ac. nat-m. sabin.
— **reading**, when: bry. calc. croton.
— **rest**, during: stann.
— — amel.: arg-n.
— **riding**, when: phyt.
— **rising**, on: am-m. ang. aur. bapt. calc. hura. iod. olnd. sulph. tarax. viol-tr.
— — amel. on: calc. con. laur. nicc.
— — from stooping, on: grat. mag-s. sulph. tong. viol-tr.
— **room**, when in: ars. laur. merc. phos. rhus.
— — warm, room: chin-s. hydr. pæon.
— **salivation**, with: fluor-ac. phos.
— **sewing**, when: petr.
— **siesta**, after a: bov. bry. mag-c. rhus.
— **sleepiness**, with: canth. gamb.
— **sitting**, when: æth. alum. ang. ars. caus. chin. cic. merc. olnd. squil. sulph.
— — bent over, when: *con.*
— — amel.: sulph.
— **sleep**, amel.: laur.
— **sleepiness**, with: apoc. asaf. bar. bell. coca. corn. gamb. grat. hydr. ipec. laur. nit-ac. op. rhus-r.
— **smoking**, aggr.: ferr-i. gels.
— **soup**, after taking: phos.
— **sneezing**, on: seneg.
— **standing**, on: alum. ars. bov. caus. k-ca. mag-c. nicc. plb.
— **stool**, after: apoc.
— **stooping**, on: acon. alum. *berb.* bov. bry. camph. *carb-an.* colch. con. fluor-ac. grat. hell. hyos. indg. k-bi. k-iod. laur. nat-m. nicc. nit-ac. *nx-v.* petr. phos. ph-ac. plat. *puls.* rhus. senna. spong. sulph. sul-ac. tabac.
— — amel.: dros. ign. tarax. viol-tr.
— — **after**: tong. viol-tr.
— **sun**, heat of, in: brom. nat-c.
— **swallowing**, aggr.: k-ca.
— **sweat**, during: ars. caus.
— **talking**, from: cact. nat-m. sulph.
— **touch**, on: agar.
— **urine**, profuse discharge, amel.: *gels.* fluor-ac.
— **vertigo**, during: acon. am-m. bov. bry. carb-an. caus. clem. coff. gels. hepar. hydr-ac. k-bi. lact. laur. mag-c. mag-m. mag-s. naja. nat-c. nicc. nx-m. pæon. phos. prun. puls. secale. thu. tilia.
— **vexation**, after: mag-c.
— **vomiting**, after: sulph.
— — amel.: tabac.
— **waking**, on: bar. bell. bry. calc. calc-p. cann-i. cham. chel. chin. cic. con. croton. euphr. ferr. fluor-ac. ign. lach. lil-t. mag-s. nat-c. nat-m. nicc. nit-ac. rhus. sep. sol-n. squil. sulph. tarent. tilia. tong. verat.
— **walking**, when: hell. hipp. k-bi. laur. puls. rheum. rhus. spong. sulph. thea.
— — amel.: k-bi. mag-c.
— — after in open air: bov.
— — in open air, amel.: hydr.
— **washing**, amel.: mag-c. phos.
— **work**, from fatiguing: nat-c.
— **writing**, when: calc. gent-l. lyc.
— **yawning**, with: mag-c.

**Heaving** up and down sensation in: *bell.* con. lyc.

**Hold** up head, unable to: ant-t. atro. bapt. cham. con. croc. cupr-s. gels. glon. hipp. lil-t. lyc. mang. mezer. nx-v. olnd. op. petr. phel. *puls.* rhus. sabad. *sil.* tabac. znc.
— steady, unable to: squil.

**Hollow.** See Empty.

**Humming.** See Buzzing.

**Hysterical** headache: *aur.* bell. bry. caps. cham. cocc. hepar. *ign.* k-bi. lach. mag-c. mag-m. *mosch.* nit-ac. phos. *plat.* rhus. ruta. *sep.* valer. verat.
— ill-humor, with: bov. creos. k-ca. *plat.* sil.
— impatience, with: plat.
— inability to collect one's self, with: carb-v. chin. creos. crotal. cyc. mang. mezer. nit-ac. rhus. sars. sil. stann. sulph.

**Increasing** gradually, pain: acon. bry. lact. lobel. sars.

— and decreasing, gradually: arn. bar. crotal. jab. k-bi. op. *plat.* sabin. sars. *stann.* stront. verb.
— and decreasing, rapidly: arg-n. bell. coca. merc-c. sul-ac.
— gradually, but suddenly going off: arg. caus. sul-ac.
**Inflammation** of brain: *acon.* apis. *bell. bry.* cadm. *camph.* canth. cham. cina. crotal. *cupr.* glon. *hell. hyos.* lach. merc. nx-v. phos. plb. puls. rhus. stram. sulph. verat-v.
— — with sopor: borax.
**Intermittent** pains: agar. anac. arg. cann-i. caul. cina. cupr-ac. ign. iod. iris. kalm. mill. nit-ac. plan. plat. psor. sang. sep. stann. tereb. valer. verat.
**Intoxication**, as from: æsc-h. agar. am-c. ars. berb. bry. carb-an. carb-v. caus. chel. creos. croc. croton. cyc. euphr. eupi. glon. graph. hydr-ac. iod. laur. mag-m. mezer. nat-m. nit-ac. nitr. *nx-v.* par. ph-ac. ptel. *puls.* rhod. *rhus.* samb. spig. tarax. sul-ac. valer.
— — compare with Confusion.
**Itching**: dig. sabad. tarax.
— see under Scalp.
— sides and inside: dig.
— **forehead**: agar. am-m. anac. ars. bell. berb. bov. canth. caps. caus. cham. clem. con. fluor-ac. gamb. gran. hura. hyper. k-bi. lach. laur. led. lyc. mag-c. merc. nat-m. olnd. ol-an. petr. *rhus.* samb. sars. spig. squil. *sulph.* tabac. verat.
— — evening: *sulph.*
— — **air**, amel. in open: gamb.
— — **dinner**, during: hepar. mag-c. sulph.
— — **menses**, before, itching eruption on: sars.
— — **rubbing**, amel.: ol-an. samb. tabac.
— — **scratching**, amel.: bov. mag-c. squil.
**Jerking** pain: acon. ambr. am-c. anac. ant-t. *arn.* asaf. bar. bell. bism. borax. *bry.* calc. cann-i. canth. carb-an. carb-v. caus. *chin.* creos. croton. cyc. dulc. glon. graph. ign. k-ca. lach. lyc. mag-c. mag-m. marum. men. merc. mill. mur-ac. nat-c. nat-m. nit-ac. nx-v. pæon. petr. phos. ph-ac. plb. prun. *puls.* ratan. sabad. sep. sil. spong. squil. stann. sulph. thu.
— — compare with Shocks.
— — here and there: chel. stront.
— — involuntary jerking of head back and forward, when sitting: sep.
— — from one side to another: samb.
— — from behind forward: k-ca. ph-ac.
— — on walking quickly or ascending stairs: bell.
— — throwing head toward the right: nat-s.
— **forehead**, in: arn. borax. cann-i. caus. cham. chin. op. sep. sil. stann. sulph. sul-ac. thu.
— — **across**: sabad.
— — alternating with dull aching: stann.
— — backward: prun.
— **occiput**, in: acon. bell. cedr. fluor-ac. glon. k-ca. prun. rhus. spig. stann. thu.
— — forward: arg-n.
— — intermitting: canth.
— **sides** of: æth. (l), alum. caus. chin. nat-m. nicc. nit-ac. sabin. (r).
— **temples**, in: arn. cast. chin. glon. k-ca. lact. lil-t. oxal-ac. plb. stann. (l), sulph. valer.
— — downward: anac.
— — upward: am-m. spong.
— **vertex**, in: anac. k-iod. men. mur-ac. ran-sc. spong.
— — here and there: k-iod.
— **drinking** cold water amel.: k-ca.
— worse during rest, better from motion: stann.
**Knocks** head against things: ars. bell.
— pain as if knocked in the head: mosch.
— — see under Bruised.
**Knocking** in: am-c. ang.
— — like a ball striking skull: plat.
— — as of brain against skull: ars. chin. daph. glon. hydr-ac. laur. mezer. nat-m. nx-m. rheum. stann. sulph. sul-ac.
— — — — on motion: nx-m. *rhus.*
— — — — see also under Pulsation, and compare with Motion, etc.
**Lacerating**. See Tearing.
**Laming** pain: cina.
**Lancinating** pain: acon. æsc-h. ambr. am-c. arn. bell. cadm. cup-ac. dros. gins. graph. hepar. hura. ipec. k-iod. manc. sang. squil. tarent.
— see also Cutting.
— motion, amel.: k-iod.

— stooping, on: arn.
— vertigo, with: nat-m.
— walking in open air, amel.: hepar.
**Large**. See Enlarged, and compare with Swelling, etc.
**Lean** on to something, desires to: *bell.* gymn.
— — see also Falling; compare with Heaviness.
**Lightness** in, a sense of: acet-ac. am-c. arum-t. bar. bry. camph. cann-i. chin-s. chr-ac. cinnb. coff. con. corn. crotal. eup-pur. gels. grat. *hipp.* hydrph. iris. k-bi. k-ca. lach. lac-ac. naja. op. oxal-ac. phos. sars. secale. sol-n. *stram.* trom. ziz.
— — compare with Empty.
— — as if air were in head: benz-ac.
— — as if borne by air: op.
— **occiput**, in: secale.
— **giddiness**, after: iris.
— **motion**, aggr.: gels.
— **nausea**, after: hydrph.
— **sitting**, when: carb-an.
— **sneezing**, when: benz-ac.
— **walking**, when: oxal-ac.
**Load**, pain as from a: bell. led. men. *mosch.* ph-ac. plat. rhus. sars. sil. *spig.* squil. sulph. *thu.*
— compare Heaviness, Pressure, etc.
**Looseness** of brain, sensation of: acon. am-c. *bar.* carb-an. caus. chin. cic. con. cocc. croc. elaps. glon. graph. guai. hyos. k-ca. kalm. lach. lact. laur. mag-s. mur-ac. *nat-m.* nat-s. nicc. nitr. *nx-m.* phys. *rhus.* sep. staph. sul-ac. verat. xanth.
— — compare with Motion, Shaking, Vibration, etc.
— — diagonally across top, when turning: kalm.
— — feels as if brain fell to side on which leans: am-c.
— **forehead**, in: chel. con. laur. sul-ac.
— **temple**: sul-ac.
— — when stooping feels as if brain fell toward left: nat-s.
— **occiput**: staph.
— **carrying** a weight, when: mur-ac.
— **head**, on shaking: bar. con. glon. nat-m. *nx-m. rhus.* xanth.
— — on moving the: mag-s.
— — on turning the: k-ca. kalm.
— **step**, at every: guai. rhus.
— **stooping**, when: laur.
— — and rising: phos.
— **waking** in morning, on: cic.
— **walking**, when: acon. carb-an. cob. croc. mag-s. nx-m. nx-v. rhus. staph. verat.
— — in open air, when: caus. sul-ac.
**Lump**, sensation as of, in: ant-t. arn. cham. chel. staph.
— in forehead: cham. pip-m. staph.
**Lying** on something hard, as if: ph-ac.
— too low, as if from: phos.
— in an uncomfortable position, as if: cimx. clem. lyc.
— must lie: k-bi. rhus.
— wants to lie low: absin. mosch.
**Maddening** pains: *acon.* chin. ign. indg. nat-m. puls.
— feeling in brain: plan.
— pain, extending into teeth and face: puls.
— sensation, as of madness: bell.
**Marble**, brain seems as if changed to: cann-i.
**Motions**, movement in: *acon.* aloe. alum. am-c. ang. ant-t. ars. *bar.* bell. *bry.* calc. carb-an. caus. chin. cic. cob. croc. crotal. cyc. dig. eugen. glon. graph. guai. hyos. indg. k-ca. lach. lact. *laur.* mag-s. mezer. mosch. nat-m. nx-m. nx-v. phel. phos. plat. rheum. rhus. sep. sol-n. spig. staph. stront. sulph. sul-ac. tabac. verat.
— compare with Knocking, Looseness, Rolling, Shaking, Undulation, etc.
— **alive**, as if something: crotal. hyper. petr. sil.
— **moved**, as if something, from back of neck up to head: glon.
— **worm**, as of a: alum.
**Conditions** of Motions.
— **morning**: grat. guai. indg. lact. nat-s. spig. tabac.
— — on awaking: cic.
— — on rising: bar.
— **afternoon**: graph. mag-m. mezer. nat-m. sulph.
— — amel. in: bar.
— **evening**: eugen. mag-m. nat-m. plat. stront. sulph.
— **night**: hyper. puls.
— — on awaking: par.
— **air**, in open: laur.
— — amel. in: indg. mag-m.
— — walking in, when: aloe. caus. plat. rhus.
— **ascending** stairs, when: bell. crotal. lyc. nat-m. par.
— **coughing**, when: *bry.* lact. mag-s.

— **drawing** load, when: mur-ac.
— **drinking**, when: acon. bry.
— **eating**, after: alum. mag-s.
— — amel. after: aloe.
— **head**, by bending the: asar. dig.
— — — amel.: spig.
— — **moving**, by: am-c. ars. bar. calc. cocc. con. croc. glon. k-ca. lach. lact. mang. mezer. nat-m. nx-m. rhus. sep. sol-n. spig. squil. stann. sulph. sul-ac. thu.
— — **turning** the, quickly, when: nat-ars.
— **leaning**, when: cyc.
— **menses**, during: mag-m.
— **motion**, from: acon. ars. bry. calc. carb-an. cic. cob. croc. led. mag-c. mag-s. mang. nat-m. nx-m. nx-v. spig. staph. sulph. tabac.
— — amel.: petr.
— **pressure**, amel.: bell.
— **rest**, from: lach. staph.
— **rising** up, when: cham. indg. lyc. phos.
— — amel.: alum. laur. mill.
— **room**, when in: indg. mag-m.
— — in warm: lact.
— **sitting**, when: grat. sil.
— — amel.: spig.
— **speaking**, when: acon. cocc. znc.
— **standing**, when: mang.
— **step**, making a false: led. thu.
— **stool**, when at: spig.
— **stooping**, on: ant-t. alum. berb. bry. carb-an. coff. dig. hydr-ac. k-ca. laur. mag-s. mill. nat-s. nx-v. rheum. rhus.
— **stumbling**, from: bar. led. sep. sil.
— **thinking** about it, amel.: cic.
— **turning**, when: cham. glon. kalm. spig.
— **vomiting**, from: eugen.
— **waking**, on: phos.
— **walking** when: bell. carb-an. cic. cob. cocc. crotal. guai. hyos. indg. lyc. mag-c. mag-s. nuph. nx-v. rhod. rhus. spig. sulph. verb. viol-tr.

**Motions** of head (shaking, nodding, waving, etc.): aloe. ars. aur-m. bell. benz-ac. bry. bufo. cann-i. caus. *cic.* crotal. mezer. nx-m. secale. sep. stram. tarent.
— — — see also Drawn, Falling, Trembling, etc.
— — **backward** and forward: aur. cham. lam. lyc. ph-ac.
— — **constant**: ars. cocc.
— — **convulsive**: camph. cocc. nx-m. stram. tarent.
— — — convulsively, so that talking and swallowing are impossible: nx-m.
— — **difficult**: hipp. k-iod. stann.
— — **forward**: nat-m.
— — **hither** and thither: ars. op. stram.
— — **impossible**: spig. tarent. znc.
— — **involuntary**: merc. nat-m.
— — **pains**, moves head to relieve: chin. k-iod. secale.
— — **pendulum-like**: cann-i. secale.
— — **raising** from the pillow: stram.
— — — and sinking alternately: bell.
— — **rolls** head: hyos.
— — — day and night, with moaning: hell.
— — **rubs** against something: tarent.
— — **sideways**: aur. bell. clem. hell. lyc.
— — — rocks head from side to side to relieve pain: k-iod.

**Nail**, pain as from a: *agar. arn.* carb-v. *coff.* evon. hell. *hepar. ign.* lach. nat-m. *nx-v.* ptel. *puls.* ruta. staph. thea.
— — compare with Plug, and see under Pressure.
— **forehead**: hell. sabin. *thu.* (l).
— — were driven from occiput to forehead: mosch.
— — **side** of: acon. agar. chel. *coff.* hepar. (r), *ign. nat-m.* (l), nx-v. ruta. staph. thu. (r).
— — were driven outward, better lying on part: ign.
— **temple**: *arn.* cocc. *ign.* k-iod. sang. (l).
— — from temple to temple: ham.
— **vertex**: evon. hell. hura. manc. nicc. *nx-v.* staph. *thu.*
— — pain as if a bolt were driven from neck to vertex, worse at each throb of heart: *cimic*
— **air**, aggr. in open: coff.
— **morning**, on rising, worse: ptel.
— **liquor**, after excess of: ruta.
— **menses**, during: arn. *ign. nx-v.*
— **walking** in open air: *thu.*

**Noise** in: ars. bar. calc. caus. chin-s. con. dig. graph. k-ca. lyc. mur-ac. nit-ac. phos. ph-ac. sep. stann. sulph. tarent.
— See also under Ears.
— Compare Boiling, Bubbling, Buzzing, Cracking, Gurgling, Roaring, etc.
— **barrel**, tones like an empty: pimp.
— **chirping** in, like a: agar. ang. bry.
— **crepitation**: acon. calc. cann-i. puls.
— — synchronous with pulse: coff. puls.
— **explosion**, like an: cann-i. dig.
— **gong**, like a, when lying: sars.
— **simmering**, like boiling water: bar.
— **sudden** crashing noise in head, on falling asleep, awakes with a start: dig.
— **ticking**, like a watch or clock: *graph.* mag-s.
— **twang**, as upon a harp string: lyc.
— — — — upon a loose wire: phel.
— **whirlwind**, like a: croc.
— **whirring** in: k-ca. lact. puls.
— **whizzing** in: k-ca. pimp. lyc.
**Nodding** of: alco. aur-m. calc. caus. k-bi. lyc *nat-m.* ph-ac.
— — while writing: caus. ph-ac.
**None**, sensation as if had: asar. calc-iod. nit-ac
**Numbness**, sensation of: acon. ant-t. apis. bapt. bell. bry. calc. carb-an. coff. colch. con. dig. dios. fluor-ac. graph. ham. hura. jatr. lach. lil-t. mag-m. meph. merc. merc-i-fl. merl. mezer. mur-ac. nit-ac. olnd. ol-an. petr. phos. phys. *plat.* sil. stram. thu.
— — compare Asleep, as if.
— — as if tied up in a hot cloth: petiv.
— — as after electric shocks: fluor-ac.
— — during menses: plat.
— — as if of wood: petr.
— **brain**, as if in: mag-c. plat.
— **forehead**: bapt. brom. coll. dig. fluor-ac. ham. mag-m. merc. mur-ac. *plat.* sil. valer.
— — extending to nasal bone: *plat.*
— — as if a board lay there: acon.
— — as if from a blow: plat.
— — morning on awaking and when lying; better from exercise and wrapping head warmly: mag-m.
— **occiput**: bry. gels. merc-i-fl. merl. plat. raph. tellur.
— — as if too tightly bound: plat.
— — **side** of: calc. (r), chel. (r), cina. (r), con. hura. (l), hydrph. (l), lach. (l), ol-an. (l), tarax. thu. (l).
— **temple**: myric. (r), phys. *plat.* zing.
— **vertex**: pallad. plat.
— — preceded by a feeling as if scalp and brain were contracted; better from motion and in open air: plat.
**Open**, pain as if: guare. sil.
— opening and shutting, pain as if: cann-i. cimic. cocc.
**Oppression**, See Heaviness, Pressure, etc.
**Pain**. See Aching.
**Paralysis** of brain, incipient: am-c. ars. carb-v. hyos. lyc. op. phos. plb. znc.
**Paroxysmal** pains: acon. agar. arn. dig. lyc. mur-ac. plat. sil. *spig.* spong. stann. stront. thu. valer. verat.
**Pecking**: carb-an. mosch. nx-v. rhus. ruta.
— **forehead**: carb-an.
**Piercing**: apis. calc. cann-s. carb-an. carb-v. caus. chel. creos. croc. glon. hell. merc. par. petr. phos. sep. sil. staph.
— **temple**: apoc. (r), caj. (r), calc-i. (r), euphr. (r), guai. (l).
— — pressure aggr.: *verb.* (r).
— — compare Cutting, Shooting, etc.
**Pimples** on head. See Scalp.
— on forehead: agar. alum. ambr. ars. bell. bov. bry. calc. *calc-p.* canth. carb-v. clem. con. creos. cyc. gran. hura. indm. k-bi. k-bro. lach. *led.* meph. mezer. mur-ac. nat-c. nit-ac. olnd. par. phos. puls. rhus-v. *sep.* sol-n. *sulph.* tabac. tarent. znc. ziz.
— — burning: bell. canth.
— — itching: alum. calc. *sulph.* ziz.
— — painful: indm. staph.
— — red: bell. carb-v. nat-m. sol-n.
— — sore to touch: ph-ac.
— — smarting, when washed: nx-v.
— — white: carb-v. k-bro. sulph. znc.
— — after drinking wine: znc.
**Pinching**: bar. caus. colch. lyc. marum. mezer. nx-v. *petr.* phos. sil. verb.
— **forehead**: acon. anac. calc. eugen. mezer. nit-ac. nx-m. psor. rheum. staph. verat.
— — extending to root of nose: op.
— **occiput**: am-m. carb-v. chel. hipp. petr.
— **side** of: calc. (l), crotal (l), sep. squil.
— **temple**: crotal. (r), *k-ca.* (l), merc. (r), mezer. olnd. (r), sulph. VERB.
— — as with forceps: ph-ac. verb.

— — extending to ear: nat-p. (1).
— after sleeping: rheum.
— on walking: sil.
**Pithy**, sensation as if upper part of head were: mezer.
**Plug**, peg, or wedge, pain as from a: *anac.* arg. asaf. bov. cocc. con. creos. dulc. hepar. jac. olnd. plat. prun. ran-sc. rhod. rhus. ruta. *sul-ac.*
— — Compare with Nail.
— — as if split wide open by a wedge, body cold, head burns, cannot get warm; whines: lachn.
— — a plug were thrust suddenly in by increasingly severe blows: sul-ac.
— **forehead**: anac. (r), asaf. jac. sul-ac. (1).
— **occiput**: *arg.* rhod.
— **side** of: *asaf.* hepar. (r), *plat.*
— **temple**: *anac. asaf.* sul-ac.
— **vertex**: intense pain as if a bolt were driven from neck to vertex, worse at each throb of heart: *cimic.*
**Pressive** pain. See Boring and under Pressure.
**Pressure**, pressing, etc.: acon. agar. agn. all-c. aloe. alum. *ambr.* am-c. *am-m. anac.* ang. *ant-t.* apis. arg. arg-n. *arn.* ars. arum-t. *asaf.* asar. aur. bapt. bar. *bell.* berb. *bism.* bov. bry. calad. calc. calc-p. camph. cann-s. canth. caps. *carb-an. carb-v. caus.* cham. chel. *chin.* chin-s. cic. cina. cinnb. clem. *cocc.* coloc. con. coral. creos. crotal. cupr. daph. diad. *dig. dros.* dulc. eugen. *euph.* euphr. eupi. *evon.* ferr. fluor-ac. gels. glon. *graph.* guai. hell. helon. *hepar.* hydr-ac. *hyos.* hyper. *ign.* indg. iod. ipec. k-ca. k-iod. kalm. lac-can. *lach.* lam. lact. laur. led. *lyc. mag-c. mag-m.* mag-s. mang. men. merc. merc-c. merc-i-r. merl. *mezer.* mosch. *mur-ac.* myric. nat-c. *nat-m.* nat-s. nicc.. *nitr. nit-ac.* nx-m. *nx-v.* olnd. ol-an. op. osm. par. *petr. phos.* ph-ac. pip-m. *plat.* plb. prun. psor. puls. ran-b. *ran-sc.* rheum. *rhod. rhus. ruta. sabad.* sabin. *samb.* sars. *seneg. sep.* serp. *sil. spig. spong.* squil. *stann. staph.* stront. *sulph.* sul-ac. tabac. tanac. *tarax.* tarent. tereb. thu. tong. valer. verat. verb. viol-tr. xanth. znc. zing.
— — compare with Bursting, Compressive, Drawing, Heaviness, etc.
— — **asunder**: acon. aloe. ant-c. arn. bar. *bell.* bov. *bry.* caps. chel. chin. cocc. euph. hell. ign. k-iod. lach. lil-t. lyc. merc. mezer. nat-m. nitr. nx-m. *nx-v.* par. prun. puls. ran-b. rhus. sabad. sabin. samb. *sep.* spig. stann. *staph.* stront. tarax. znc.
— — **band**, as if by a: clem. glon.
— — **burning**: aloe. alum. lact. mang. sep. sul-ac. tarax.
— — — from within out: nx-m.
— — **changeable**: gins. *ign.*
— — — from place to place: bell.
— — **congestion** as from: apis. chin. merl. nx-m. rhus-v.
— — **constricting**: graph.
— — **cramp-like**: ars. colch. ph-ac. plat. ran-sc. znc.
— — **deepseated**: agar. *arg-n. bell.* caus. cic. con. gins. indg. lach. nat-m. nat-s.
— — **desire** for pressure: chin.
— — — against wall: rham-c.
— — — upon floor: sang.
— — **digging**: bry. clem.
— — **downward**: ambr. ant-t. asar. cic. *cina.* con. *cupr.* hura. laur. mang. men. merc. merc-i-fl. mur-ac. nit-ac. nx-v. *phos.* ph-ac. plat. rhus. senna. spig. spong. *sulph.* verat.
— — **drawing**: agar. ang. ant-c. ant-t. arg. ars. asaf. aur. carb-v. caus. coff. hell. hepar. ign. iod. k-ca. mosch. nat-c. nit-ac. olnd. ran-b. ran-sc. *rhod.* rhus. sabad. sars. spig. stann. staph. tarax. thu.
— — **dull**: aloe. apis. canth. cimic. con. ferr. hydr-ac. lith. op. phys.
— — **extending** to nape: sabin.
— — **forward**: bry. nit-ac. sil. sulph.
— — **gnawing**: *ran-sc.*
— — **hat**, as from tight: sulph.
— — **heavy**, see Weight.
— — **inward**: *anac.* asar. cham. cocc. coff. dulc. hell. ign. nit-ac. olnd. *plat. ran-sc.* sabad. spig. staph. zing.
— — — as if by sharp corners: cham.
— — **moving** to and fro: bell. phos.
— — **outward**: *acon.* anac. arn. ars. *asaf.* asar. *bell.* berb. bry. camph. chel. cob. con. *coral.* creos. dros. dulc. euph. ferr. fluor-ac. hell. hepar. hyper. ign. laur. lil-t. lyc. men. merc. nx-m. *olnd.* par. ph-ac. phyt. pic-ac. prun. psor. ptel. ran-sc. rhod. sabad. sabin. samb. *sep.* sil. spig. spong. sulph. tarax. znc.
— — — as if by a sharp instrument: prun.

— — — as if contents would be forced out: lil-t.
— — **painful**: creos. lyc. ol-an.
— — **paroxysmal**: carb-v. cham.
— — **right** to left, from: eup-pur.
— — **spots**, in: *bell.* cic. dig. dulc. glon. meph. nx-v. psor. thu. znc.
— — **stoop**, must: cann-i.
— — **throbbing**: bry.
— — **upward**: fluor-ac. guai. meph. ph-ac. spig.
— — **wedge-like**, body icy cold: lachn.
— — — see also under Nail, Plug, etc.
— — — like fingers pressed in: meph.
— — — like a button: thu.
— — — peg on small spots: dulc.
— — — thumb: acon. nit-ac.
— — **weight** or stone, like: ars. *bism.* cann-s. carb-v. *cina.* laur. led. men. merc-i-r. nit-ac. *nx-v.* plat. *rhus.* sars. sulph. thu.
— **brain**, pressure forward of the: asar. bell. bry. ipec. k-ca.
— — against skull: mezer. rhod. rhus-v.
— — — sharp corners: sabad.
— — **inward**: asar. glon.
— — **on the**: ars. *cann-i.* glon. ign. manc. men. ruta. sep.
— — **outward** of the: agar. *bell. bry.* glon. hepar. hydr. indg. laur. lil-t. nat-m. phys. stann.
— **forehead**: *acon.* æsc h. æth. agar. agn. aloe. alum. ambr. am-c. am-m. anac. ang. ant-t. apis. arg-n. *arn.* ars. asaf. asar. aur. bapt. bar. *bell.* berb. *bism.* borax. bov. brom. *bry.* calad. calc. calc-ac. camph. cann-i. cann-s. canth. caps. carb-an. cast. caus. cham. *chel.* chin. cic. cimic. cina. clem. cocc. coff. *coloc.* con. cop. coral. croc. cupr. cyc. dig. dros. dulc. elaps. euph. euphr. eupi. ferr. gels. gent-l. gins. glon. gran. graph. grat. guai. hell. hydr-ac. *hyos.* hura. ign. indg. iod. ipec. jatr. k-bi. k-ca. kalm. lach. lachn. lact. laur. led. lil-t. lyc. mag-c. *mag-m.* manc. mang. marum. men. merc. mezer. mosch. mur-ac. nat-c. nat-m. *nat-s.* nit-ac. nitr. nx-m. *nx-v. ol-an.* olnd. op. oxal-ac. *par.* petr. phos. ph-ac. plat. plb. plect. prun. psor. puls. ran-b. raph. rheum. *rhod.* rhus. ruta. sabad. sabin. samb. sars. seneg. sep. sil. *spig.* spong. squil. *stann.* staph. stram. stront. *sulph.* sul-ac. tarax. tarent. thea. thu. tilia. tong. ustil. *valer.* verat. *verb.* vinc. viol-tr. *znc.*
— — **eyes**, over: acon. agar. aloe. am-c. ang. apis. arg-n. arn. asaf. bar. borax. bov. brom. bry. cann-i. carb-v. chel. chin. cist. con. creos. euph. eupi. evon. fluor-ac. glon. grat. gymn. hæmat. ign. indg. iod. k-ca. kalm. lach. lil-t. lith. mag-c. merc. merc-c. merc-i-r. merl. morph. nat-m. nat-p. nat-s. nitr. nx-m. op. pæon. petr. phyt. pic-ac. plan. plect. puls. *rhus.* ruta. sabad. sant. seneg. sep. sil. sol-t-æ. staph. stront. tabac. thu. znc. zing.
— — — as if eyes would be forced out: cocc. gymn. ign. lachn. nat-m. phos. sabin. seneg. sep. tarent.
— — — right eye, upward and inward: bism.
— — — pressive pain above left eye, followed by a dull, pressive pain in occipital protuberances, thence spreading over whole body; on quick motion and after eating pain so severe that it seemed a distinct pulsation in head: *bry.*
— — — pressure so severe, when rising, could only half open eyes, could not look up: stram.
— — **nose**, above: *æsc-h.* ambr. am-m. ant-t. asar. *bapt.* bar. bism. cann-s. carb-v. chin. coloc. euphr. glon. helon. hydr. ign. iod. manc. mezer. nitr. ph-ac. raph. sil. *stict.* tilia. viol-tr. *znc.* zing.
— — **right side** of forehead: anac. arn. asaf. bell. caus. chel. chin. guai. hell. ign. k-ca. marum. men. merc. mezer. mosch. nitr. nx-v. par. phos. plat. rhus. ruta. sabad. sabin. sars. spong. staph. stront. sul-ac. thu. valer. verb. viol-tr. znc.
— — — — then in left: colch.
— — **left side** of forehead: agn. ambr. ant-t. arg. asaf. aur. camph. cann-s. caus. chel. cic. cina. coloc. euph. ign. iod. mag-c. marum. merc. mur-ac. nat-c. nat-m. nitr. nx-m. nx-v. ph-ac. plat. ran-b. rhod. sabad. sabin. sars. seneg. spong. squil. stront. sul-ac. thu. znc.
— — **asunder**. See Outward.
— — **backward**: dios. spong. tabac.
— — **ball**, as from a: bell. con. mag-s.
— — **between** forehead and brain, sensation as if something pushed in: caus.

— — **crazy**, with fear of becoming: ambr.
— — **downward**: ambr. ant-t. asar. bell. bry. cina. cocc. glon. mur-ac. rhus.
— — **extending** downward: *bry.* chin-s. merc.
— — — to eyes: asaf. bell. laur. nitr. nx-m. op.
— — — inward: agar. laur.
— — — to nose: agar. aloe. am-m. *calc.* ph-ac.
— — — to occiput: bry. cann-s. chel.
— — — outward: aloe. bar-ac. lact. prun. psor. *stann.* staph.
— — — to vertex: creos. glon. puls.
— — **finger**, as from a: ol-an. stront.
— — **hat**, as from a tight: *almn.*
— — **forward**: hydr. laur. mag-s. nx-m. rhus.
— — **inward**: agar. alum. ant-c. bapt. bell. brom. calc. cocc. croc. ferr. hell. k-ca. laur. mosch. nx-v. olnd. plat. ran-sc. rhod. rhus. spig. stann. staph. sulph. verb. znc.
— — — as though eyes would be drawn into head: hepar.
— — **outward**: acon. aloe. alum. *am-c.* anac. ang. arg-n. asaf. bar. bell. *berb.* brom. *bry.* calc. camph. cann-i. cann-s. canth. caps. cast. chel. chin. cic. cina. coloc. con. *coral.* creos. cupr. dros. dulc. graph. hell. hepar. hydroph. ipec. k-ca. lil-t. mag-m. mag-s. mang. marum. men. merc. mezer. mur-ac. nat-m. nx-m. *olnd.* op. phos. ph-ac. plat. prun. psor. puls. ran-b. rhod. rhus. sabad. senec. sep. sil. *spig.* spong. stann. staph. stront. sulph. tarax. thea. thu. verb. viol-tr.
— — — as though brain would come out: acon. all-c. am-c. ang. arn. *bell.* brom. *bry.* canth. carb-v. caus. chel. colch. coloc. creos. k-ca. mag-m. mag-s. mang. mezer. nat-c. nx-v. phos. plat. puls. ratan. rhod. sabad. sep. *sil.* spig. spong. stann. staph. stront. sul-ac. thu. verb.
— — **upward**: glon. valer.
— — **weight**, or stone, like: acon. am-m. ant-t. asar. bell. cham. dig. rhus. sep. spig. tarax.
— **occiput**: acon. agar. aloe. alum. ambr. anac. ant-t. apis. *arg.* asaf. asar. aur. bapt. *bell.* berb. *bism.* bov. bry. cact. calc. calc-ph. camph. cann-i. cann-s. canth. carb-an. carb-v. caus. cedr. chel. chin. cic. cinnb. cocc. *colch.* coloc. con. croc. cupr. dig. dulc. euph. fluor-ac. gent-l. gins. glon. gran. graph. grat. guai. hell. hydr-ac. ign. iod. ipec. jatr. k-bi. k-ca. lach. lact. laur. lobel. lyc. mag-m. mang. marum. men. meph. merc. mezer. nat-c. nat-m. nat-s. nitr. nit-ac. nx-m. nx-v. ol-an. par. *petr.* phos. ph-ac. pip-m. plb. puls. ran-b. rhod. rhus. ruta. sabad. sabin. sars. selen. seneg. sep. *sil.* spig. spong. squil. stann. staph. stront. sulph. sul-ac. tabac. *tarax.* thu. tilia. valer. verb. znc. zing.
— — **asunder**: aloe. staph.
— — **deep-seated**: cast.
— — **downward**: card-b. hydr-ac. merl.
— — **extending** forward: ant-t. hydr-ac. mang. ol-an. ph-ac. sabad.
— — **forward**: chel. mang. nx-v. ol-an. ph-ac. plb. sabad.
— — **intermittent**: phel.
— — **inward**: bar. calc. ign. meph. olnd. oxal-ac. ph-ac. sep. spig.
— — — between vertex and occiput: oxal-ac.
— — **outward**: berb. bry. carb-v. chin. *gels.* mezer. ph-ac. prun. stann. staph. stront. tilia.
— — **plug**, nail, etc., as from: bov. canth. con. rhod. tarent.
— — in **spots**: glon. olnd. ol-an.
— — as with thumb: acon.
— — **weight** or stone: anac. asar. cann-s. carb-v. caus. cina. cocc. cupr. graph. hell. laur. led. men. nitr. nx-v. ph-ac. plat. sulph.
— **side** of: acon. agar. (r), agn. (r), alum. am-m. anac. ang. arg-n. (r), arn. arum-t. *asaf.* asar. (r), bar. (r), bell. bov. (l), bry. (r), cact. (r), calc. cann-s. *caps.* cedr. chel. chin. clem. coca. coloc. con. creos. crotal. (l), dig. dros. (r), euph. glon. grat. (r), hydr-ac. hell. (l), hepar. (r), ign. (r), iod. (l), k-bi. kalm. (r), laur. lil-t. (r), mang. men. mezer. (r), mur-ac. (l), nat-m. nat-s. nitr. nx-m. olnd. (r), pæon. phos. ph-ac. (l), pip-m. rheum. (r), rhus. (l), sabad. (r), sabin. samb. sars. (l), sep. *spig.* (l), spong. (r), stann. staph. stront. (l), sulph. (l), tabac. (r), thu. verat. verb. viol-tr. znc. (r).

— — **asunder**: *spig.* (r).
— — **brain**, as if, against the bone: mezer. (r).
— — — as if something were lying on the: grat. (r).
— — **board**, like a heavy: eugen.
— — **downward**; calc. con.
— — **extending** to eyes: *hydrph.*
— — — to forehead: hydr-ac.
— — — toward temple: nitr.
— — **hoop**, like a: therid.
— — **internal**, when leaning head against wall: cann-s.
— — **inward**: asaf. calc. croc. dulc. k-ca. hydroph. nat-s. olnd. plat.
— — **outward**: *asaf.* (l), asar. bell. (l), cina. (r), creos. dros. (r), merc. ph-ac. (r), spig. (r), spong. (r), stann. (r), verb. (r), viol-tr. (r).
— — **stupefying**, as with blunt instrument: olnd. ruta.
— — **sudden**, as from blunt tool, pressed in: asaf.
— — **tool**, as from a blunt: asaf. dulc. hepar. olnd. ruta.
— **temple**: acon. agar. agn. (r), aloe. alum. *anac.* ang. ant-t. apis. arg. arn. ars. *asaf.* asar. aur. bell. berb. *bism.* bov. brom. (l), *bry.* calad. (r), calc. camph. cann-i. *cann-s.* canth. caps. carb-an. carb-v. cast. caus. cedr. (r), *cham.* chel. chin. cina. cinnb. coca. (l), clem. coff. colch. (r), *coloc.* con. cupr. *cyc.* dig. dios. dros. dulc. euph. ferr. fluor-ac. (l), gent. gins. glon. gran. (l), graph. (l), guai. hell. hepar. hipp. hura. (l), hydr-ac. hyos. hyper. ign. indm. (r), iod. ipec. jatr. k-ca. kalm. lach. lachn. lac-can. laur. led. lith. lobel. lyc. mang. marum. men. merc. merl. mezer. mosch. (r), nat-c. nat-m. nat-s. nitr. (r), nx-m. olnd. ol-an. op. par. petr. phos. ph-ac. plat. prun. psor. ran-b. ran-sc. rheum. (r), *rhod.* rhus. sabad. sabin. samb. sars. seneg. sep. sil. spig. spong. *stann.* stront. sulph. sul-ac. tabac. tarax. taxus. (l), thu. verat. verb. viol-tr. znc.
— — **eyes**, drawing in eyes as if strabismus would follow, pressing in temples with: podo.
— — **extending** across forehead: bry. seneg. sol-n.
— — — to neck: chel.
— — — to occiput: lil-t. (l), sabad.
— — — to vertex: chel.
— — **forward**: verb.
— — **inward**: acon. alum. *anac.* ant-c. ant-t. asaf. asar. bell. calc. cocc. dulc. fluor-ac. hell. jatr. k-ca. lith. mezer. nat-c. nit-ac. ol-an. ph-ac. plat. *ran-sc.* rhod. sabad. sabin. seneg. sol-n. *spig.* stann. staph. sul-ac. thu. valer. znc.
— — **outward**: acon. aloe. anac. asaf. (l), berb. bism. bry. calc. canth. carb-v. (l), cast. caus. chin. creos. dros. (r), fluor-ac. glon. ign. indg. ipec. k-ca. (r), lach. lact. lil-t. lobel. marum. *mezer.* (l), mur-ac. (r), nat-c. (r), nat-m. nx-m. (r), op. par. ph-ac. phys. phyt. prun. ran-sc. rhod. *sabad.* (r), sabin. (l), samb. senec. *spig.* spong. (r), stann. stront. sulph. valer. verb. (l), viol-tr.
— — — with heat of face and flickering before eyes: aloe.
— — **paroxysmal**: ptel.
— — **spots**, in: helon. oxal-ac.
— — **upward**: rhus.
— — **weight**, plug, or stone, like: ambro. ant-t. arn. asaf. cocc. dulc. hell. rhus. sep.
— — — as from strong pressure with thumb: cham.
— **vertex**: *acon.* æsc-h. agar. agn. aloe. alum. ambr. am-c. anac. ang. ant-t. apis. arg-n. arn. asaf. asar. aur. bar. bell. benz-ac. bov. cact. calc. *cann-s.* carb-v. cast. caus. cedr. cham. chel. chin. cina. clem. cocc. cocc-c. colch. coloc. con. creos. croc. cupr. cyc. dig. dulc. eup-per. ferr. ferr-p. gels. glon. graph. hell. helon. hepar. hipp. hydr-ac. hydroph. hyos. hyper. ign. iod. k-bi. k-ca. *k-iod.* lach. lac-can. laur. led. lil-t. lith. lyc. mag-m. manc. men. mezer. mosch. nat-c. nat-m. nicc. nitr. nx-m. nx-v. olnd. ol-an. op. pallad. petr. phos. ph-ac. *phys. phyt.* plat. ran-b. ran-sc. rheum. rhod. rumx. sabad. sabin. sars. sep. *sil.* spig. spong. stann. staph. stram. *sulph.* sul-ac. tabac. thu. tong. valer. *verat.* verb. viol-tr. znc.
— — **asunder**: carb-an. nx-v. ran-b.
— — **cloth**, as if brain were enveloped in a, which would deprive of senses: cyc.
— — **downward**: ambr. cina. croton. cupr. hydr. nx-m.
— — **extending** to eye: calc.

— — — to forehead: *cham.* hydr-ac. nat-m.
— — — to shoulders: gels.
— — — to spine, no pain: benz-ac.
— — **finger**, as from: nit-ac.
— — **hard** body, as from: *ign.* nx-v. thu.
— — **inward**: anac. asar. caus. dulc. ferr. glon. hell. nit-ac. nx-m. oxal-ac. ph-ac. plat. ran-sc. sil. staph. sulph.
— — **small spot**: spig.
— — **on** verte. : acon. alum. anac. ant-t. bov. brom. canth. carb-v. cham. chel. chin-s. croc. dros. euphr. ferr. hell. hydroph. k-bi. mosch. myric. nat-c. nat-m. nicc. nit-ac. ol-an. phel. sars. sep. stann. staph. sol-n. *sulph.* tabac. thu. *verat.*
— — **outward**: calc-p. cham. op. ph-ac. phys. spig.
— — **slowly** increasing and decreasing: sars.
— — **upward**: helon. ferr.
— — — and outward, as if there were no room for brain: cimic. glon.

**Conditions** of Pressure.
— **morning**: *acon.* alum. ambr. arg-n. asaf. benz-ac. bov. cann-s. caus. cedr. *cham.* chin. cimic. croc. cyc. glon. k-bi. lach. lyc. mezer. myric. nat-c. nat-m. nat-s. nicc. nitr. nx-v. pæon. *petr.* phos. ph-ac. pip-m. puls. rhus. sil. sulph. tong. thu.
— — 3 to 4 A. M.: thu.
— — on waking: agar. anac. arg-n. cocc-c. ferr. gels. hepar. mezer. ol-an. ph-ac. znc.
— — on rising: cinnb. graph. oxal-ac. psor. sabin. squil. *sulph.*
— **noon**: agar. manc. sil. *sulph.* znc.
— — relieved toward: bry.
— **afternoon**: alum. ang. cann-i. carb-v. cham. coloc. hell. nat-c. op. ph-ac. senec. sep. stram.
— **evening**: acon. alum. ambr. *anac.* arg-n. ars. cast. cham. chel. colch. coloc. dig. dios. ferr. fluor-ac. hell. hydr-ac. *hydroph.* hyper. kalm. mag-m. mag-s. nat-m. nat-s. ol-an. rhod. rhus. staph. SULPH. thu. *znc.*
— — after sunset: nat-s.
— **night**: hepar. lyc. nx-v. sulph.
— — on awaking: canth.
— **air**, in open: agar. caus. ferr. glon. hepar. laur. nx-v. rhus.
— — amel. in open: alum. cinnb. hell. hydr-ac. mag-m. phos. sabin. seneg.
— **arms**, on moving: rhus.
— **ascending**, on: arn. men. ph-ac.
— **bed**, when in: kalm. nat-s. ol-an. pip-m. ran-b. rhus. sulph.
— **bending** backward, amel.: *bell.* ph-ac.
— — — aggr.: mang.
— **breakfast**, after: chel. hydr.
— — — amel.: *psor.*
— **boots**, when drawing on the: apis.
— **chill**, during: sep.
— **coffee**, after strong: arum-t. nat-s.
— **company**, when in: mag-c.
— **constipation**, during: jatr.
— **coughing**, when: acon. alum. *bry.* chel. con. creos. hepar. nit-ac. phos. ruta. sars. sep. spig.
— **covered**, pressing with distress, when: led.
— **darkness**, in: sil.
— **descending**, on: men.
— **diarrhœa**, during: rhus.
— — pressing pain in forehead, at its height vomiting and diarrhœa: æth.
— **dinner**, during: pallad.
— — after: alum. calc. carb-an. ol-an. ruta. seneg. tabac. thu. *znc.*
— **drinking** aggr.: cocc.
— **eating**, after: alum. calc. carb-an. carb-v. chel. clem. *cocc.* con. graph. hydr. hyos. lyc. nat-s. ol-an. pip-m. ran-b. ruta. tabac. thu. *znc.*
— — amel.: psor.
— **excitement**, after: chin-s.
— **exertion**, aggr.: bell.
— **eyes**, on moving: bell. *chel.* hepar.
— — turning toward affected side, aggr.: con.
— — opening, after sleep: rhus.
— — using: gent. hell.
— **fever**, during: sep.
— — with hay-fever: sabad.
— **hat**, worse from pressure of: calc-p.
— **head**, on bending forward: *bell.* ferr-p. nat-m. ph-ac.
— — lying with head high, amel.: spig.
— — moving, when walking: ars.
— — resting against wall (internal pressure), when: cann-s.
— — shaking, on: chin. glon.
— **heated**, when: am-c.
— **hiccough**, during: *bry.*
— **house**, amel. in: cocc.
— **looking**, steadily, aggr. when: hell.

— **lying** down: glon. pip-m. nat-s. TARAX.
— — on side, when: bar.
— — amel.: bell. nit-ac.
— — see also Bed.
— **menses**, before: petr. sep.
— — during: acon. bell. *bry.* cast. cimic. *creos.* cyc. eupi. nat-m. nx-v. *sep.* stann. sulph.
— — — on vertex: calc. cast. nx-v.
— — after: ustil.
— **mental** exertion from: arg-n. arn. asar. cact. carb-an. cham. cocc. colch. dig. helon. lyc. mag-c. nat-s. ph-ac. pic-ac. sep. *sulph.*
— — **reading**, while: bell. cocc. helon. lyc.
— — when attention is concentrated: helon.
— **motion**, aggr.: agn. arg-n. bism. calc. cupr. dulc. glon. hell. phos. ph-ac. pic-ac. spig. thu.
— — to one side, on: thu.
— — amel.: *agar.* ferr. pip-m.
— **nausea**, during: asar.
— **neck**, on moving the: glon. nat-s.
— **noise**, aggr.: nit-ac. ph-ac. spig.
— **nose-bleed**, before: *carb-an.*
— **pressure** of hat aggr.: calc-p.
— — amel.: arg-n. asaf. chin. dios. *men.* nat-s. op. stann.
— **rest**, amel.: arg-n. cocc. nat-s. pic-ac. rhod. spig.
— — in one position, aggr.: pip-m.
— **riding** in wagon aggr.: cocc.
— — amel.: nit-ac.
— **rising**, on: asaf. bell. cinnb. glon. mag-s. nit-ac. *spig.*
— — after amel.: ran-b. stann.
— **room** (house), in: laur. mag-c. nat-m. nat-s. phos.
— — hot, in: cocc-c.
— — amel. in: hepar.
— **rubbing**, amel.: op. ph-ac.
— **sitting**, when: agar. alum. *bry.* benz-ac. fluor-ac.
— — up in bed, amel.: canth.
— — amel.: bell. pic-ac.
— — erect, when: mang.
— **sleep**, after, amel.: thu.
— — aggr.: cocc.
— — after a siesta: calad.
— **smoking**, when: calad.
— **sneezing**, after: apis. cina.
— **standing**, on: alum. selen.
— — up, amel. on.: plb.
— **step**, every, in open air, at: glon.
— **stool**, before: merc.
— — during: coloc. gran. merc.
— — after: lyc. sil. spig.
— **stooping**, on: *bell. bry.* calc. canth. carb-v. cham. hepar. fluor-ac. lyc. mag-m. marum. merc. merc-c. par. petr. phos. *spig.* stann. zing.
— — must bend forward: cann-i. ign.
— — amel.: caus. mang.
— **sun**, when in; passes off in shade: brom.
— **supper**, after: carb-v. ran-b.
— **sweat**, amel.: thu.
— **talking** loudly, when: spig.
— **tea**, after warm, amel.: glon.
— **thinking** of it, aggr.: cham. cocc. dig. *helon.*
— **thirst**, with: iod.
— **vomiting** and diarrhœa, with: æth.
— **waking**, on: arg-n. bry. calad. nat-m. rhus. thu. *verat.*
— **walking**, when: arn. bell. *bry.* calc. caus. clem. cocc. nat-m.
— — in open air: agar. *bell.* glon. ferr. staph. thu.
— — — — — after: *bell.* znc.
— **washing**, after, amel.: ferr. *psor.*
— — cold bath, amel.: euphr.
— **writing**, when: k-ca. nat-c.

**Pricking**, prickling: am-m. apis. arg-n. aur. cham. chin-s. hydr-ac. lachn. ph-ac. sabad. thu. verb. viol-od.
— — as from needles: con. eugen. rhus. thu.
— **forehead**: apis. aur. chin-s. ferr. lil-t. sabad. sep. thu. verat. *viol-od.*
— — intermittent: verb.
— — in spots: apis.
— — like needles: agar. all-c. am-c. asaf. caul. hepar. k-ca. mang. nat-m. sep.
— — pricking, stinging above root of nose: k-bi.
— **temple**: ailan. (1), ant-c. apis. cupr. cocc. euphr. rhus-r. tarent. tarax. thu. verb.
— — as with needles: nicc. znc.
— evening: lachn.

**Pulled**, sensation as if hair were: *æth.* alum. *arg-n.* arn. canth. carb-an. chin. laur. mag-c. mag-m. mur-ac. nitr. phos. psor. rhus. stann.
— out: ars. bell.
— vertex, from the: *acon.* ferr. indg. mag-m. nitr.

**Pulling** like, pain: canth. *lach.* petr.

— — extending into teeth: staph.

**Pulsation**, beating, throbbing, etc.: *acon.* æth. ailan. aloe. alum. anac. ant-t. apis. *arn.* *ars.* asaf. asar. aur. *bell.* *borax.* bov. *bry.* cact. cadm. calc. calc-p. *camph.* *cann-i.* cann-s. *caps.* carb-an. *carb-v.* *cast.* *caus.* cedr. *cham.* chel. chin. chin-s. cimic. cinnb. clem. cob. cocc. coff. colch. *creos.* croc. crotal. croton. cupr. cyc. daph. dig. dros. eugen. eup-per. euphr. eupi. *ferr.* gels. *glon.* graph. grat. guai. hell. hepar. hipp. hyos. *ign.* indg. indm. iod. ipec. k-bi. k-ca. k-iod. lach. lact. lam. *laur.* led. lith. *lyc.* mag-c. mag-m. mag-s. manc. mang. merc. mezer. mill. mur-ac. myric. nat-c. nat-m. nicc. nit-ac. nitr. nx-m. nx-v. olnd. ol-an. op. par. *petr.* phel. phos. ph-ac. plb. psor. *puls.* *rheum.* rhod. rhus. ruta. sabad. sabin. sang. *sars.* secale. seneg. *sep.* *sil.* sol-n. *spong.* squil. stann. stram. *sulph.* tabac. therid. thu. tilia. *tong.* verat. *verat-v.* znc.

— — compare with Bursting, Congestion, Hammering, etc.

— — **abdomen**, rising from: rheum.

— — **aching**: acon. æth. ang. ars. aur-m. bell. bry. calc. calc-p. calc-s. camph. canth. caps. caus. cham. chel. clem. coll. cupr. cupr-s. eugen. ferr. ferr-m. glon. ham. hepar. hydrs. hyper. ign. indg. k-bi. manc. meli. nat-m. nat-p. nat-s. nicc. nit-ac. nx-m. petr. ph-ac. psor. ptel. rheum. rhod. rhus-r. sang. sep. sil. sulph. tarent. upa. verat. xanth.

— — **alternating** between head and chest: bell.

— — **burning**, throbbing: apis. coff. *rhus.*

— — **drawing**: ars.

— — **extends** to neck or chest: nat-m.

— — **gnawing**: par.

— — **hammers**, as if from little, awakens every morning: *nat-m.*

— — **here and there**: acon. æth. indg.

— — **intermittent**: ferr-m. verat.

— — **jerking**: bry. ign. phos.

— — **painless**, with fear of going to sleep: nx-m.

— — **paroxysmal**: caus. glon.

— — **pressing**, throbbing, in spots, worse in left supraorbital ridge: nx-m.

— — **shooting**: æth. bell. ferr. lam. nx-v.

— — — as from an internal abscess: acon.

— — **stinging**: puls.

— — **tearing**: carb-an. mag-m. nat-c. sil. spong. znc.

— — **teeth**, extending to: mezer.

— — **transient**, in one half of brain: cham.

— — **ulcerative**: bov. cast. mang.

— **brain**, seems to be in: *bell.* cyc. dig. glon. hyos. op. lyc. rhus.

— — **deep-seated**: cic.

— — **beating** of, against skull: *ars.* *bell.* daph. hydr-ac. psor. *sulph.*

— — — as of waves: *chin.* dig.

— — — hammers, like little: *nat-m.* psor.

— — — throbbing pain in middle of brain, every morning, lasts all day: calc.

— — **shooting**, ending in: *bell.*

— — **transient**, in one half of: cham.

— **forehead**: acon. æth. aloe. alum. *am-c.* am-m. ang. ant-t. apoc. arg. ars. asaf. asar. aur. bar. *bell.* borax. bry. calc. calc-p. camph. *cann-i.* cann-s. canth. *caps.* carb-v. cast. caus. cic. cinnb. clem. cocc. corn. creos. croc. dig. dulc. euph. eupi. gamb. *glon.* graph. grat. hell. ign. iod. iris. k-ca. kalm. laur. lyc. mag-c. mag-m. mag-s. merc. merc-i-fl. mezer. nat-c. nat-m. nat-p. nit-ac. nx-m. olnd. op. oxal-ac. par. petr. phos. puls. ran-b. rheum. rhod. rhus. ruta. sabad. sars. seneg. sep. sil. spig. spong. stram. therid. thu. verb. vibur. znc.

— — **aching**: bell.

— — — in spots: nx-m.

— — — — pulsating in forehead, with aching in occiput: carb-v.

— — **blows** in, pulsation as from strong: acon.

— — **extending** to occiput: bry.

— — **eyes**, over: gymn. k-bi. lac-can. lach. nx-m. spig. therid. vibur.

— — **nose**, above: ars. mezer.

— — **side**, left of forehead: acon. cimic. cocc. k-ca. nx-m. par. spig. verat.

— — — one sided: aur.

— — — on frontal protuberance: arg-n.

— **occiput**: æth. agar. ailan. aloe. alum. am-c. asar. bar. bell. berb.

borax. *bry.* calc. camph. cann-s. carb-an. carb-v. caus. con. dros. *eup-per.* ferr. *gels.* glon. hepar. hura. ign. indg. k-ca. lac-can. laur. lyc. mag-m. nat-m. nit-ac. nitr. *petr.* phos. phys. psor. puls. ran-b. rhus. ruta. *sep.* spig.
— — from occiput to forehead : carb-v. sil. *spig.* (1).
— — gradually extending to sides and forehead, worse on stooping or moving: ferr.
— — hammer, like beats of, in cerebellum : camph.
— **side** of: æth. (r), agar. (r), alum. (r), am-c. (1), ant-t. arg-n. ars. aur. (r), bar. bell. bov. (r), bry. calc. (1), canth. (r), cham. coca. (r), con. (r), croc. (1), dirc. (1), eup-per. glon. graph. (r), hura. (1), k-ca. (r), k-iod. kalm. (1), laur. mag-c. (r), mag-m. (1), mag-s. (r), nat-c. (1), nit-ac. (1), ol-an. petr. phos. (1), rhod. (r), rhus. (r), sep. spong. sul-ac. (r), tong. verat. znc.
— — deep-seated: sars. (r).
— — from left to right: nx-m.
— — from right to left: bov.
— **temples** : æth. (1), all-s. alum. (r), am-m. (r), ant-c. (1), ars. asaf. (1), bell. borax. bry. cact. cadm. calc. camph. caps. carb-v. cast. (r), cedr. chel. chin. cocc. coloc. daph. ferr. fluor-ac. (r), gins. *glon.* hell. (1), hepar. hyper. (r), hura. (1), k-ca. k-iod. lac-can. lach. lac-ac. laur. (r), nat-m. nat-s. nit-ac. nitr. phos. phys. plb. rhus. (1), sabad. sars. (r), spig. spong. (1), stann. stram. sulph. sul-i. tabac. thea. thu. verat. verat-v.
— — **aching** : alum. arn. bov. camph. cocc-c. coloc. glon.
— — **burning** : cinnb.
— — pressive, screwing, throbbing pain in one frontal protuberance, temple or into bones of face; at its height trembling of body; nausea, bilious vomit; lies senseless, eyes closed, shuns light and conversation: arg-n.
— **vertex** : æth. agar. alum. ars. bry. calc. cann-i. caus. *chel.* cinnb. cocc. creos. corn. ferr. glon. grat. ham. *hyper.* hydrph. k-ca. lach. lyc. manc. *merc-i-r.* nat-c. nx-v. phel. phos. sars. sep. *sil.* *stram.* sulph. thea. tong.
— — **aching** : bell. canth. glon. sulph. tong. verat.
— — **ascending** from base of skull: glon.
— — **bruised** : caus.
— — like beating of waves : znc.
— — as if all would come out there: caus.
— — **pressing** : grat. nx-v.

**Conditions** of Pulsation.
— **morning** : aur. bov. canth. cedr. cob. gamb. glon. graph. grat. indg. lact. lyc. nat-c. nicc. plb. sars. sep. sil. spig. *sulph.*
— — every, lasts all day: calc.
— — increases till evening: eup-pur. sang. sep.
— — on awaking: *bry.* lach. nat-m. phos. ruta.
— — on rising : asar. caus. nat-m.
— — comes on gradually and goes off about breakfast: nit-ac. (1).
— **afternoon** : æth. alum. cast. caus. coca. glon. graph. grat. hura. indm. lyc. mag-s. *merc-i-r.* nat-m. phel. phys. sil.
— **evening** : acon. am-m. bov. calc. canth. carb-v. cic. clem. cocc. con. cyc. fluor-ac. glon. indg. iris. k-iod. lac-ac. mag-s. nit-ac. oxal-ac. puls. ruta. stram.
— — on lying : carb-v. *lyc.*
— — worse in, until gets to sleep: cast.
— **night** : aloe. ars. *cact.* chel. glon. hura. lyc. sars. sil. *sulph.*
— — in bed before sleep : chel.
— — on waking : carb-v. sulph.
— — worse after 12 P. M. : ferr.
— — comes on during the, with nausea and vomiting : sil.
— **air**, worse in open : carb-an. cocc. eup-pur. iris.
— — better in : ant-t. k-bi. mang. phos.
— **ascending**, aggr. on : alum. glon. nat-p.
— — fast: glon.
— **bathing**, after : cast.
— — cold partially amel. : ars.
— **bed**, when in : chel. con. cyc. graph. sep.
— **bending** backward, aggr. when: aur. *lyc.*
— — — amel. : *bell.* *nat-m.* *sil.*
— — forward. See Stooping.
— **blood**, after loss of: chin.
— **breakfast**, amel. after: nat-m. nit-ac.

— **chewing**, when : phos.
— **chill**, during: cann-i. eup-per.
— — with chilliness : sil.
— **cold**, preceding a : lach.
— **debauch**, after a: carb-v.
— **coughing**, when : aur. dirc. ferr. hepar. hipp. ipec. iris. k-ca. led. lyc. nat-m. nit-ac. phos. ph-ac. seneg. sep. sil. spong. sulph.
— **dinner**, after: *am-c.* carb-an. k-bi. mag-c. nat-c. ol-an. plb. znc.
— **eating**, after: am-c. carb-v. clem. selen.
— — before and after: cocc.
— **drinking**, worse when: acon.
— **epistaxis**, after: borax.
— **exertion**, from: gins. glon.
— **eyes**, close involuntarily from sheer prostration: chin-s.
— — lies senseless, eyes closed: arg-n.
— — turning or closing, aggr.: sep.
— **fever**, during the: eup-per.
— **head**, on moving: *sulph.*
— — high, lying with, amel.: nat-m.
— — resting, amel.: k-bi.
— — raising it suddenly, aggr.: nat-p. squil.
— — shaking, aggr.: glon.
— **heat**, during: eup-per. glon. rhus.
— **house**, amel. in: cocc.
— **jar**, from any: *bell.* glon. *therid.*
— **laughing**, from: phos.
— **lying**, when: aloe. glon. naja. phos.
— — on back: sep.
— — on the part: *petr.*
— — on side, amel.: nat-m. sep.
— — must lie down: *bell.* sang.
— **menses**, before: *bell.* *borax.* crotal. gels. glon. lach. petr. sulph.
— — **during**: acon. bell. borax. bry. cact. *calc.* calc-p. *chin.* glon. ign. lach. mag-c. *nat-c.* puls. sang.
— — — painless throbbing: eupi.
— — **after**: calc-p. carb-an. ferr. glon.
— — suppressed, when: *puls.*
— — compare with Congestion.
— **mental** exertion, from: nat-m. psor. puls. *raph.* sil. vibur.
— **motion**, aggr.: acon. apis. ars. *bell.* calc-p. cimic. colch. dirc. ferr. *glon.* grat. *iod.* *lach.* *nat-m.* *sep.* sulph.
— — moderate, amel.: iris. vibur.
— — sudden, from: ferr.
— **periodic**: ferr.
— **perspiration**, amel.: nat-m.
— **pressure**, amel.: æth. am-c. bell. ferr. glon. nat-m. nitr.
— — with hands amel. temporarily: apis. guai.
— — upon forehead causes beating: mag-m.
— **pulse**, at each beat of: camph. cimic. glon.
— **reading**, when sitting: lyc.
— **respiration**, during difficult: *carb-v.* glon.
— **rest**, when at: aloe. lact.
— — amel.: eupi. sep. stram.
— **riding**, when, aggr.: cocc. *glon.* grat.
— **rising**, aggr. on: chin-s. dirc. glon. phos.
— — amel.: nat-c.
— — from stooping, on: mag-m.
— — up in bed, when: ars.
— **room**, on entering: æth. mag-m. mang. tong.
— — in dark, amel.: sep.
— — in warm: am-c.
— **rubbing**, amel.: æth.
— **sitting**, when: am-m. cast. indg. lyc. mag-m. ol-an.
— **sleep**, amel.: sang.
— **standing**, when: cast. plb.
— — amel.: camph.
— **stool**, when straining at: ign.
— **stooping**, by: apis. asar. colch. ferr. glon. hydr-ac. k-bi. lach. laur. nat-c. nat-m. phos. puls. sul-i.
— **stretching** limbs out, on: phos.
— **sunlight**, aggr. in: acon.
— **talking**, aggr.: acon. sil.
— **tea**, warm, amel.: glon.
— **thinking** of it, aggr. when not: ant-c.
— **turning** around, aggr.: glon.
— **vertigo**, during: glon. secale.
— **waking**, on: carb-v. cinnb. lach. nat-m. phos. ruta. sulph.
— **walking**, when: *bell.* calc. *glon.* k-bi. nat-s. nx-v. plb. sars. sil. sulph.
— — in open air: am-c. mag-s.
— — — — amel.: ars. eup-pur. guai.
— — quickly, when: ferr. nx-v.
— — stepping heavily, when: alum. phos.
— **washing**, cold, amel.: phos.
— — — amel. partially: ars.
— **wine**, after: oxal-ac.
— **wrapping** head up warmly amel.: [*sil.*
— **writing**, when: k-ca.
— **yawning**, after: calc.

**Pushed** forward, sensation as if: *canth.* grat. nit-ac. nx-m. nx-v. rhus.

— to forehead from occiput, sensation as if a load were: pallad.
**Pushing** sensation: croc. *nat-m.* phos. ph-ac.
— — amel. by pressure: *arg-n.*
— — compare with Pressure.
**Pustules** on forehead: anac. ars. carb-an. chel. eup-per. k-bro. mur-ac. rhod. sars.
**Quivering** sensation in: cann-s. lact.
— — on shaking head: xanth.
— — when running and walking: nx-v.
— — sensation as if brain were shaking when walking: rhodo.
**Raging**: æth. ant-t. aur. bov. caus. cinnb. clem. indg. led. mag-m. *merc.* mill. sabad.
— amel. in open air: ant-t.
— reading aggr.: clem.
**Raise**, difficult to, night: chel.
— frequently from pillow: stram.
— unable to: bell. chel. *puls.* nx-v.
— — — when lying on back: nx-v.
— — — after stooping: bell. rhus.
**Rash** on forehead: ailan. arn. indg. lil-t. rheum. marum.
— sticking, when cold: marum.
— see Pimples, etc.
**Redness** of forehead: calc. hura. laur. lil-t. merc-i-r. mezer. rhus-v. stram. vaccin. verat.
— — — spots: æsc-h. berb. caps. cyc. mosch. sulph. *tellur.*
**Reeling**: acon. bell. rhus.
— see Vertigo.
**Rending**, See Tearing.
**Restlessness**: ambr. bell. caus. ether. jabor. merc. merc-p. phos. pip-m. ruta. secale. sil.
— when too weak to move body, will roll head from side to side: *ars.*
**Rigid** feeling in: *caus.* phos. rheum.
**Ringing** in forehead: ailan.
— in head. See under Ears and Hearing.
**Rising** sensation in: glon. nat-c. nx-v. rhus.
— and sinking: bell. cob.
— when drinking beer: rhus.
— when walking rapidly: nx-v.
— from vertex to forehead, sensation of something: glon.
— sensation as if brain raised several times in succession: thu.
**Roaring**, rushing, in: acon. am-c. ars. *aur.* bar. calc. cann-i. cann-s. carb-v. caus. chin-s. cimic. cinnb. coff. creos. croc. ferr. graph. hepar. hura. hydr. hyper. k-bi. k-ca. lach. lact. lyc. mag-c. mag-m. mur-ac. nat-c. nat-m. nit-ac. nx-v. op. petr. phos. ph-ac. plat. *puls. rhus.* sars. sep. *sil. staph. sulph.* tabac. thu. *verat.* verb. znc.
— compare under Ears.
— **forehead**: verat.
— **temple**: ang. k-ca. stront.
— **vertex**: hyper. nat-m.
— like wind among trees: rhus-r.
**Conditions** of Roaring.
— **evening**: cinnb. hyper.
— — in bed: nat-m.
— **coition**, after: carb-v.
— **coryza**, during: sep
— **cough**, during: caus. hepar. mag-m.
— **eating**, after: cinnb.
— **hysterical** persons, in: aur.
— **menses**, during: brom. *creos.*
— **sitting**, while: phos.
— **stepping** heavily, when: *sil.*
— **stool**, after: znc.
— **sweat**, during: caus.
— **walking**, when: ferr.
**Rocking** of brain to and fro: acon. chin. rhus.
— see also Quivering, Waving, etc.
— rocks head from side to side to relieve the pain: k-iod.
**Rolling** in head: cup-ars. eugen. graph. hura. phys. sep.
— — sensation as if brain were rolled into small bulk: *cocc-c.*
— — sensation as if a lead ball rolled about in: hydrph.
— — — after study: cup-ars.
— — — during vertigo: sep.
— — — vomiting aggr.: eugen.
— **of the head**: merc. morph. nx-m. phos. stram.
— — — in paroxysms: merc.
— — — from side to side: hell. podo.
— — — — — — — when too weak to move the body: *ars.*
— — — when lying on pillow: merc.
— — — when sitting: *nx-m.*
**Rubbing**: camph. con. hyos. tarent.
— against something: tarent.
— inclination to rub forehead: glon.
— with the hand: verat.
**Semilateral** pains. See under Aching and special pains.
**Screwed** in. See under Compressive.
**Scraped** feeling, motion amel.; when lying the pain shifts to side laid on: ph-ac.

**Sensitiveness** of brain: *bell.* brom. bry. calc. carb-v. chin. con. croton. dros. gels. gent. hyos. iod. k-ca. *lach.* lact. led. lyc. mag-m. mezer. nit-ac. phos. raph. sil. *spig.* znc.

— to special causes, compare aggravations under General Conditions.

— **air**, to cold: brom. mezer.

— — to draught of: naja. tilia.

— **candle-light**: calc.

— **cold**, to: grat.

— **dinner**, after: gent.

— **hat**, to pressure of: croton. glon. sulph.

— **jar**, to the least: *bell.* glon. hepar. mang. nat-ars. nit-ac. ph-ac. *sil.* spig. *sulph. therid.* vibur.

— **motion** of head, to: gent. mang.

— **noise**, to: bar. calc. con. ph-ac. stram. *therid.*

— — shrill sounds: calc.

— — of wagons on street: nit-ac.

— — of reading: mag-m.

— — of speaking: con.

— — especially of male voices: bar.

— **stepping**, to: *bell.* calc-p. dros. gels. lyc. nat-m. nit-ac. raph. rhus. *spig.* stann. *sulph.*

— — when ascending: rhus.

— **touch**, to gentlest, after anger: mezer.

**Separated**, from body, were, night: daph.

— brain from skull, were: staph.

**Shaking** sensation in: acon. aloe. anac. ars. asar. aur. bar. bell. benz-ac. bufo. *calc.* cann-i. carb-v. caus. cic. cocc. crotal. elaps. eupi. fluor-ac. glon. hyos. k-ca. lact. led. lyc. mag-s. mezer. nx-m. nx-v. pallad. phos. ph-ac. plat. rhod. sep. sil. sol-n. *spig.* verat. viol-tr.

— compare with Looseness, Motion, Undulation, etc.

— of head, cannot bear: glon.

— by paroxysms: carb-v.

— against frontal bone: aur.

— **air**, aggr. in: aloe.

— **chills**, during: ars.

— **cold**, aggr.: nx-m.

— **cough**, during: chin. *lact.* mag-s.

— **eating**, worse after: nx-m.

— **heat**, except that of bed, amel.: nx-m.

— **motion** of head, on: cocc. lact. *spig.*

— — laying head down, on: aloe.

— **rest**, amel. at: nx-v.

— **room**, amel. in warm: nx-v.

— **speaking**, on: cocc.

— **stamping**, on: bar.

— **stepping** heavily, when: led. lyc. sil.

— — — striking foot against anything: sep.

— **stooping**, on: berb.

— **walking**, when: cic. cocc. led. lyc. mang. nx-v. sep. sil. spig. viol-tr.

— — in open air: caus. *nx-v.*

— **wrapping** up warmly, amel.: nx-v.

**Shattered** feeling in: æth. calc. hyos. k-ca. mang. nx-v. sil. stront. sul-ac. verat.

— — compare with Bruised.

— — on coughing: calc. lact. *rhus.*

— — on motion: mang. nx-v.

— — on stepping heavily: *sil.*

— — on waking in morning: sul-ac.

— — walking, when: nx-v.

— — — as if a ball were beating against the skull, on beginning to walk: plat.

— — with toothache: euph.

**Shocks**, blows, etc., in: acon. æth. all-c. alum. ars. asaf. bapt. bar. bell. benz-ac. bov. calc. camph. *cann-i.* carb-v. caus. clem. *croc.* crotal. ferr. glon. graph. hell. hydr-ac. hydrph. indg. ipec. k-ca. lach. laur. led. lobel. lyc. mag-s. *mang.* merc. mill. mur-ac. nat-c. *nat-m.* nat-s. nit-ac. nx-v. olnd. phos. ph-ac. plb. psor. puls. ran-b. raph. rhus. sabad. samb. sang. seneg. sep. *spig.* stann. sulph. *sul-ac.* tarent. thea. thu. valer. verat-v. znc.

— — compare with Jerks, Pulsation; see under Plug, Nail, etc.

— — electric, like: arn. lobel. nat-s.

— — here and there: znc.

— — from elbow to head: agar.

— — to cheek: puls.

— **forehead**: acon. am-c. ang. camph. caus. croc. hipp. glon. k-ca. laur. mag-s. nat-m. olnd. plat. rhus. sang. seneg. spig. stann. sul-ac. thu. znc.

— — as with an axe: nx-v.

— — — — a finger: nat-m.

— **occiput**: arn. cann-i. hell. lyc. mang. plb. ran-b. sabad.

— — from occiput to forehead: sabad.

— — dull, heavy, throbbing pain through head, with sensation like a heavy blow on back of head and neck: cann-i.

— **side** of: alum. (r), am-c. (l), bov. (r), chel. graph. (r), k-ca. (r), laur. (l), plat. mag-m. (l), nat-s. (l), phos. (r), plb. (r), puls. (r), sars. (r), spig. (l), sulph.
— **temples**: agn. am-c. (r), bar. (l), camph. croc. lach. *lyc.* olnd. (l), ph-ac. plat. spig. (r), sul-ac. thu.
— — as if a peg were struck in deep: sul-ac.
— — sudden, deep in, causes starting: croc. (r).
— **vertex**: calc. lyc. mang. nat-c. phos. tong.
— — as from a bolt, from neck to vertex, worse at each throb of heart: cimic.
— — electric like: nat-s.

**Conditions** of Shocks.
— **morning** in bed: nx-v. sul-ac.
— — on rising: tarent.
— — — — amel.: nx-v.
— — every: sul-ac.
— **ascending**, on: ant-c. arn. bell. men. par. ph-ac.
— **consciousness**, on regaining: cann-i.
— **coughing**, when: ars. calc. ipec. lach. lyc. mag-s. mang. nat-m. rhus. seneg. spig. sul-ac.
— **drinking** cold water, on: thea.
— **eating**, after: lyc.
— **hawking**, on: raph.
— **lying**, when: nit-ac.
— **menses**, during: borax.
— **motion**, from: am-c. *lyc.* merc. prun.
— **pressure**, amel.: bell. thu.
— **pulse**, at each beat of: *cimic.* glon.
— **reading**, from: carb-v.
— **shaking** head, when: mang.
— **siesta**, after a: sep.
— **sitting**, after a full meal, when: lyc.
— **sleep**, on going to: nat-c.
— **sneezing**, when: bar.
— **stool**, during: phos.
— **stooping**, on: merc. nit-ac. thu.
— **talking**, when: nat-m.
— **walking**, when: bell. mang.
— — rapidly: ant-c. arn. bell. par. ph-ac.
— — in open air: spig.
— **writing**, when: raph.

**Shooting** in: acet-ac. agar. alum. ambr. am-c. ant-t. arg. bar. *bell.* bry. calc. caps. carb-v. caus. cham. cimic. colch. con. corn. *ferr.* gran. hell. hepar. hura. hyos. ign. indg. iod. ipec. k-ca. lach. lact. laur. mag-c. manc. marum. merc. mur-ac. naja. *nat-hyp.* nat-m. nit-ac. nitr. nx-v. petr. plan. ptel. puls. rhus-r. sep. sil. staph. sulph. thu. tong. valer.
— — compare Cutting, Lancinating, Shocks, Stitches, etc.
— — declining and increasing: bar.
— — here and there: am-c. bapt. calc. hydr-ac. mag-c. mag-s. nicc. plb. ratan. sul-ac.
— — — flying: asar. calc. stront.
— — from behind forward: nat-m.
— — — within outward: alum. cinnb. nat-c. rhus. sulph.
— — upward: guai. sil.
— **forehead**: acon. agar. ant-t. arn. bell. chin. cinnb. con. creos. cyc. dig. euph. ferr. k-bi. k-ca. merc. merc-i-fl. mosch. naja. plb. rhod. rhus. rumx. sabad. senec. sil. stram. sulph. tilia. tarent.
— — **diagonally**: chel.
— — **flying**: asar. jatr. sep.
— — **inward**: canth. gels.
— — **eyes**, over: am-c. ant-cr. berb. bov. bry. (l), caus. k-bi. nat-m. nit-ac. ph-ac. *sep.* znc.
— — — violent shooting pains from root of nose along left orbital arch to external angle of eye, with dim sight; begins in morning, increases till noon, and ceases toward evening: k-bi.
— — — from left eye to vertex: phyt.
— — **outward**: bar-ac. bell. con. ferr. glon. gran. lyc. ph-ac. puls. senec. sep. sulph. verb.
— — **upward**: ph-ac. scutel.
— **occiput**: acon. æth. agar. ailan. alum. anac. arum-t. asaf. bell. bov. caps. cimic. cedr. glon. grat. hepar. indg. iod. k-ca. laur. lyc. mag-c. marum. men. mur-ac. naja. nat-m. nit-ac. nitr. ol-an. phos. sil. znc.
— — **diagonally** across: agar.
— — **forward**: chel.
— — **upward**: ambr. sep. sil.
— — intense pain as if a bolt had been driven from neck to vertex, worse at every throb of heart: cimic.
— **side** of: acon. æth. agar. aloe. alum. am-c. am-m. anac. arg-n. bar. calc. camph. caus. cham. *ferr.* (l), fluor-ac. k-ca. lach. mang. men. nat-m.

phos. phys. plan. rumx. sabin. sars. stann.
— **temple**: acet-ac. acon. æth. (l), agar. (r), anac. (l), arum-t. bapt. bell. (l), calc-p. (r), cimic. (l), coca. (r), com. (l), cup-s. glon. iris. k-bi. kalm. lil-t. (r to l), merc-i-fl. naja. phos. phys. phyt. pic-ac. pip-m. rhus. (l), sep. (l), sulph (r), sul-i. tarent. (r), thea.
— — spreading out in a circle: *caus.*
— — transient: iris. tarent.
— — from one temple to another: *bell.*
— — **inward**: arn. berb. canth. dirca. rhus.
— — **outward**: bell. dulc. rhus.
— — — out and in: staph.
— — **upward**: chin-s.
— — — up and down: ang.
— **vertex**: acon. æth. agar. alum. am-m. bar. bell. berb. bov. bry. calc. caps. carb-an. carb-v. caus. chel. chin. cimic. cupr. dig. hura. iod. ipec. k-bi. lach. laur. lyc. mag-c. mezer. mill. nat-m. nit-ac. phel. phos. ph-ac. phyt. spig. stram. sulph. tabac. valer. [znc.
— — **across**: lac-ac.
— — **boring** through: sil.
— — **deep**: caps. indg. lyc. ratan. staph. tabac. tong.
— — **drawing** head backward: phel.
— — **extending** to forehead: nicc.
— — **inward**: aloe. lyc.
— — **transient**: indg. mill.

**Conditions** of Shooting.
— **morning**: arum-t. ptel.
— — before rising, aggr.: *nat-hyp.*
— — begins in, increases till noon and ceases toward evening: k-bi.
— **noon**, at: calc-p. sep.
— **afternoon**: *ferr.* plan. sulph. tarent.
— **evening**: bell. tarent.
— — amel. toward: k-bi.
— **night**: tarent.
— **air**, amel. in open: naja.
— **coughing**, on: arn. bry. calc. carb-v. con. mang.
— **cramps**, during: hell.
— **cries**, extorting: sep.
— **eating**, aggr.: sulph.
— **eyes**, on turning up. arum-t.
— **head**, bending back, aggr.: anac.
— — moving, amel.: sulph.
— **lying** down, aggr.: cimic. k-bi.
— **menses**, before: ferr.
— — during: apis.
— **pressure**, amel.: bell. cupr-s.
— **rising** and walking, aggr.: phys.
— **singing**, when: ptel.
— **sitting**, amel.: acon.
— **stooping**, on: bell. creos. nit-ac. sulph. sul-i.
— **teeth**, clenching, amel.: sulph.
— **vexation**, after: mag-c.
— **walking**, when: bell. phys.

**Sinking** feeling: glon.
— — as if something were sinking from occiput, on stooping: k-ca.

**Sleep**, sensation, as if brain were asleep: con. cupr. nat-m.
— — as from want of, see under Confusion, Dullness.

**Smaller**, feels: acon coff. *grat.* pic-ac.
— brain feels smaller than skull: glon.
— — — too far from skull: staph.

**Smarting**: bapt. camph. canth. chin. euphr. glon. ham. rhus. sabin.
— compare Soreness, and also see under Scalp.
— **forehead**: bapt. canth. carb-an. gels. graph. hydr. lach.
— — on touch: graph.

**Smoke**, sensation as of, in: ang. sul-ac.

**Softening** of brain: ambr. lach. nx-m. phos.

**Soreness**, feeling of: agar. aloe. apis. ars. bapt. bry. calc. camph. *canth.* carb-v. *chin.* cinnb. coca. cupr-ars. daph. eup-per. euphr. ferr. *glon.* graph. ham. hydrph. ign. k-bi. lac-ac. lach. lyc. mag-c. merc. *mezer.* mosch. nat-m. nat-p. nit ac. olnd. phos. phyt. prun. raph. rhod. *rhus.* sabad. sep. sil. *spig.* stram. sulph. thea. *znc.* zing.
— — compare with Sensitiveness, and see also under Scalp.
— — **deep-seated**: mosch. plat.
— — feels as if head would fall to pieces, is afraid to shake it: glon.
— — in spots: oxal-ac. sang.
— **forehead**: acon. apis. ars. bapt. bufo. canth. carb-an. *coloc.* gels. glon. hydrs. lach. lil-t. lyc. merc. mur-ac. nat-c. plan. podo. prun. sang. spig. spong. sulph. tellur. znc. ziz.
— — in spots: par.
— — to touch: hepar.
— — every morning from 9 to 1; begins over left eye, then over nose, temple to back of head: mur-ac.
— **occiput**: bry. chel *cimic.* dirc. eup-per. euph. ferr. *glon.* hyos. mezer. nicc. nx-v. pip-m. sabad. spig.

— — as if broken loose from rest of skull: chel.
— — as if a wound were pressed: sabad.
— **side** of: ambr. (r), eup-per. lil-t. (l), mezer. (r), phyt. sulph.
— — as if suppurating: petr.
— — in spots: sulph.
— **temples**: æsc-h. calc-p. (r), cast. cham. (l), coca. cupr-ars. daph. dirc. glon. gymn. (l), merl. nx-m. plan. plb. puls. sang.
— — to touch and from lying on them; warmth amel.: nx-m.
— **vertex**: apis. bov. bry. bufo. cast. chel. cimic. cinnb. ferr. k-bi. k-ca. lac-ac. mag-c. mag-m. olnd. phos. rhod. rhus. sabin. sep. spig. sulph. sul-i. *znc.*
— — as if bruised: glon.
**Conditions** of Soreness.
— **morning**: bov. hepar. mezer.
— — on waking: *cupr-ars.*
— **afternoon**: bufo. sang.
— **evening**: acon. cast. chel. mag-c. puls. *znc.*
— — in bed: plan.
— **night**: coca.
— **breakfast**, after: merc.
— **coughing**, when: coca. spig. znc.
— **head**, on shaking: glon.
— **lying**, on part, aggr.: nx-m. spig.
— **mental** labor, after: daph. prun.
— **motion**, on: CIMIC. glon. merc. nat-[ars.
— **pressure**, from: pip-m.
— — of hat: carb-v.
— — on pillow: cupr-ars.
— **rest**, aggr.: puls.
— **riding** on cars, aggr.: glon.
— **room**, worse in warm: puls.
— **speaking**, when: spig.
— **stooping**, on: bapt. *coloc.* lyc. nicc.
— **touch**, when: dirc. hepar. merc. *mezer.* mosch. nat-m. phos.
— **vexation**, after: MEZER.
— **walking**, when: raph.
— — in open air, amel.: puls.
**Sound**. See Noise.
**Spasmodic** pain: acon. ambr. ang. *arn.* ars. calc. carb-v. chin. colch. coloc. croton. ign. *mosch.* murx. nat-c. nicc. nit-ac. nitr. nx-m. petr. ph-ac. plat. ran-b. rheum. sars. squil. stann. *stram.* stront.
**Splashing** in: asaf. bell. carb-an. hepar. hyos. nx-v. rhus. spig. squil.
— compare with Undulation, etc.
**Splitting**. See Bursting.
**Spot**, pain in small: borax. carb-v. caus. colch. eupi. ferr-m. graph. helon. hepar. hydr-ac. *k-bi. kalm.* lach. lact. lith. nx-m. oxal-ac. phos. plan. psor. ran-sc. ratan. sang. sol-n. spig. sulph. sul-ac. tellur. thu. vinc. znc.
**Sprained** sensation in back of head: psor.
**Squeezing**. See Compressive.
**Stabbing**. See Cutting, Shooting, etc.
**Stepping**. See Sensitiveness, and compare general conditions of head.
**Stiffness**, sensation of: canth. ferr. glon. nat-m. nat-s.
— — extending to nose: lachn.
— in brain, in open air: phos.
— in occiput: anac. calc. ferr. gins. nitr. phos. sil.
— evening in bed: sil.
— must bend head back: nitr.
— motion, on: nat-s.
— — of head, aggr.: colch.
— on waking: anac.
**Stitches**, stinging, etc.: *acon.* æth. agn. aloe. *alum.* ambr. am-c. am-m. anac. *ant-t.* arg. *arn.* ars. asaf. aur. bapt. *bar. bell.* berb. *borax.* bov. *bry.* calc. camph. cann-i. cann-s. canth. *caps.* carb-an. carb-v. castor. *caus.* cham. *chel.* chin. *cic.* cina. cocc. *con.* croc. croton. cupr. cyc. daph. dig. dirc. dulc. elaps. eugen. euphr. eupi. *euon.* ferr. gels. glon. grat. guai. hell. *hepar.* hipp. hydr-ac. hyos. *ign.* indg. iod. *ipec.* k-bi. *k-ca.* lach. lachn. lact. lam. laur. lobel. *lyc. mag-c.* mag-m. *mag-s.* manc. mang. marum. merc. merc-c. merc-i-fl. merl. mill. mosch. *mur-ac. nat-c. nat-m.* nat-s. nicc. *nit-ac. nitr.* nx-m. nx-v. ol-an. op. *par. petr.* phos. ph-ac. plb. plat. *puls.* raph. ratan. rhod. *rhus.* sabad. *sabin. sars.* selen. seneg. sep. serp. *sil.* spig. spong. *squil.* stann. staph. stront. *sulph. sul-ac.* tabac. tarax. tarent. thu. tilia. tong. *valer.* verat. verb. viol-tr. znc.
— — **boring** stitching, at night: am-c.
— — **burning**: ph-ac. rhod.
— — with coldness and trembling, every day at 4 P. M., unbearable stitches: asaf.
— — **deep-seated**: all-c. lach. tabac.
— — **drawing**: creos. mang. sil.
— — **dull**: mag-m. sep. sil.
— — **extending** to chest or neck: nat-m.

— — — to face: rhus. sars.
— — — to malar bone: indig. rhus.
— — **jerking**: *nat-m.* nx-v. puls.
— — **periodic**: calc.
— — **tearing**: berb. coloc. k-bi. merc. phos.
— **brain**, in: agn. *alum.* am-c. bar. bell. bry. calc. cham. cina. colch. cyc. dulc. euphr. gran. *guai.* k-ca. laur. lyc. mill. nat-m. petr. plb. *puls.* sabin. sil. thu.
— **forehead**: acon. æsc-h. alum. am-c. am-m. anac. ant-t. arg-n. arn. asaf. aur. bar. bell. berb. bov. bry. calc. camph. canth. caps. carb-v. caus. cham. chel. chin. cic. cina. cocc. cocc-c. coloc. *con.* cupr. cyc. dig. dros. dulc. euph. euphr. ferr. gels. gins. gran. grat. guai. hell. hepar. hyos. ign. ipec. k-ca. lact. laur. led. lil-t. lyc. mag-c. mag-m. *mang.* men. merc. merc-c. mezer. mosch. mur-ac. nat-c. nat-m. nit-ac. nitr. nx-v. op. petr. phos. ph-ac. plan. *plat.* plb. puls. rhod. rhus. ruta. sabad. sabin. sars. selen. *sep. sil.* spig. spong. squil. stann. staph. stram. stront. *sulph.* sul-ac. tarax. tilia. valer. verat. verb. viol-tr. znc.
— — left frontal eminence: arg-n. mang. nat-c. sars.
— — right frontal eminence: bell. squil.
— — **burning**: thu.
— — **extending** into ear: rhus. squil.
— — — to lower jaw: brom.
— — — to nose: psor.
— — — to occiput: *cham.* phos.
— — **externally**: ang. dig. hell. hepar. *tarax.*
— — **eyes**, over: anac. arum-t. (l), bov. k-iod. manc. nat-m. ol-an. phos. ph-ac. rhus. selen. (l), spig. (l).
— — **nose**, over: agar. berb. camph. chin. k-bi. k-ca. nat-m. nit-ac. psor. ran-b. rhus. sars. sep. sil.
— — **within**, from: colch. sep. sulph.
— **occiput**: acon. æth. aloe. ambr. ant-t. bell. bov. bry. calc. canth. carb-an. carb-v. caus. cham. *chel.* cimic. dig. dulc. euphr. glon. grat. hell. ign. indg. iod. iris. k-bi. k-ca. k-iod. lac-can. laur. mag-c. mag-m. mang. marum. mur-ac. nat-c. nat-m. nit-ac. nitr. petr. phos. puls. ran-b. rhus. samb. sars. sep. spig. spong. squil. staph. stront. sulph. sul-ac. tarax. thu. verat. viol-tr. znc.
— — **burning**: carb-v. staph.
— — **deep-seated**: canth. cop.
— — **extending** across: agar.
— — — to frontal eminence: bar.
— — — to back of chest: eupi.
— — — through ears: puls.
— — — to upper jaw, left side: cham.
— — **pulsating**: cop. hepar.
— — **tearing**: æth.
— **side** of: æth. (l), alum. (r), am-c. (r), am-m. (l), anac. (l), ant-t. asaf. aur. bar. bell. (r), berb. borax. (r), bov. brom. (r), bry. calc. calc-p. (l), camph. (r), cann-s. (l), canth. (r), caps. cast. caus. (r), cham. (r), chel. (l), cic. cinnb. cocc. cocc-c. con. crotal. (l), cupr. (r), cyc. (l), dig, eup-pur. euph. (r), euphr. eupi. (l), ferr. (l), gamb. graph. grat. (r), guai. hyos. hyper. (r), indg. (l), iod. (r), k-bi. (r), k-ca. (r), lach. laur. mag-c. mag-m. mag-s. (r), mang. (l), men. (l), mezer. (l), mill. (r), mur-ac. nat-c. nat-m. nat-s. (l), nicc. nit-ac. (r), nx-v. ol-an. (r), par. (r), petr. phos. ph-ac. plat. (l), plb. (r), puls. ratan. (l), rhod. (r), sars. sep. sil. spig. (l), staph. (l), sulph. tarax. (l), thu. (r), tong. verb. znc. (r).
— — **extending** deep into brain: anac. indg.
— — — to eye: calc. (r).
— — — to face: k-bi. (r).
— — — to forehead: sil. (l).
— — **tearing**: sars. spig.
— — — extending from side to side: carb-v.
— **temples**: acon. æsc-h. æth. agar. (r), aloe. ambr. (l), am-c. (l), am-m. (l). anac. (l), ang. ant-t. apis. apoc. (r), arn. arum-t. asaf. (l), ars. (l), bar. (r), bell. berb. (r), borax. (r), bov. bry. cadm. calad. (r), calc. (l), camph. *cann-i.* (r), canth. carb-an. carb-v. *caus.* cham. chel. (l), chin. cimic. cina. cocc. (l), cocc-c. coff. (r), coloc. cop. crotal. (l), cupr. (l), cyc. (l), daph. dig. dulc. euphr. eupi. (l), ferr. gamb. gran. graph. (l), grat. (r), guai. hell. (l), hepar. (l), hydr. ign. iod. iris. k-bi. (r), k-ca. k-iod. (l), lyc. mag-c. mag-m. mag-s. mang. men. (l), merc-i-fl. merl. mezer. mur-ac. nat-c. nat-m. nit-ac. nitr. nx-m. *nx-v.* ol-an. par. phos. ph-ac. plat. (l), plb. (l), squil. (r), psor. *puls.* ran-b. (l), ran-sc. (l), rheum. rhod. (l), rhus. ruta. sabad. (r), sang. sars. (r), selen. sep. *sil.* sol-n. spong. stann. staph. (l), stront. sulph. tabac. tarax. (l), tarent. therid. thu. (r), verb. viol-tr. znc.
— — awakening at 3 A. M.; spreading gradually over head; chilly: ferr.
— — **alternating** with heat and coldness: borax.
— — — with pressure: tabac.
— — **boring**, amel. on touch: coloc.
— — **extending** to eyes: ant-c. (l), berb. (r).
— — — across forehead: anac. berb. borax. *ferr.* sil. squil. tabac.
— — — inward: acon. arg. arn. lach. rhus. tilia.
— — — outward: berb. calc. nx-m. rhus. sulph.
— — **needle** like, burning stitches: staph.
— **vertex**: acon. æth. alum. am-m. anac. bar. bell. borax. bov. *bry.* calc. caps. carb-an. carb-v. caus. chel. chin. cimic. con. cop. cupr. dig. ferr. hell. hyper. guai. indg. iod. ipec. k-iod. lach. laur. lyc. mag-c. men. mezer. mill. nat-c. nat-m. nicc. nit-ac. nitr. olnd. ol-an. petr. phel. phos. pimp. ph-ac. raph. ratan. ruta. sabad. sars. sep. spig. stann. staph. stront. sulph. tabac. thu. tong. valer. verb. znc.
— — extending to forehead: caps. mezer. nicc.
— — — through whole head, from exposure to sun: bar.
— — — into palate: nat-m.
— — — to pharynx: cham.
— — — to temples: carb-v. phos.
— — paroxysmal: *caus.* chel.
— — in spots: chel. k-bi.
— — within, outward; better when washing, but worse after: spig.

**Conditions** of Stitches.
— **morning**: agar. alum. am-m. arg-n. bry. canth. cham. con. glon. grat. hepar. indg. lyc. mag-c. mag-s. mang. nicc. petr. plb. sars. sil. stront. thu. tilia. tong. verat.
— — at 3 A. M.: ferr.
— — on waking: petr.
— — after rising: bar. *mag-c.* plb. stront.
— **noon**: con. elaps.
— — till goes to sleep: mur-ac.
— **afternoon**: æth. alum. bov. canth. cham. grat. indg. mag-c. nat-c. nicc. ol-an. phel. sars. sep. stront.
— — every day at 4 P. M., with coldness and trembling: asaf.
— **evening**: ambr. bar. bov. calc. canth. carb-an. carb-v. caus. chel. dig. graph. hyper. indg. k-iod. mag-c. mang. mur-ac. nat-c. nat-m. nit-ac. petr. phos. plat. sep. *sil.* stram. stront. sulph. valer. thu.
— **night**: am-c. arum-t. dig. lyc. nat-m. sep. spig.
— — on waking: hepar.
— **air**, in open: *mang.* sil.
— — — — amel.: am-c. sars. *sep.* tabac.
— **arms**, on moving: nat-s.
— **bed**, when in: nat-c. plat.
— **breakfast**, after: *bry.*
— **chilliness**, during: eupi.
— **cold** hand, touch of, amel.: euphr.
— **coryza**, during: cocc-c. k-ca.
— — during stopped: croc.
— **coughing** when: alum. anac. ant-t. arn. *bry.* calc. *carb-v.* caus. chel. cina. con. hyos. k-ca. mezer. nit-ac. phos. ph-ac. ruta. sabad. sulph. sul-ac. verb. znc.
— **descending**, on: merc-i-fl.
— **dinner**, during: znc.
— — after: ant-t. bar. mag-c. phos. *puls.*
— **eating**, after: alum. ant-t. bar. lyc. mag-c. phel. phos. sep sulph.
— **eyes**, on moving: caps. hyper. k-ca.
— **fright**, from least: cic.
— **head**, on moving: caps. hyper. k-ca. nat-m.
— — raising, amel.: k-ca.
— **heat**, after in face: puls.
— — amel.: k-ca.
— — of stove, from: bar.
— — during the fever (stage): asaf. *nx-v.* puls.
— **house**, when in: nat-m. sep.
— **jaw**, moving aggr.: k-ca.
— **laughing**, when: tong.
— **lying** down, when: canth. nat-c. puls. sep.
— — amel.: calc. nit-ac.
— — on affected side amel.: chel. sep.
— — on well side amel.: mag-c.
— **menses**, before: calc-p. ferr.
— — appearance of, amel.: cyc.

— — during: acon. berb. calc. lyc. mang. ratan.
— — after: berb. lyc. nat-m. ol-an. plat.
— **mental** exertion, after: lyc. pimp.
— **motion**, aggr.: agn. ant-t. caps. cham. nitr. ratan. sil. spong.
— — amel.: sulph.
— **nose**, on blowing: mur-ac.
— **odors**, from strong: selen. (1).
— **pressure**, amel.: æth. calad. guai. mur-ac. sil. sulph.
— **reading**, when: carb-v. caus. lyc.
— **respiration**, on deep: ratan.
— **rest**, amel.: calc.
— **rising**, on: agar. calc. (mur-ac. from seat).
— — amel.: ol-an. puls.
— — from stooping, on: hepar.
— **room**, in: am-m. bov. con. selen.
— — amel. in: mang.
— **rubbing**, amel.: canth. phos.
— **scratching**, amel.: plat.
— **sitting**, when: caus. chin. indg. mag-c. phos. ratan. squil. *tarax.*
— **standing**, when: mag-c. plb.
— — still, amel. when: mang.
— **stepping** heavily, when: aloe. *bry.*
— **stooping**, when: am-m. berb. bry. calc. caps. cyc. glon. hepar. mag-m. mur-ac. nicc. puls. staph. sulph. thu.
— — after: aloe. rhus.
— **sun**, from exposure to: bar. selen. (1).
— **talking**, after: agar. nat-m.
— — loudly: sulph.
— **touch**, from: hepar. ipec. spig. staph.
— — amel.: ars. *coloc.*
— **waking**, on: canth. hepar. petr. thu.
— **walking**, when: bry. carb-an. croton. merc. plb. sep. sulph. thu.
— — after: bry. tarax. tellur.
— — about amel.: staph.
— **washing** face, on: cop.
— — better while but worse after washing: spig.
— **weather**, on change of: vip.

**Stomach**, as if rising from: alum. carb-v. con. mag-m.

**Stone**, as if from a: *bell.* cann-s. con. dig. nitr.
— around eyes, like: nitr.
— see also under Heaviness, Pressure, etc.

**Stopped-up**, stuffed sensation: acon. glon. graph. ham. nat-c. sep. sulph. ustil.

**Strange** feeling in: phys. thea.
— compare Confused.

**Strain**, as from a: *ph-ac.* phys.
— sensation as of a, in forehead: hyper. op.

**Striking**, sensation as though brain were, against skull, on motion: *ars.* laur. nx-m. plat. *rhus.* stann. *sulph.* sul-ac.
— compare with Knocks, Looseness, etc.
— strikes head against wall or bed; twitching of eyelids and frontal muscles: mill.

**Stunning**, stupefying pain: acon. æth. agar. am-c. anac. ant-c. arg. arn. *ars.* asaf. asar. aur. bar. *bell.* bov. bry. calc. chin. cic. cina. cinnb. con. croton. cupr. cyc. dros. dulc. gran. hell. *hyos.* iod. iris. k-ca. lac-can. *laur. led.* lyc. mag-c. men. mezer. mosch. nat-c. *nitr.* nx-m. nx-v. *olnd.* op. *phos.* ph-ac. *puls.* rheum. rhod. rhus. ruta. sabad. sabin. samb. sep. sil. *stann. staph.* sulph. tarax. thu. valer. verb. znc.
— — **compressing**: *mosch.*
— — **drawing**: asar.
— — **pressing**: *ant-t.* arg. arn. ars. *asar.* calc. cic. cina. *croton.* cupr. *dros.* dulc. *evon.* hell, *hyos. mezer. ruta. sabad. stann.* sulph. verb.
— — **stinging**: verb.
— — **throbbing**: sabin.
— — **tightening**: asaf. olnd.
— — with coryza, 4 to 8 P. M., worse on stooping; better at rest and in open air: hell.
— — with heat in temples and ears; mouth and lips dry; worse 4 to 8 P. M., rising up or lying down: lyc.
— **forehead**: agar. anac. *ant-c.* arg. arn. ars. asaf. asar. *bell. calc.* cann-s. carb-an. cic. cina. cyc. dros. euph.. fluor-ac. gran. *hyos.* laur. led. mag-c. mang. men. mur-ac. nat-c. nitr. olnd. par. phos. ph-ac. plat. ruta. sabad. sep. stann. staph. tarax. thu. valer. *verb.*
— — over eyes: evon.
— — above nose: *acon.* ant-t. asar. mosch.
— — so violent sweats from anxiety; when walking in open air: ant-c.
— **occiput**: *cann-i.* cina. seneg. sulph.

— **side** of: asaf. (l), daph. (r), dulc. (l), euph. (r), mezer). (l), olnd. (r), sul-ac. (r), verb.
— **temple**: acon. ars. asar. cina. iod. podo. rheum. sabad. *verb.*
— **vertex**: bov. dulc. phos. rheum. valer.
**Conditions** of Stunning pain.
— **morning**: nx-v. rhus.
— — on waking, as from liquor: nitr.
— **evening**: puls.
— **cold**, amel.: puls.
— — aggr.: rhus.
— — with chilliness: puls.
— **coughing**, on: æth. nitr.
— **eating**, after: nx-v.
— **motion**, amel.: puls. rhus.
— **pressure**, amel.: iod. podo.
— **rest**, aggr. when at: puls. rhus.
— — and motion, during: calc.
— **room**, worse in: nat-c. puls.
— **sunshine**, from: nx-v.
**Stupefaction**, sensation of: acet-ac. æsc-h. agar. ant-t. arg-n. apis. ars. asaf. ars. bapt. bov. bry. calc. camph. caus. cham. chin. cina. clem. cob. *cocc. cocc-c.* coloc. con. croc. *dulc.* ferr. graph. hell. helon. hyos. jatr. ipec. laur. *lyc.* mag-m. nat-m. nat-s. nicc. *nx-v.* op. petr. phos. phys. plan. psor. raph. rhus-v. sars. secale. selen. sep. sil. spig. squil. stann. staph. thu. tong. valer. vip.
— compare Confusion, Duliness, etc.
— **forehead**: ars. berb. cina. *coloc.* con. gels. hell. hepar. mosch. staph.
— **temple**: ant-t. plan.
— **occiput**: agar. cupr-s.
**Conditions** of Stupefaction.
— **morning**: acet-ac. agar. chin. cob. graph. sars. squil. thu.
— — on waking: cham. phos.
— — after rising: rhod. sabad.
— **afternoon**: calc. phys. lyc.
— — 11 A. M. to 6 P. M.: ars.
— **evening**: bov.
— **night**: calc.
— — on waking, must rise: psor.
— **air**, in open: cina. *nx-v.*
— **coryza**, during: hell.
— **dinner**, after: coloc. nat-m. nx-v. plan.
— **eating**, aggr. after: *cocc.* morph.
— **mental** exertion, aggr.: petr.
— **motion**, from: *staph.* thu.
— **retching**, amel.: asar.
— **rising**, on: sil.
— — amel. after: phos.
— **somnolency**, with: ph-ac.
— **stool**, during: tong.
— **stooping**, on: nicc. valer.
— **sun**, aggr. in: *nx-v.*
— **vertigo**, during: acon. æth. agar. arn. bar. bell. bov. calc. clem. creos. gels. graph. hell. hydrs. hydr-ac. laur. mill. mosch. mur-ac. op. phos. phyt. psor. sabin. secale. stann. staph. sulph. znc.
— **walking**, when: ipec.
— — open air: ars. cina.
— **warm** room, in: phos.
— **walking** much, when: alumn. *ars.*
— **writing**, when: arg-n.
**Sudden** pains: agar. *bell.* camph. croc. ferr. mezer. morph. phys. *sabin. tabac. valer.*
— — and go, suddenly: *bell.*
— — which decrease gradually: asaf. calc-ac. fluor-ac. puls. ran-sc. sabin.
— — — compare Increasing.
— — during micturition: tabac.
**Sunstroke**: camph. glon. stram. therid. verat-v.
— from having slept in sun: acon. bell.
**Suppuration**, pain as if from: bov. carb-v. nx-v. petr. rhod. stann.
**Surging** sensation, while lying: oxal-ac.
— — amel. on becoming erect: alum.
— — in forehead, like waves rolling up and down: *sep.*
— — from occiput to forehead: cann-i.
**Swashing** sensation in: asaf. *bell.* carb-an. dig. hepar. hyos. nx-v. plect. rhus. samb. spig. squil. viol-tr.
— — see also Undulation.
— **ice**, amel.: plect.
— **motion**, from: plect.
— **shaking** head, on: spig. squil.
**Sweat.** See under External Head.
**Swollen**, distended feeling: æth. agar. am-c. anac. ant-t. apis. *arg-n.* arn. bapt. bar. *bell.* berb. bism. bov. cann-i. caps. cedr. chin-s. cimic. cina. cob. cocc-c. collin. coral. cupr-ac. daph. dig. dulc. gels. gins. GLON. indg. k-iod. lach. lachn. lact. laur. lil-t. lith. mang. meph. merc. merl. nat-m. nx-m. nx-v. op. par. plan. *ran-b.* ran-sc. rhus. samb. sep. spig. stront. sulph. tarax. therid.
— feels elongated: hyper.
— inflated, feels: k-iod.
— compare Enlarged.
— **forehead**: acon. agar. ars. cic. dulc.

hepar. indg. lyc. merc. mezer. nx-v. phos. pip-m. rhus-v. ruta. sep.
— — expanding alternating with contracting: tarax.
— — feels broad and high: cund.
— **occiput**: bry. dulc. pip-m. puls.
— **side**: caus. nx-m. par.
— **temple**: bufo. (r), calc. (r), cham. (l), euph. (l), par. (r).
— **vertex**: all-c.
— on **waking**: ars. samb.
— **walking** in open air: æth. mang.
— **washing**, sensations as if head, face, and hands were swollen; worse after washing; better going into room: æth.

**Tearing**, rending pain: æth. agar. ailan. alum. ambr. am-c. *anac.* ant-cr. arg. *arn.* ars. asar. aur. *bell. berb.* bov. bry. calc. camph. cann-s. *canth.* caps. carb-an. carb-v. cast. caus. *cham.* chel. *chin.* cina. cinnb. cocc. coff. colch. coloc. con. creos. croc. croton. cupr. dig. dros. eupi. ferr. graph. *guai.* hell. hyos. *ign.* indg. iod. ipec. k-bi. *k-ca.* kalm. lach. lam. laur. led. *lyc.* mag-c. mag-m. mag-s. manc. marum. *merc.* mezer. mill. mur-ac. nat-c. *nat-m.* nat-s. nicc. nitr. *nx-v. ol-an.* petr. phos. ph-ac. plat. plb. psor. *puls.* ran-b. ratan. rheum. rhus. ruta. samb. sars. selen. sep. *sil.* spig. squil. stann. staph. stram. stront. *sulph.* sul-ac. tarax. tereb. thu. tilia. tong. viol-tr. vip. znc.
— — **aching**, tearing, jerking: phos.
— — — shooting, tearing: tong.
— — **asunder**: agar. am-m. coff. *mur-ac.* nat-s. op. *puls.* staph. sul-ac. *verat.*
— — — compare with Bursting.
— — **bruised** tearing: bov. merc.
— — **crazy** feeling runs up back: lil-t.
— — **cutting**: bell.
— — **digging**: coloc. spig.
— — **drawing**: am-c. calad. canth. caps. cina. guai. lach. nx-v. ol-an. rhus. sil.
— — — boring, drawing, tearing: carb-an.
— — **extending** to ear: nx-v.
— — — to face: am-m. anac. bry. guai. lyc. sil. squil. thu.
— — — to left eye in paroxysms: nicc.
— — — to nose: lyc. nat-c. nx-v.
— — — to teeth: lyc. merc. staph.
— — — to throat: anac. merc.
— — **flying**: ambr. con. nat-s. selen.
— — **intermittent**: nicc. rheum.
— — **jerking**: agar. anac. *chin.* k-ca. mag-c. marum. mur-ac. pæon. puls. ratan. thu.
— — **lacerated**, as if. See Torn.
— — — morning on rising, rest and warmth amel.; passes off with much yawning: staph.
— — — pain as if brain were clasped by a hand and being torn and twisted: mur-ac.
— — **periodic**: anac.
— — **pressive**: camph. chel. squil.
— — **pulsating**: ars. rhus.
— — **saw**, as if with a: *sulph.*
— — **shooting**: arg. berb. caus. chel. chin. cic. hyos. hyper. phos. sil. sulph. vip. znc.
— — **spots**, in: aloe. lyc.
— — **stinging**: caps. cocc. *ign.* mag-m. *nat-m.* nicc. puls.
— — **twitching**: *chin.* k-ca. sil.
— **forehead**: agar. agn. alum. ambr. am-c. am-m. anac. arg. arg-n. arum-t. asaf. asar. aur. bell. berb. bism. bov. *bry.* cact. calc. camph. canth. caps. carb-an. carb-v. cast. caus. cham. chel. chin. cina. cocc. cocc-c. coloc. con. cupr. dros. euphr. gran. graph. grat. guai. hell. hepar. hyos. ign. indg. ipec. k-bi. *k-ca.* kalm. lachn. laur. *lyc.* mag-c. mag-m. mag-s. mang. merc. merc-i-r. merl. mezer. mur-ac. nat-c. nat-m. nat-s. nit-ac. nitr. nx-v. op. phel. phos. plb. puls. ratan. rhod. sabad. samb. sars. sep. *sil.* SPIG. stann. staph. stront. sulph. sul-ac. thu. tilia. znc.
— — **across**: bry. kalm. lachn. (l to r).
— — **alternating** with pain in arms: sil.
— — **extending** to chest: cham.
— — — to cervical muscles, then to right arm: bry.
— — — down neck, into face and teeth; worse rising, better lying: lyc.
— — — to eyes: mur-ac. nat-c. nat-m. spig.
— — — to nape: berb.
— — — to occiput: bov.
— — — to temple: caus. gran.
— — — to vertex: merc. sil.
— — **eyes**, over: agn. aur. calc. chel. ferr-i. iod. k-ca. k-iod. lach. lyc. mang. (r), merc. (l), mezer. phos. sang. sil. tong.

— — **flying**: ratan. seneg.

— — **nose**, above: agar. ambr. lyc. nat-c. nat-m. tong.

— — **periodic**: cham. plb.

— — **radiating**: lyc.

— **occiput**: acon. æth. agar. ailan. ambr. am-m. anac. arg. ars. aur. bar. bell. berb. bism. bov. calc. camph. canth. carb-an. carb-v. chel. colch. con. cupr. grat. guai. hyos. hyper. ign. indg. k-bi. laur. led. lyc. mag-c. mag-m. mang. merc. merc-c. merl. mur-ac. nat-s. nit-ac. nitr. nx-m. phel. ph-ac. puls. ran-b. sabad. sep. sil. spig. squil. stann. stront. sulph. tarax. thu. verat. znc.

— — **burning**: cupr.

— — **extending** to forehead: ambr. aur. carb-v. chin. merc.

— — — to nape of neck: berb. nx-m. ran-b.

— — — to temple: anac. arn.

— — — to throat, amel. on rubbing: laur.

— — — to vertex: mag-m. ratan.

— — — forward: anac. aur. chin. merc.

— — — upward: berb. ol-an. sars.

— — — — and forward: ambr. mag-m. ratan.

— **side** of: æth. agar. alum. (r), ambr. am-c. am-m. (r), *anac* (r), ant-t. (l), arg. (l), arg-n. ars. (l), aur. (l), bar. bov. (r), bry. canth. caps. *carb-an.* (r), carb-v. cast. (r), caus. *cham.* chin. (r), cic. (r), cina. (l), cocc-c. colch. (l), coloc. (l), croc. (l), dig. (l), *gran.* (r) graph. (l), grat. (r), *guai.* (l), hell. ign. (r), indg. (r), k-ca. (l), laur. (l), led. (l), lyc. mag-c. (r), mag-m. mang. marum. *merc.* (l), *merl.* (l), mill. (r), mur-ac. nat-c. (l), nat-s. nicc. nx-v. (r), ol-an. phel. (l), phos. (r), plb. (r), *puls.* (r), ratan. (l), rhod. (r), sars. selen. (l), sep. (l), sil. spig. (l), stann. (r), stront. (r), sulph. sul-ac. (r), thu. (l), tilia. (l), tong. verb. (r), *znc.*

— — **drawing**: bov. *caps.* znc.

— — **extending** to eye: *mag-m.* (r).

— — — from side to side: clem. rhus.

— — — to teeth and glands of throat: graph.

— — — down neck, into face and teeth; worse rising, better lying: lyc.

— **temple**: acon. (l), æth. (r), agar. (r), agn. ailan. alum. ambr. (l), am-c. am-m. (l), anac. (l), ant-t. (l), *arg.* arg-n. *arn.* (l), arum-t. (r), asaf. (r), *asar.* (l), aur. (l), bell. berb. bism. (r), bov. bry. (r), calc. calc-ac. camph. (r), canth. (r), carb-v. cast. caus. (r), cham. chel. (r), chin. (l), chin-s. cic. (l), cina. (l), cocc. colch. (r), coloc. (r), con. cop. creos. cyc. dig. (r), dulc. (l), gran. (r), grat. (l), guai. (l), ham. hell. hyper. (r). indg. (r), iod. k-bi. (l), k-ca. (l), k-iod. lach. lachn. (l), lact. (r), laur. (r), led. lyc. mag-c. mag-m. (l), mag-s. (l), mang. merc. merl. mezer. mur-ac. (r), nat-c. (r), *nat-m.* nat-s. (r), nicc. (r), nitr. nx-m. nx-v. olnd. ol-an. (l), par. petr. phos. ph-ac. (l), plb. plect. puls. ran-b. (r), ratan. (l), rhod. (l), rhus. (r), sabad. sabin. samb. (l) seneg. (r), sep. (l), sil. (r), *spig.* (l), spong. stann. sulph. (l), sul-ac. thu. tilia. (l), verb. viol-tr. (l), znc.

— — **extending** into brain: anac.

— — — to face: am-m. arg-n. bry. k-ca. lachn. seneg.

— — — across forehead: cast. lyc. mezer. ph-ac.

— — — to neck: bry.

— — — to occiput: rhus-v.

— — — to teeth: bry. carb-v. lachn. *verb.*

— — — upward: am-m. laur. rhus-v.

— — in **spot**: carb-v. ratan.

— — twitching-tearing in temple lain on; moves to other side on turning; worse evenings and raising eyes: puls.

— **vertex**: agar. agn. ambr. am-c. arg. *aur.* bar. *bell.* benz-ac. borax. bov. canth. cast. chel. colch. creos. dulc. hyper. indg. iod. k-ca. kalm. lach. lachn. laur. *lyc.* mag-c. mag-s. mang. merc. mezer. mur-ac. naja. nit-ac. nx-v. phel. phos. ph-ac. ran-b. ran-sc. ratan. rhus. ruta. sars. sil. spig. stann. thu. vinc. znc.

— — **extending** to ear: agar. phos.

— — — to occiput: indg.

— — — to shoulder: lyc.

— — — over temples: ang.

— — — to zygoma, while sitting: phos.

— — **hair** were pulled, as if: canth.

**Conditions** of Tearing.
— **morning**: alum. arg-n. borax. bov. *coloc.* con. indg. mezer. nicc. ran-sc. rhod. sars. sil. tong. verat.
— — on waking: graph. phos. puls. verat.

— **morning**, in bed: arg-n.
— — on rising: ipec. staph. stront. stram.
— **noon**: graph. znc.
— — every day at; pressure aggr., open air amel.: arg.
— **afternoon**: æth. calc. cast. caus. chel. creos. graph. grat. guai. k-iod. laur. lyc. mag-c. mag-s. nat-c. nicc. ol-an. sil. sulph. znc.
— — till evening: lyc.
— **evening**: ailan. almn. ambr. am-c. coloc. grat. hyper. lachn. lyc. mag-c. mag-m. merc. nicc. nitr. olnd. petr. puls. sars. sil. staph. sulph. sul-ac.
— — in bed: laur. sil. thu.
— — till midnight: laur.
— **night**: cham. hepar. laur. lyc. mag-c. merc.
— — on waking: arg-n.
— **air**, from cold: ign.
— — in open: alum. ant-t. mag-s. mang. ol-an.
— — — — amel.: aur. sulph.
— **bed**, amel. in: aur.
— — warmth of, aggr.: merc.
— **breakfast**, during: sul-ac.
— **chill**, during: eupi. hyper.
— **cold**, aggr.: bov. grat. stram.
— **cough**, during: alum. arn. calc. cupr-ac. puls. sep.
— **dinner**, during: znc.
— — after: carb-an. mag-c. ol-an. znc.
— **eating**, while: con. sul-ac. znc.
— — after: carb-an. mag-c. ol-an. phel. sep. znc.
— **eyes**, on moving, aggr.: dros. mur-ac.
— **head**, bending back aggr.: anac.
— — — forward, amel.: ign.
— — — — burning tearing in, on: cupr.
— — moving, aggr.: *coloc.*
— — resting on hands, amel.: dros.
— — — on table, amel.: sulph.
— **heat**, during the: puls.
— — amel.: staph. stram.
— **knitting**, when: mag-s.
— **labor**, from hard: *anac.*
— **lying** down, when: mag-c.
— — on back, amel.: ign.
— — must lie down: colch.
— — lying quietly, towards morning, amel.: merc.
— **menses**, during: calc. cast. mag-c. nat-c. ratan.
— — before: ars. cinnb.
— **mental** exertion, from: *anac.*
— — — amel.: calc-ac.
— **motion** aggr.: *agn. aur.* calc. canth. chin. phos. ratan. sil. SPIG. verat.
— — amel.: mur-ac. sulph.
— **noise**, aggr.: SPIG.
— **pressure** aggr.: arg. bism.
— — amel.: calc-ac. carb-an. mag-c. nat-c. sulph.
— **respiration**, on deep: ratan.
— **rest**, amel.: staph.
— **rising**, on: am-m. kalm.
— — from stooping: ant-t. mang.
— — up in bed, aggr.: mur-ac.
— — — — amel. on: tong.
— **room**, on entering: mag-m.
— — amel. in: ol-an.
— **rubbing** amel.: laur.
— **sitting**, while: indg. mag-c. mezer. nicc. phos. *spig.*
— — amel.: mag-m.
— — upright, aggr.: ign.
— **standing**, while: ran-b. SPIG.
— — still, amel.: *tarax.*
— **step**, a false, aggr.: SPIG.
— **stooping**, on: asar. bov. carb-an. rhus. sil. tong.
— **talking**, on: sars.
— **touch**, aggr.: arg. chel. ipec.
— — amel.: mur-ac.
— **vomiting**, after: thu.
— **waking**, on: arg-n. graph. phos. thu. verat.
— **walking**, when: cast. chin. con. sars. SPIG. tarax.
— — in air, amel.: ant-c.
— **water**, cold aggr.: sulph.
— **writing**, when: ran-b.
— **yawning**, passes off with much: mur-ac.

**Tender.** See Sensitiveness, Soreness, etc.

**Tension**: æth. aloe. alum. ambr. ang. ant-t. *arn.* ars. asaf. asar. bapt. bar. bell. berb. bov. bry. calc. cann-s. carb-an. carb-v. *caus.* cham. clem. coff. colch. con. corn. creos. crotal. dig. dios. glon. *graph.* gymn. hell. hepar. ipec. lach. lact. laur. lobel. *lyc.* mag-c. mag-m. mang. men. merc. merl. *mosch.* mur-ac. nat-c. nat-m. nit-ac. nitr. *nx-v.* olnd. op. oxal-ac. par. petr. *plat.* puls. rheum. rhod. sabad. samb. *sil.* spig. stann. *stront.* sulph. sul-ac. therid. valer. verat. verb. viol-od. xanth. znc. ziz.
— — compare Compressive, Pressure.

— — **bandage**, as from a: chel. gymn.
— — — ligature around, as from: bell.
— — **choking** sensation, followed by a: glon.
— — **skull** were too small, as if: glon. morph. scut.
— — **thread** were stretched from nape to eyes: lach.
— — — were stretched through eyeball back into middle of brain: par.
— — **water** were in, as if: samb.
— **forehead**: acon. æth. agn. aloe. *ant-t.* asaf. bapt. bar. bell. berb. calc. cann-s. carb-an. caus. chel. chin. clem. colch. coloc. croc. crotal. croton. dig. dros. dulc. euph. gent-l. glon. grat. hell. hepar. hyper. laur. mag-m. marum. men. merc. mosch. naja. nat-c. nitr. nx-v. par. plat. puls. rheum. rhus. ruta. sabad. sabin. sep. *sil.* spig. *sulph.* valer. verb. verat. viol-od. znc. ziz.
— — **across**: iris. naja.
— — **skin** of: bar. cann-i. op. par. phys. sabin. sep. verat.
— — **skull** were too tight, as if: naja.
— — **string** or band, across, as from: chel. coca. manc. merc.
— — over eyes: apis. chel. dulc. euphr. glon. (r), iod. merl. sil. sul-i.
— — over nose: æth.
— **occiput**: agar. alum. anac. bar. berb. cann-s. *chel.* coloc. dulc. euph. glon. *graph.* hyos. ipec. k-iod. lact. lobel. lyc. mag-c. mezer. mosch. mur-ac. murx. nat-c. par. ruta. thu. viol-od. verat. ziz.
— — **alternating** with tension in face: *viol-od.*
— — extending to finger joints: plect.
— — — into nape of neck: pimp.
— — — upward, downward, and toward ears: glon.
— **side** of: ant-t. (l), apis. (l), asaf. (l), bar. (r), calc. (r), caus. (l), chin-s. clem. (r), coloc. (l), dig. (r), fluor-ac. (r).
— — extending into orbits and teeth: crotal.
— — — to left upper teeth, stitch-like in spots, on stooping; amel. on rising: dig.
— **temple**: ailan. alum. (r), am-bro. ant-t. bar. berb. (l), bov. calc. *cann-s.* caus. (r), *carb-an.* cinnb. clem. coloc. (r), glon. hell. hyper. lith. lyc. mag-m. merl. mur-ac. (r), nat-m. (r), ol-an. pallad. *plat.* rheum. verat. verb. znc.
— — like a band from temple to temple: carb-ac.
— **vertex**: ant-t. apis. cact. calc. chel. gent. k-iod. men. mosch. naja. rheum. stront. tong. verat. verb.
— — extending to jaw: stront.

**Conditions** of Tension.
— **morning**: agar.
— — amel. in: glon.
— **evening**: asaf. lobel. murx. mur-ac. ol-an. stront.
— **night**: mezer. nx-v.
— **air**, open, amel.: berb. lach.
— — walking in, amel.: oxal-ac.
— **bed**, evening in: asaf. merc. ol-an.
— **eating**, after: con. dios. lyc.
— **eyes**, on turning to side: dig.
— — using the, aggr.: par.
— **laying** forehead on table, when: ang.
— **lying** on back, when: mezer.
— **menses**, before: sil.
— **mental** exertion, from: par. *sulph.*
— **motion**, aggr.: par.
— **pain** in occiput, with: graph.
— **pressure**, amel.: lach.
— **throat**, after tightness about the: glon.
— **rising** from stooping, amel.: dig.
— — from bed and laying hand on forehead amel.: merc.
— **sitting** bent forward aggr.; sitting erect amel.: asaf.
— **sleep**, aggr. on going to: graph.
— **standing**, aggr.: mag-c.
— **stool**, during: coloc.
— **stooping**, on: berb. dig.
— **swallowing**, on: mag-c.
— **waking**, on: anac. *ant-t.* graph.
— **writing**, when: *lyc.*

**Thick**, feels: ailan. clem. colch. nat-m. nicc. petr. ran-sc. thea. therid.
— compare with Swollen.
— **forehead**: mag-s. ruta. spong.
— **temple**: gins. (l).

**Thin**, cranium feels as if: bell. puls.

**Thread-like** pain over eyes: all-c.
— tension as from a thread through eyes: lach. par.

**Throbbing**. See Pulsation.

**Throwing** head about: merc. phos. tarent.
— — backward: acet-ac. camph. cina. ether. *glon.* hell. lobel. merc. nitr. phyt. *stram.* tabac. tanac.

— — — boring into pillow: *bell.* camph. dig. hell. hyper. *stram.*
— — — during sleep: hyper.
— — from **side** to side: ars. *bell.* hell. merc. naja.
— — — — while lying: œna.
— — compare Motions of, Rolling, etc.
**Thrusts**. See Jerks, Shocks, etc.
**Ticking** in. See under Noise.
**Tickling** in: ferr. phos.
— — brain: laur. phos.
— — — night: hyper.
— — **forehead**: brom. ferr.
**Tied**, feels as though: colch. pimp.
— see Compressive, Tension, etc.
**Tightness** in head: caus. rheum.
— tightening pain: berb.
— see Tension.
**Tingling**: acet-ac. acon. am-c. apis. arg. arn. bar. cadm. caus. chel. cic. cocc. *colch. cupr.* hyos. laur. nx-m. phos. ph-ac. plat. puls. rheum. *rhus.* secale. sulph. tarax. thu. verb.
— — as though a large bell were struck: sars.
— — compare Crawling.
— **forehead**: arn. aur. chel. cic. *colch.* indg. ph-ac. puls. sabad. stram. tarax. verat. viol-od. viol-tr. znc.
— **occiput**: rhus.
— — stupefying, on stepping: sulph.
— **temple**: borax. plat. rheum. stront. sulph.
— — with coldness of spot: plat.
— **vertex**: calc. colch. cupr. hyos. lac-can. sulph.
— — strange tingling pain in; menses omitting: cupr.
— on speaking aloud: znc.
— while walking: verb.
**Tired** feeling in brain: apis. *con.*
— pain, after mental exertion: iris.
**Torn**, pain as if: agar. alum. am-m. ang. ars. aur. bell. bov. camph. carb-an. caus. cham. *chin.* coff. con. euphr. ferr. graph. hell. hepar. ign. ipec. iod. lach. mag-c. merc. mosch. *mur-ac.* nicc. nitr. nit-ac. *nx-v.* op. phos. ph-ac. plat. puls. rhus. sep. stann. *staph.* stront. sulph. thu. verat. znc.
— — compare with Bruised, Tearing, etc.
— **brain** aches as if torn to pieces, morning on rising, worse from motion, better from rest and warmth; passes off with much yawning: staph.
— — pain as if brain were torn or beaten to pieces: worse moving eyes or sitting up in bed; better from moderate exercise: mur-ac.
— — pain as if brain were clasped by a hand and were being torn and twisted: mur-ac.
— **forehead**: am-m. asar. coff. graph. mezer. nx-v.
— **occiput**: con.
— **side**: nx-v. sulph.
— **temple**: mur-ac.
— **vertex**: carb-an. caus. mur-ac. thu. znc.
**Torpid** feeling: hura.
— see Numbness, etc.
**Trembling** sensation: alcoh. ant-t. bell. bufo. calc. carb-v. chel. *cic.* cocc. graph. merc. plb. sulph. tabac.
— extending to pit of stomach: phys.
— in head: indg. petr. plat.
— on coughing: ant-t.
— moving aggr.: cic.
**Turning**, twisting in head (sensation of): bell. bry. calc. indg. iris. k-ca. petr. rhus. sabad. sil.
— **backward** of: laur.
— to **side**: op.
— — side to side: colch. hyos. phos phys. secale. tarent.
— — — see Rolling.
— — to left side: lyc. tarent.
— — to right side: plb.
— — to wrong side, when spoken to: *atrop.*
— **to and fro**: *agar.*
**Twitching** in: ambr. apis. arn. bell. bry. calc. cann-s. carb-v. caus. chel. chin. croton. cyc. eupi. glon. graph. ign. k-ca. laur. lyc mag-c. merc. nat-c. nit-ac. nx-v. petr. phos. ph-ac. rhus. sabad. sep. sil. stann. staph. stram.
— in **brain**: bar. bov. bry. calc. cann-s. ratan.
— **forehead**: acon. agar. ant-t. arn. berb. borax. bry. caus. cham. chin. lach. mag-m. mezer. phos. *prun.* sabad. *sep.* sil. spong. stann. sulph. thu.
— — extending into brain: camph.
— **occiput**: acon. bism. canth. mag-c. mag-m. merc. ph-ac. rhus. sars. *spig.* thu.
— — extending to forehead: anac.
— **side** of: æth. (r), agar. (r), anac. (l), ang. (r), bar. (r), calc. (l), cann-i. (l), caus. (r), cupr. (l), glon. graph. nit-ac. (l), oxal-ac. (r), plb. (r), valer. (r), verb. (l).
— — extending from side to side: merc.
— — — to throat: chin.
— — — to vertex, when jerking arms and on stepping: spong.
— **temple**: agar. am-c. am-m. (l), anac. (l), arg. bar. (l), bov. (l), bry. carb-an. chel. (l), chin. crotal. cyc. k-ca. (l), lil-t. merc. (r), oxal-ac. phos. (l), plb. *spig.* squil. (r), stann. (l), sul-ac. (r), valer. (r).
— — extending into brain: camph.
— — — to jaws or teeth: rhus.
— — — to vertex: cyc.
— — in spots: ratan. (r).
— — twitching-tearing in temple lain on; moves to other side on turning; worse on raising eyes: puls.
— **vertex**: chel. gent. mag-c. men. mur-ac. petr. ran-sc. sil.
**Conditions** of Twitching.
— **morning**: glon. nx-v. phos. sep.
— **noon**: glon.
— **afternoon**: æth. borax. rhus.
— **evening**: fluor-ac. mur-ac. nit-ac. rhus. sil.
— **night**: chel. rhus. sil.
— **arms**, when jerking the: spong.
— — on moving: chel.
— **ascending** steps, on: glon.
— **cough**, during: lyc puls.
— **eating**, after: cham.
— **lying** down, when: nit-ac.
— **motion**, aggr.: phos.
— **rest**, amel.: eupi.
— **standing**, evening, after: fluor-ac.
— **stepping**, when: spong.
— **stool**, aggr. during: phos.
— **stooping**, on: berb. nit-ac. petr.
— **touch**, aggr: chel.
— **walking**, while: *spig.*
**Twitching of** head: aloe. arn. cham. cic. merc. nat-s. petr. ph-ac.
— — Compare with Rolling, etc.
— — during sleep: arn. merc.
— — on walking: petr.
— **backward**, of: alumn. atro. bov. cic. cina. merc. sep. strych.
— — during sleep: hyper.
— **forward**: merc. sep. strych.
— **side** to side: k-ca. plb.
**Ulcerous** pain: acon. am-c. borax. *bov.* carb-v. cast. caus. creos. hepar. k-ca. mag-c. mang. merc. nx-v. puls. sep. stront. sulph.
— **forehead**: hepar. mur-ac. nx-v.
— — periodical, with constipation: nx-v.
— **occiput**: am-c. mang. nx-v. sep.
— **temple**: mur-ac. *puls.*
— **vertex**: cast. znc.
**Undulating** pain: ant-t. asaf. chin. cocc. ferr. plat. spig. sep. spig. viol-tr. znc.
**Undulation**, waving sensation: acon. alum. caus. chin. cimic. cina. cupr-s. dig. dulc. ferr. glon. graph. hepar. hyos. indg. *lach.* laur. lyc. mag-m. mang. merc. mill. par. petr. sars. selen. sulph.
— — as from water in: asaf. bell. cina. dig. ferr. mag-m.
— — wave-like motion upward: glon.
— **forehead**: asaf. *bell.* merc. petr.
— — from right to left: glon.
— — like a heavy body swaying back and forth: op.
— **motion** amel.: petr.
— **standing** aggr.: dig.
— **turning** head, aggr.: glon.
**Unsteady**, feels: bell. clem. phos. rhus. sep. *sulph.*
— — aggr. after study: cupr-ars.
**Vacant** feeling: secale.
— forehead, morning after waking: sulph.
**Vapor** in: nx-m.
— of coal, as from: am-m. nit-ac. znc.
**Vertigo.** See Vertigo in general.
**Vibration**: arn. am-c calc. grat. indg. k-ca. lyc. mag-c. nit-ac. nx-m. nx-v. sars. sil. stann. stront. sulph. verb. znc.
— — see also Motion, Undulation.
— — as of a steel spring: grat.
— forehead: *merc.*
— temple: k-ca. stront.
— occiput: sulph.
— **coughing**, on: hepar.
— **motion**, from slight: arn. mag-c.
— **stepping**, when: lyc. nx-v. sil.
— **talking**, when: phos. verat.
— **walking**, when: verb.
**Violent** pains: æth. apis. ars. bell. cann-s. canth. cimx. cina. cinnb. coloc. croc. crotal. cupr. euphr. grat. hell. hyos. lach. laur. lyc. mag-c. merc. mosch. nat-m. op. plb. sil. stram. tarax. therid.
— — during the climacteric: therid.
**Vise.** See under Compressive.
**Wagging**: bell, cham.

— see also Shaking.
**Wandering** pains: am-c. chin. k-bi. led. lyc. nat-s. podo. puls.
— mist before eyes; then fleeting pains, worse at occipital protuberance, down neck and shoulders, better lying in a dark, quiet place, and from sleep: podo.
**Water**, sensation as of, dripping on head: cann-s.
— — **brain**, on: acon. am-c. apis. apoc. *aur.* bell. calc-p. dig. hell. indg. lach. mag-m. ph-ac. plat. samb. stram.
— — of boiling water in: acon. indg.
— — of warm, in: am-c. petiv. sant.
— — wrapped up in, as if: all-c.
**Waves** waving. See Undulation.
**Weakness**: alum. *ambr.* ant-t. asaf. aur. bell. bry. canth. carb-v. caus. cham. chin. cinnb. creos. hepar. hyper. k-ca. *merc.* nat-m. nit-ac. nx-m. op. phos. ph-ac. plan. psor. ran-b. raph. rhus. sep. spong. squil. stann. stram. sulph. sul-ac. tabac. tanac. tarent. thu. znc.
— compare Dullness, etc.
— disabling: iod.
— on side on which lies: mag-m.
— as from working in hot room: glon.
— as though headache were coming on: ambr. iod. lac-can. phos. stram. thu.
— extending to lower extremities, as though paralyzed: phys.
— — to throat: graph.
**Conditions** of Weakness.
— **morning**: cham. phos. ran-b.
— — after rising: ph-ac.
— **noon**: ars.
— **evening**: plan. raph.
— **coffee**, after: *cham.*
— **cough**, after: hepar.
— **dinner**, during: sulph.
— **exertion**, after: hydr-ac.
— **heat**, after: sep.
— **lying** on back, when: puls.
— **mental** exertion, after: cinnb.
— — causes mental weakness: spong.
— **pain**, after: thea.
— **respiration**, on deep: carb-v.
— **standing**, while: rhus.
— **stomach**, during weakness in: ars.
— **walking**, when: sulph.
**Weariness**: lach. nat-m. nx-m. *psor.*
— see also Tired.
**Wedge**. See Plug, Nail, and under Pressure.
**Weight**. See Heaviness, Pressure, etc.
**Whirling** in: arg. ars. eup-per. fluor-ac. glon. lach. nx-v. petr. sabad. sep. sil. sulph. tarax.
— — early morning: eup-per.
— — like a mill wheel: chin-s.
**Whizzing**: k-ca. lact.
— as of boiling water, in side lain on: mag-m.
**Wild** feeling: acon. agar. ambr. am-c. *anac.* ant-c. ars. asaf. asar. bell. bov. bry. *carb-v.* chin. chin-s. coloc. croc. euphr. ferr. gels. graph. grat. hell. ign. lact. led. *lil-t.* lyc. *merc.* mezer. nat-c. *nx-v.* phos. ph-ac. *puls.* rhod. sabad. secale. seneg. spig. squil. staph. thu.
— — alternating with uterine pains: gels.
— — crazy feeling on top of head; wild feeling in head, with confusion of ideas: lil-t.

